# Chemical Composition Tables of Locally Available Ruminant Feeds in West Africa: A Systematic Review

**DOI:** 10.3390/ani16081215

**Published:** 2026-04-16

**Authors:** Alassan Seidou Assani, Myriam Koudjoué, Hilaire Sanni Worogo, Mirabelle Jésugnon Houngbedji, Nouroudine Alimi, Loukaiya Zorobouragui, Yaya Idrissou, Ibrahim Alkoiret Traoré

**Affiliations:** Laboratory of Ecology, Health and Animal Production (LESPA), Faculty of Agronomy (FA), University of Parakou (UP), Parakou P.O. Box 123, Benin; koudjouea@gmail.com (M.K.); hilairov@gmail.com (H.S.W.); houngbedjimirebelle@gmail.com (M.J.H.); nouroudinealimi@gmail.com (N.A.); louckzoro@gmail.com (L.Z.); yayaidriss2617@gmail.com (Y.I.); alkoiretib@yahoo.fr (I.A.T.)

**Keywords:** feed chemical composition, ruminant nutrition, agro-industrial by-products, feed variability, West Africa

## Abstract

Feed availability and quality remain major constraints to ruminant productivity in West Africa, where livestock systems rely heavily on locally available resources such as natural forages, crop residues and agro-industrial by-products. However, reliable ration formulation requires accurate information on feed chemical composition, while existing data are fragmented and highly variable. This study conducted a systematic review of peer-reviewed literature published between 2000 and 2025 to synthesize available data on the chemical composition of ruminant feeds in West Africa. Following PRISMA guidelines, 44 studies reporting quantitative feed composition data were retained. Feed resources were classified into agro-industrial by-products, agricultural by-products and forages, and descriptive statistics were calculated for key nutritional parameters. The results revealed substantial variability in nutrient composition across feed types and even within the same feed resource. Cottonseed cake emerged as a major protein-rich supplement, legume haulms showed higher nutritional value than cereal residues, and several browse species such as Moringa oleifera and Leucaena leucocephala demonstrated high protein potential. These findings highlight that fixed feed composition values are poorly suited to heterogeneous tropical feeding systems. The reference ranges established in this review provide a more reliable basis for feed evaluation and ration formulation and can support the development of locally adapted feeding strategies and decision-support tools for West African livestock systems.

## Simple Summary

Livestock production in West Africa relies heavily on locally available feed resources such as natural pastures, crop residues and agro-industrial by-products. However, the nutritional quality of these feeds varies widely depending on environmental conditions, plant maturity and processing methods. Because of this variability, farmers and livestock nutritionists often formulate rations using incomplete or inaccurate information, which can reduce animal performance. This study compiled and analysed published data on the chemical composition of feeds used in West African ruminant systems. The results revealed large variations in nutrient content among feed resources. By providing realistic reference ranges rather than single values, this review offers a more reliable basis for feed evaluation and ration formulation, helping to improve livestock feeding strategies and productivity in West Africa.

## 1. Introduction

Livestock production plays a central role in food security, rural livelihoods and national economies across West Africa. The sector contributes approximately 25% of the agricultural gross domestic product in sub-Saharan Africa and represents a major source of animal protein and income for rapidly growing populations [1,2]. Ruminant production systems, largely based on extensive and semi-intensive management, provide income and nutrition for millions of smallholder households. However, animal productivity in these systems remains relatively low, largely due to chronic feed shortages and poor feed quality, particularly during the prolonged dry seasons that characterize many West African agro-ecological zones [3]. These constraints are exacerbated by increasing climatic variability, which affects pasture availability and feed resource stability. As a result, feed scarcity remains one of the most critical limitations to livestock productivity, herd resilience and farm income in the region [4].

To cope with seasonal feed deficits, livestock keepers rely on a wide diversity of feed resources, including natural pastures, crop residues, agro-industrial by-products and a range of non-conventional feeds [5,6]. Efficient utilization of these resources requires accurate knowledge of their chemical composition in order to formulate balanced rations capable of meeting maintenance, growth, production and reproductive requirements [7,8]. However, ration formulation in many West African livestock systems still relies on fragmented information, empirical practices or feed composition tables originally developed for temperate production systems. These tables may inadequately represent tropical feed resources because of differences in plant physiology, higher fibre lignification, variable secondary metabolites and strong agro-ecological influences on feed quality [9,10].

West Africa encompasses a wide range of agro-ecological conditions, from Sahelian drylands to humid coastal zones of the Gulf of Guinea, resulting in substantial variability in the type and quality of feed resources available to livestock [4,11]. Numerous studies have reported the chemical composition of feeds used in these production systems [5,12,13]. Nevertheless, reported values for the same feed resource often vary widely across studies due to differences in botanical origin, soil fertility, climatic conditions, harvest stage, processing methods and analytical procedures [14,15]. For instance, crude protein content of browse forages or crop residues reported in the literature may vary by more than 20% between studies conducted in different agro-ecological zones. Such variability complicates the use of existing data for practical feed evaluation and ration formulation, particularly for smallholder farmers who depend heavily on locally available feed resources.

In addition to conventional feeds, livestock producers increasingly explore alternative feed resources to improve feed availability and reduce feeding costs. These include non-conventional feeds such as tree leaves, agro-industrial residues and emerging resources such as microalgae or aquatic plants like *Azolla pinnata*, which have shown promising nutritional value and potential for sustainable livestock feeding [16,17]. However, reliable information on the chemical composition of many of these resources remains scattered across individual studies, limiting their integration into feeding strategies.

Existing feed composition tables rarely capture the full diversity of feed resources used in West African livestock systems, particularly locally available by-products and non-conventional feeds that play a critical role in smallholder production systems [18]. Moreover, although some feed evaluation systems provide variability ranges, these are generally derived from temperate production environments. Consequently, existing feed tables rarely provide region-specific variability ranges under tropical agro-ecological conditions, where feed composition is strongly influenced by environmental and management factors [8,19]; This lack of harmonized and regionally relevant reference ranges constrains both scientific research and the development of context-specific feeding strategies adapted to West African livestock systems.

Against this background, a systematic synthesis of published data is needed to consolidate existing knowledge, quantify variability and establish realistic reference ranges for the chemical composition of livestock feeds used across major West African production systems. In order to improve the robustness of the synthesis and reduce the influence of isolated or potentially biased observations, only feed resources reported in at least two independent studies were considered in the analysis. The objective of this study was therefore to conduct a systematic review of peer-reviewed literature published between 2000 and 2024 to compile and harmonize chemical composition data for feeds commonly used in West African livestock systems. By generating reference tables including mean, minimum and maximum values for key nutritional parameters, this work aims to support improved feed evaluation, ration formulation and sustainable ruminant nutrition strategies adapted to the diverse agro-ecological conditions of West Africa.

## 2. Materials and Methods

### 2.1. Literature Search Strategy

A systematic literature review was conducted to compile published data on the chemical composition of feed resources used in ruminant production systems in West Africa. The review followed the Preferred Reporting Items for Systematic Reviews and Meta-Analyses (PRISMA) guidelines to ensure transparency and reproducibility of the study selection process [20]. Peer-reviewed articles were retrieved from three scientific databases: Scopus, Web of Science and Google Scholar. The literature search covered the period from January 2000 to December 2025 in order to compile a comprehensive dataset on feed resources used in ruminant production systems in the region.

The geographical scope of the review was restricted to West African countries, defined according to the Economic Community of West African States (ECOWAS) classification: Benin, Burkina Faso, Cape Verde, Côte d’Ivoire, Gambia, Ghana, Guinea, Guinea-Bissau, Liberia, Mali, Niger, Nigeria, Senegal, Sierra Leone and Togo. Searches were conducted using combinations of keywords related to feed composition and ruminant feeding systems in both English and French. The search string included: ((“chemical composition” OR “nutritional value” OR “feed analysis” OR “feed composition”) AND (“ruminant” OR “cattle” OR “sheep” OR “goat*” OR “livestock”) AND (“locally available” OR “local” OR “regional”) AND (“feed” OR “forage” OR “diet”) AND (“Benin” OR “Burkina Faso” OR “Cape Verde” OR “Côte d’Ivoire” OR “Gambia” OR “Ghana” OR “Guinea” OR “Guinea-Bissau” OR “Liberia” OR “Mali” OR “Niger” OR “Nigeria” OR “Senegal” OR “Sierra Leone” OR “Togo”)). French equivalents were also included to ensure comprehensive coverage of the literature, such as: (“composition chimique” OR “composition des aliments”) AND (“ruminant” OR “bovin” OR “ovin” OR “caprin”) AND (“sous-produits agro-industriels” OR “sous-produits agricoles” OR “fourrages”). The Boolean search syntax was adapted to each database. In Scopus and Web of Science, Boolean operators (AND, OR) were explicitly applied to structure the search queries. Because Google Scholar handles Boolean logic differently, keyword combinations were used without strict Boolean nesting and the first 200 results ranked by relevance were screened. To ensure consistency and reliability of the dataset, the review focused exclusively on peer-reviewed journal articles. Gray literature sources such as theses, institutional reports and technical documents were not included in the final dataset.

### 2.2. Eligibility Criteria and Study Selection

The selection of studies followed the PRISMA screening procedure. The initial database search identified 2304 records. After removing duplicate records and applying preliminary filters related to publication type, language and geographic scope, 1034 records remained for title and abstract screening. Studies were included if they met the following criteria: conducted in West Africa, published in peer-reviewed journals, focused on feed resources used in ruminant feeding systems, reported quantitative chemical composition data. Studies were excluded if they were review articles without original data, were conference abstracts or methodological papers without analytical data, were conducted outside West Africa, reported only qualitative descriptions without numerical values.

The screening process was conducted independently by two reviewers. Disagreements regarding study inclusion were resolved through discussion until consensus was reached.

**Figure 1:**
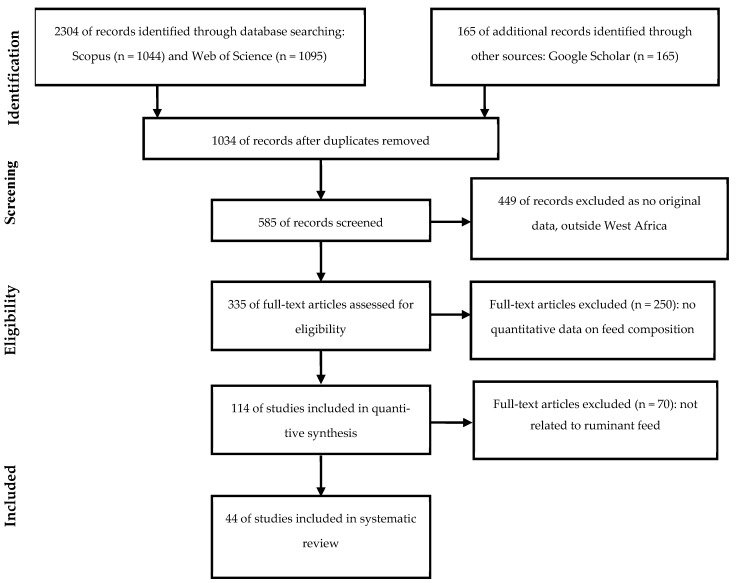
PRISMA flow diagram illustrating the identification, screening, eligibility and inclusion of studies used for the systematic synthesis of feed composition data in West Africa.

Following title and abstract screening, potentially eligible studies were subjected to full-text evaluation. After applying all inclusion and exclusion criteria, 44 articles were retained for data extraction and analysis. The overall study selection process is summarized in a PRISMA flow diagram (Figure 1). The full database-specific search strings are provided in Supplementary Materials Table S1 to improve transparency and reproducibility. The list of selected studies, including authors, titles, year, source title, doi, country, is provided Supplementary Materials Table S2.

### 2.3. Data Extraction and Feed Classification

For each selected study, the following information was extracted: feed resource name, botanical or industrial origin, country of study, analytical methods used, reported values of nutritional parameters. Feed resources were classified into three main categories according to their production origin and their role in livestock feeding systems:

- Agro-industrial by-products, including residues generated from industrial processing of agricultural products such as oilseed cakes, cereal brans, brewers’ grains and fruit pulps.
- Agricultural by-products, including residues produced directly from crop production or harvesting activities such as straws, husks, stalks, pods and crop residues.
- Forages, including natural pastures, cultivated grasses, forage legumes and browse species.

The detailed inventory of feed resources included in each category is presented in Supplementary Materials Tables S3–S5. For instance, agro-industrial by-products include feedstuffs such as cottonseed cake, rice bran and cassava peels, whose chemical composition values were compiled from multiple studies. When feed resources consisted of mixtures of different ingredients or when their classification was ambiguous, the corresponding samples were excluded from the dataset to avoid misclassification. Feed classification and data extraction were initially performed by the first author and subsequently verified by the co-authors to ensure accuracy and consistency. Because analytical methods varied across studies (e.g., crude protein determined by the Kjeldahl or Dumas method, or NDF determined with or without heat-stable amylase), these methodological differences were recorded when available and considered as potential sources of variability in the compiled dataset. These methodological differences were recorded as potential sources of variability, but they were not quantitatively modelled or tested through subgroup analysis in the present synthesis. Whenever necessary, reported values were converted to a dry matter basis to ensure comparability across studies.

### 2.4. Nutritional Parameters Considered

The following nutritional parameters were collected whenever reported: Dry matter (DM), Crude protein (CP), Ether extract (EE), Organic matter (OM), Crude fibre (CF), Neutral detergent fibre (NDF), Acid detergent fibre (ADF), Ash, Digestibility of organic matter (dOM). Energy parameters included: Gross energy (GE), Digestible energy (DE) and Metabolizable energy (ME). Additional feeding system indicators were also recorded: Digestible crude protein (DCP), UFL (Feed Unit for Milk; Unité Fourragère Lait), UFV (Feed Unit for Meat; Unité Fourragère Viande). Energy values were expressed on a dry matter basis (MJ/kg DM) when available. When necessary, values reported in kcal/kg were converted to MJ/kg DM to standardize units across studies. Some parameters such as UFL and UFV were not consistently reported in the West African literature. When available, these values were extracted directly from the original studies. The dataset was based exclusively on values reported in the retained studies. When necessary, values reported in different units were converted to a common dry matter basis to standardize the dataset and improve comparability across studies.

### 2.5. Data Synthesis and Statistical Processing

For each feed resource with data available from at least two independent studies, reported values were compiled into a unified dataset. When multiple values for the same feed resource were reported within a single study (e.g., different sampling locations, seasons or processing conditions), each value was considered as an independent observation. Descriptive statistics were calculated to summarize the variability of feed composition across studies, including: mean, minimum, maximum, standard deviation (SD) and coefficient of variation (CV). Extreme values were visually inspected using boxplot analysis. Outlier identification was based on visual inspection of boxplots and on verification of whether extreme observations were likely to reflect clear analytical or data-entry errors; no formal statistical threshold or influence diagnostics were applied. Values identified as clear analytical errors were excluded from the dataset to improve reliability. All calculations were performed using Microsoft Excel and R software version 4.5.2 [21]. Because feed composition can vary considerably across agro-ecological zones, plant maturity stages, feed processing methods and analytical techniques, the reported ranges reflect the natural variability of feed resources across West African production environments, rather than single reference values.

## 3. Results

### 3.1. Temporal Trend in Studies on Ruminant Feed Resources in West Africa

**Figure 2:**
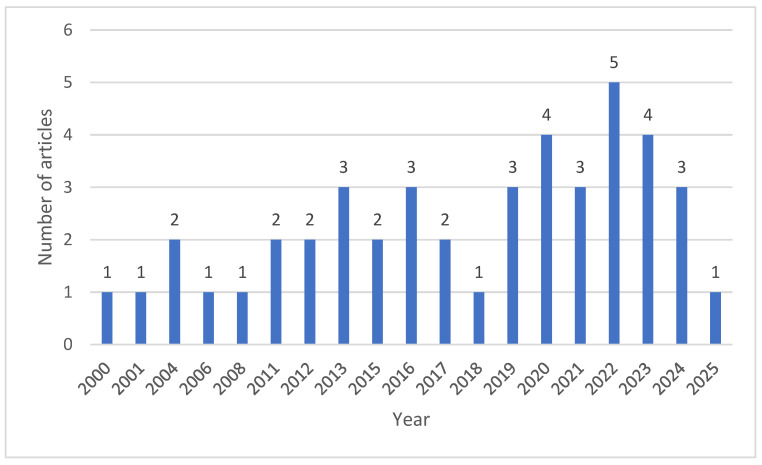
Temporal distribution of the studies included in the database of ruminant feed resources in West Africa (2000–2025).

The number of eligible studies was low and irregular during the early 2000s, with only isolated publications in several years (Figure 2). From 2011 onwards, the literature became more continuous, and a clearer increase was observed after 2018. The highest publication output was recorded in 2022, followed by 2020, 2023 and 2024, indicating growing research interest in the characterization of ruminant feed resources in West Africa during recent years. This pattern also suggests that the evidence base used in the present synthesis is relatively recent, although temporal gaps remain for some periods.

### 3.2. Geographical Distribution of Studies on Ruminant Feed Resources in West Africa

**Figure 3:**
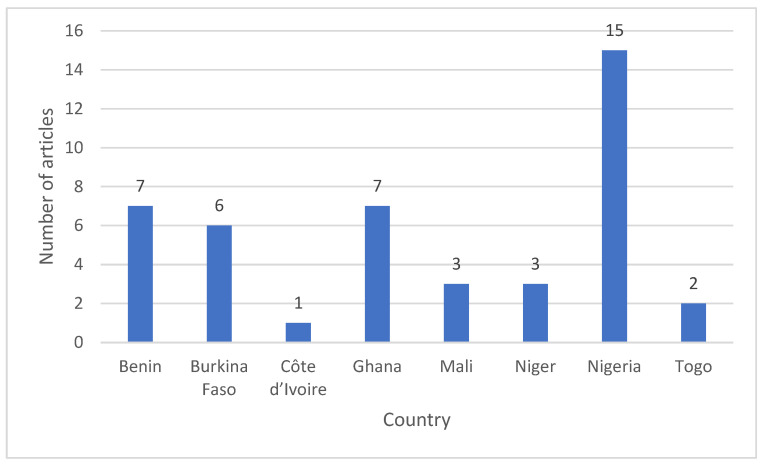
Geographic distribution of the studies included in the database of ruminant feed resources across West African countries.

The retained studies were unevenly distributed across countries. Nigeria contributed the largest number of articles, followed by Benin and Ghana, whereas Burkina Faso also showed substantial representation. In contrast, Mali, Niger and Togo were represented by fewer studies, and Côte d’Ivoire had only limited coverage (Figure 3). This spatial imbalance indicates that the regional evidence base is dominated by a few countries, which should be considered when interpreting the overall averages reported in the feed resource tables. It also highlights the need for additional analytical studies in underrepresented West African countries to improve regional representativeness.

### 3.3. Chemical Composition of Agro-Industrial By-Products

Agro-industrial by-products showed marked heterogeneity in chemical composition across West Africa (Table 1). The complete list of feed materials, together with their mean, minimum, maximum and standard deviation values, is provided in Supplementary Materials Table S3. Among the retained ingredients, cottonseed cake had the highest crude protein concentration (32.85 ± 10.31% DM) and digestible crude protein content (301.30 ± 108.66 g/kg DM), confirming its value as a major protein supplement for ruminants. Cotton seeds also had a relatively high crude protein concentration (22.00 ± 3.50% DM) but were distinguished by the highest ether extract content (20.80 ± 0.71% DM), indicating a dual contribution of protein and lipid. In contrast, cassava peels had a much lower crude protein concentration (5.34 ± 0.09% DM) and a relatively high fibre content (NDF: 54.18 ± 6.53% DM), suggesting that they function mainly as low-protein energy by-products requiring nitrogen supplementation. Wheat bran (15.94 ± 2.12% DM CP) and maize bran (11.44 ± 4.40% DM CP) showed intermediate nutritional profiles. Standard deviations indicated substantial between-study variation for several ingredients, particularly cottonseed cake (e.g., EE: 14.97 ± 11.47% DM; NDF: 40.97 ± 19.49% DM) and maize bran (ADF: 24.70 ± 32.84% DM), whereas variability was lower for cassava peels for crude protein (5.34 ± 0.09% DM). Overall, these results indicate that agro-industrial by-products should not be treated as a nutritionally uniform class, and that both ingredient type and compositional variability should be considered when using tabulated values for ration formulation in West African ruminant systems.

### 3.4. Chemical Composition of Agricultural By-Products

Table 2 showed marked heterogeneity in the composition of agricultural by-products used in ruminant feeding across West Africa. Legume-derived residues were generally richer in crude protein than cereal-derived residues, with peanut haulms presenting the highest crude protein concentration (15.69 ± 4.96% DM), followed by groundnut haulms (12.30 ± 2.62% DM) and cowpea haulms (11.76 ± 3.96% DM). In contrast, cereal residues such as rice straw (5.37 ± 1.14% DM), sorghum stover (4.82 ± 2.27% DM), and sorghum straw (4.14 ± 2.95% DM) were consistently poorer in protein, while millet stover showed an intermediate value (8.37 ± 5.67% DM). Fibre concentrations were highest in cereal residues, particularly in sorghum stover (NDF: 82.59 ± 12.72% DM; ADF: 69.04 ± 29.47% DM), millet stover (NDF: 75.00 ± 15.41% DM; ADF: 62.15 ± 12.66% DM), and sorghum straw (NDF: 73.94 ± 3.55% DM; ADF: 52.28 ± 9.92% DM), confirming their highly fibrous nature. By comparison, legume haulms had lower NDF values, including 47.26 ± 21.08% DM for peanut haulms and 54.71 ± 11.98% DM for groundnut haulms, indicating a comparatively better feeding value. Digestibility-related traits followed the same pattern, with groundnut haulms showing the highest digestible organic matter (72.00 ± 0.00%) and peanut haulms also showing relatively high values (61.47 ± 5.13%), whereas millet stover had the lowest dOM (35.00 ± 0.00%). The large standard deviations observed for several ingredients, especially for peanut haulms (e.g., NDF: 47.26 ± 21.08% DM) and sorghum stover (ADF: 69.04 ± 29.47% DM), indicate substantial between-study variation. Overall, these results show that agricultural by-products in West Africa can be broadly separated into protein-richer legume haulms and low-protein, highly fibrous cereal residues, and that their use in ration formulation should account for both ingredient class and compositional variability. The full list of agricultural by-products retained in the database, together with their mean, minimum, maximum standard deviation and coefficient of variation values, is presented in Supplementary Materials Table S4.

### 3.5. Chemical Composition of Forages

Table 3 showed marked heterogeneity in the composition of forage resources used in ruminant feeding across West Africa. Browse legumes and tree leaves were generally richer in crude protein than grasses, with Moringa oleifera showing the highest crude protein concentration (32.90 ± 7.75% DM), followed by Manihot esculenta (26.87 ± 2.58% DM), Leucaena leucocephala (25.84 ± 4.78% DM), and Gliricidia sepium (20.71 ± 4.89% DM). In contrast, grasses such as Panicum maximum contained much lower crude protein (9.19 ± 2.85% DM) and higher fibre concentrations (NDF: 73.64 ± 5.32% DM), confirming their role as basal roughages rather than protein supplements. Intermediate values were observed for browse species such as Afzelia africana (18.39 ± 5.68% DM CP) and Pterocarpus erinaceus (15.56 ± 3.46% DM CP). Fibre fractions also differed substantially across forage types, with relatively high NDF values in Piliostigma thonningii (59.82 ± 1.24% DM), Afzelia africana (54.67 ± 24.93% DM), and Pterocarpus erinaceus (52.98 ± 1.56% DM), whereas Moringa oleifera and Manihot esculenta showed comparatively lower fibre concentrations. Digestibility-related traits followed the same general pattern, with Moringa oleifera showing the highest digestible organic matter (61.50 ± 13.07%) among the major retained forages, while Piliostigma thonningii had the lowest (36.00 ± 0.00%). The relatively large standard deviations recorded for several ingredients, especially for Panicum maximum (DM: 66.38 ± 26.40%) and Pterocarpus erinaceus (DM: 61.52 ± 33.76%), indicate substantial between-study variation, likely reflecting differences in site conditions, plant maturity, season, and analytical procedures. Overall, these results confirm that forage resources in West Africa should not be considered nutritionally uniform and that both forage subclass and compositional variability should be taken into account in ration formulation. The complete list of retained forage resources, together with their mean, minimum, maximum and standard deviation values, is provided in Supplementary Materials Table S5.

## 4. Discussion

### 4.1. Main Patterns Emerging from the Evidence on Ruminant Feed Resources in West Africa

The present review highlights substantial nutritional heterogeneity among ruminant feed resources used in West Africa. This variability appears to reflect the combined effects of agro-ecological conditions, plant maturity, post-harvest handling, and processing methods. For example, the large variation observed in dry matter for Panicum maximum and Pterocarpus erinaceus is consistent with differences in harvest stage, regrowth age, and seasonal conditions, as also suggested by [41,43,51]. Likewise, the marked dispersion in crude protein, ether extract, and fibre fractions of cottonseed cake is coherent with differences in oil extraction efficiency, hull inclusion, and local processing practices, as indicated by [23,26]. Thus, the observed variability is likely to reflect real biological and technological differences across feed resources, although some methodological contribution cannot be ruled out.

This nutritional variability is of direct practical importance for ration formulation. In smallholder systems where basal diets are often based on low-quality cereal residues, even moderate variation in supplement composition may affect rumen nitrogen supply and animal performance. This is particularly relevant for residues such as rice straw, sorghum straw, and millet stover, which showed consistently low crude protein concentrations, compared with legume haulms and several browse resources. The meta-analysis of [52] showed that supplementation of low-quality roughages with tree foliage significantly affects digestibility, nitrogen utilization, and ruminal fermentation, supporting the view that relatively small fluctuations in feed composition may have meaningful nutritional consequences. In this context, the use of reference ranges rather than single-point values appears more appropriate, especially for ingredients showing high dispersion, such as cottonseed cake, maize bran, peanut haulms, sorghum stover, and several woody forage species.

The comparison among feed classes also reveals clear nutritional contrasts with important implications for feeding strategies. Among agro-industrial by-products, cottonseed cake emerged as a major protein-rich supplement, while cotton seeds combined both protein and lipid supply. In contrast, cassava peels behaved mainly as low-protein energy by-products, and wheat bran and maize bran occupied an intermediate position. These findings are consistent with reports from [22,24,26], they confirm that agro-industrial by-products should not be treated as a nutritionally uniform group.

Agricultural by-products showed a similarly strong contrast between legume haulms and cereal residues. Groundnut haulms, peanut haulms, and cowpea haulms consistently contained more crude protein than rice straw, sorghum straw, and millet stover, confirming their importance as strategic dry-season supplements. This pattern agrees with findings by [31] in Niger, [53] in Ghana, and [12] in Nigeria. By contrast, cereal residues were characterized by higher NDF and ADF concentrations, as also reported by [27,34,54]), indicating more severe structural limitations to intake and digestibility. These results support the classical complementarity between low-protein basal roughages and protein-rich supplements in West African mixed crop–livestock systems.

The forage dataset also confirmed strong subclass differences between grasses, browse species, and tree legumes. Protein-rich resources such as *Moringa oleifera*, *Leucaena leucocephala*, *Manihot esculenta*, and *Gliricidia sepium* clearly contrasted with basal grasses such as Panicum maximum, reinforcing the importance of woody and non-conventional feed resources in West African systems. This is consistent with studies from Benin, Ghana, and Nigeria by [13,25,43,47]. At the same time, browse species such as *Afzelia africana*, *Piliostigma thonningii*, and *Pterocarpus erinaceus* showed intermediate profiles, suggesting an important role as supplementary feeds, particularly under seasonal feed shortages.

The West African evidence base also appears regionally distinctive when compared with findings from other African regions. Studies from East Africa, such as those discussed by [55], likewise reported considerable agro-ecological variation in feed quality. However, the current synthesis suggests that West African systems are particularly characterized by the importance of browse species, tree leaves, legume haulms, and non-conventional feedstuffs in filling dry-season nutrient gaps. This regional specificity supports the value of developing West Africa-specific reference tables rather than relying solely on generalized international feed databases.

The present review also strengthens the argument that non-traditional feed resources can play a strategic role in ruminant feeding. Several woody species retained in this synthesis had relatively high crude protein concentrations, but their practical value should not be assessed on crude protein alone. Secondary metabolites such as tannins and saponins may improve protein utilization at moderate concentrations but may also depress intake or digestibility at excessive levels. This is particularly relevant for browse-based feeding systems. Recent meta-analytic evidence in small ruminants confirms that dietary tannins can influence growth performance, serum metabolites, ruminal fermentation, and product quality depending on dose and feeding context [56]. Therefore, the potential of woody resources in West Africa should be interpreted in light of both their protein value and their possible anti-nutritional constraints.

From an applied perspective, these results indicate that feed resources in West Africa should be characterized not only by origin, but also by nutritional function. In practice, the values reported here can support more robust feed planning under fluctuating quality conditions. For example, combinations such as sorghum straw and cottonseed cake, stover and legume haulms, or *Panicum maximum* and *Leucaena leucocephala* may be better formulated using minimum, mean, and maximum reference values rather than a single value per ingredient. This has direct implications for decision-support systems and digital feeding tools, which should ideally incorporate uncertainty margins and not only fixed composition values.

### 4.2. Limitations of the Evidence Base and Implications for Interpretation

Several limitations should be considered when interpreting the present synthesis. First, the evidence base was geographically uneven, with a strong concentration of studies from Nigeria, Benin, Ghana, and Burkina Faso, while other West African countries were much less represented. As a result, the regional averages reported here may disproportionately reflect the ecological conditions, feed resources, and analytical traditions of the most studied countries.

Second, some ingredients were represented by relatively few studies, and not all nutritional parameters were reported consistently across sources. This explains the presence of missing values for several feed materials and limits direct comparison across all variables. In addition, part of the variability observed across studies may still reflect differences in analytical procedures, sample preservation, laboratory methods, and calculation approaches. Therefore, although much of the variation is likely to be biologically and technologically meaningful, it cannot be claimed that it is entirely independent of methodological effects. This should therefore be considered a limitation of the review. In addition, outlier assessment was based on visual inspection rather than on formal statistical influence diagnostics, which should also be considered a limitation of the review.

Third, the present database describes the nutritional characteristics of individual feed ingredients, whereas smallholder farmers usually feed animals with combinations of resources rather than isolated ingredients. In practice, West African feeding systems commonly involve mixtures such as straw plus oilseed cake, cereal stover plus haulms, or grasses plus browse leaves. The values reported here therefore represent the composition of individual feedstuffs and should not be interpreted as the nutritive value of complete diets. Associative effects, substitution responses, and feed interactions may alter actual animal responses under field conditions.

Fourth, the review did not explicitly incorporate actual feeding practices, economic access, seasonal availability, or the competing uses of feed resources at farm level. Some feedstuffs with good nutritional potential may be scarce, expensive, or reserved for other purposes, which may restrict their practical use by smallholders. Likewise, potential risks related to storage quality, mould contamination, mycotoxins, or anti-nutritional compounds were not systematically quantified across the retained literature.

Taken together, these limitations suggest that the current database should be interpreted as a reference framework for ingredient-level feed evaluation, rather than as a direct prescription for field ration formulation. Future work should therefore strengthen geographical coverage, improve methodological harmonization, and more explicitly connect feed composition data with real feeding combinations and decision contexts in West African smallholder livestock systems.

## 5. Conclusions

This review establishes region-specific reference ranges for the main ruminant feed resources used in West Africa and demonstrates that these ranges are more informative than single-point values for feed evaluation and ration formulation. Its main practical contribution is to provide a more reliable basis for matching supplements to basal diets, thereby helping to reduce formulation errors, limit nutritional imbalances, decrease inefficient nutrient use, and ultimately minimize avoidable production losses in smallholder systems. The synthesis also highlights clear nutritional contrasts among feed classes: cotton-derived by-products were the most important protein-rich agro-industrial supplements, legume haulms consistently showed higher nutritional value than cereal residues among agricultural by-products, and several browse and tree species, particularly *Moringa oleifera*, *Leucaena leucocephala*, *Gliricidia sepium*, and *Manihot esculenta*, emerged as valuable protein-rich forage resources for dry-season feeding. At the same time, the results confirm that feed composition in West Africa is strongly influenced by local conditions, maturity stage, and processing methods, which means that the reported values should be used as ingredient-level reference ranges rather than as fixed ration standards. Future research should therefore prioritize multi-site characterization of key West African feedstuffs using harmonized analytical methods, especially for widely used but still under-documented local resources and should translate these data into simple locally adapted ration formulation tools, such as practical calculators based on common feed combinations used by farmers.

## Figures and Tables

**Table 1:** Average chemical composition of selected agro-industrial by-products for ruminants in West Africa.

Feed	DM	EE	OM	dOM	CP	CF	Ash	GE	DE	ME	DCP	UFL	UFV	NDF	ADF	Ca	P	Representative References
Cassava peels	91.28 ± 2.07	2.96 ± 1.64	94.69 ± 0.02	68.44 ± 0.00	5.34 ± 0.09	13.40 ± 4.86	6.56 ± 2.06	16.52 ± 3.41	–	–	–	0.93 ± 0.00	0.89 ± 0.00	54.18 ± 6.53	32.03 ± 15.15	0.45 ± 0.00	0.80 ± 0.00	[22,23,24] (n = 3)
Cotton seeds	90.80 ± 0.99	20.80 ± 0.71	95.75 ± 0.64	56.00 ± 0.00	22.00 ± 3.50	27.80 ± 3.54	4.25 ± 0.64	–	–	–	150.33 ± 7.64	1.05 ± 0.19	–	46.60 ± 0.00	29.80 ± 0.00	0.17 ± 0.00	0.76 ± 0.00	[25,26] (n = 2)
Cottonseed cake	91.38 ± 4.15	14.97 ± 11.47	91.63 ± 2.05	74.00 ± 0.00	32.85 ± 10.31	26.94 ± 13.23	6.36 ± 1.22	7.80 ± 0.00	–	–	301.30 ± 108.66	0.85 ± 0.27	0.82 ± 0.00	40.97 ± 19.49	30.27 ± 18.33	0.28 ± 0.00	1.21 ± 0.00	[23,26,27,28,29] (n = 5)
Maize bran	92.80 ± 0.22	1.60 ± 0.00	91.80 ± 5.09	88.00 ± 0.00	11.44 ± 4.40	10.83 ± 5.08	4.38 ± 1.15	–	–	–	68.55 ± 51.04	1.32 ± 0.00	–	41.38 ± 21.62	24.70 ± 32.84	–	–	[23,30] (n = 2)
Wheat bran	93.75 ± 2.67	3.81 ± 0.58	94.40 ± 0.44	79.00 ± 0.00	15.94 ± 2.12	11.61 ± 4.59	7.72 ± 3.45	12.97 ± 0.00	–	–	125.00 ± 0.00	–	–	47.55 ± 12.22	24.89 ± 15.56	1.07 ± 0.00	0.97 ± 0.00	[22,27,31,32] (n = 4)

DM = dry matter (%); EE = ether extract (% DM); OM = organic matter (% DM); dOM = digestible organic matter (%); CP = crude protein (% DM); CF = crude fibre (% DM); Ash = ash (% DM); GE = gross energy (MJ/kg DM); DE = digestible energy (MJ/kg DM); ME = metabolizable energy (MJ/kg DM); DCP = digestible crude protein (g/kg DM); UFL = feed unit for milk; UFV = feed unit for meat; NDF = neutral detergent fibre (% DM); ADF = acid detergent fibre (% DM); Ca = calcium (% DM); P = phosphorus (% DM). Values are presented as mean ± SD.

**Table 2:** Average chemical composition of selected agricultural by-products for ruminants in West Africa.

Feed	DM	EE	OM	dOM	CP	CF	Ash	GE	DE	ME	DCP	UFL	UFV	NDF	ADF	Ca	P	Representative References
Cowpea haulms	93.56 ± 2.25	5.24 ± 4.37	85.74 ± 2.36	55.50 ± 0.00	11.76 ± 3.96	28.82 ± 11.04	9.33 ± 2.40	–	–	7.68 ± 0.00	5.38 ± 4.56	–	0.41 ± 0.00	57.41 ± 12.74	49.42 ± 17.98	–	–	[12,23,26,27,30,33] (n = 6)
Groundnut haulms	88.94 ± 4.14	10.45 ± 0.00	91.44 ± 1.79	72.00 ± 0.00	12.30 ± 2.62	29.80 ± 10.90	8.89 ± 2.02	–	–	–	50.38 ± 63.10	0.76 ± 0.00	–	54.71 ± 11.98	39.97 ± 5.04	0.80 ± 0.09	0.10 ± 0.04	[23,31,34] (n = 3)
Millet stover	89.40 ± 6.22	–	93.50 ± 0.00	35.00 ± 0.00	8.37 ± 5.67	41.40 ± 0.00	7.50 ± 0.00	–	–	5.24 ± 0.00	1.90 ± 0.00	–	–	75.00 ± 15.41	62.15 ± 12.66	0.20 ± 0.00	0.04 ± 0.00	[26,31,33] (n = 3)
Peanut haulms	88.05 ± 11.30	4.37 ± 5.32	85.95 ± 12.10	61.47 ± 5.13	15.69 ± 4.96	28.77 ± 8.73	9.90 ± 2.45	18.35 ± 2.83	–	4.97 ± 0.00	13.26 ± 3.31	0.73 ± 0.00	0.52 ± 0.14	47.26 ± 21.08	41.59 ± 7.33	1.10 ± 0.00	1.50 ± 0.00	[23,26,27,28,31,33] (n = 6)
Rice straw	93.33 ± 0.35	–	83.05 ± 0.50	55.00 ± 0.00	5.37 ± 1.14	38.00 ± 0.00	15.60 ± 2.37	–	–	–	12.00 ± 0.00	–	–	62.83 ± 4.61	51.70 ± 0.00	–	–	[26,35,36] (n = 3)
Sorghum stover	91.84 ± 5.97	1.07 ± 0.62	84.84 ± 12.51	46.70 ± 0.00	4.82 ± 2.27	38.79 ± 6.29	13.75 ± 9.11	–	–	6.90 ± 0.00	1.40 ± 0.00	–	–	82.59 ± 12.72	69.04 ± 29.47	–	–	[23,26,32,33] (n = 4)
Sorghum straw	93.71 ± 3.30	1.52 ± 0.00	88.59 ± 5.06	46.00 ± 0.00	4.14 ± 2.95	41.78 ± 3.92	7.72 ± 1.73	–	–	–	2.05 ± 2.76	0.60 ± 0.00	0.30 ± 0.00	73.94 ± 3.55	52.28 ± 9.92	0.31 ± 0.00	0.70 ± 0.00	[27,34], (n = 2)

DM = dry matter (%); EE = ether extract (% DM); OM = organic matter (% DM); dOM = digestible organic matter (%); CP = crude protein (% DM); CF = crude fibre (% DM); Ash = ash (% DM); GE = gross energy (MJ/kg DM); DE = digestible energy (MJ/kg DM); ME = metabolizable energy (MJ/kg DM); DCP = digestible crude protein (g/kg DM); UFL = feed unit for milk; UFV = feed unit for meat; NDF = neutral detergent fibre (% DM); ADF = acid detergent fibre (% DM); Ca = calcium (% DM); P = phosphorus (% DM). Values are presented as mean ± SD.

**Table 3:** Average chemical composition of selected forages for ruminants in West Africa.

Feed	DM	EE	OM	dOM	CP	CF	Ash	GE	DE	ME	DCP	UFL	UFV	NDF	ADF	Ca	P	Representative References
*Afzelia africana*	39.70 ± 1.41	6.38 ± 0.00	89.30 ± 5.72	43.00 ± 0.00	18.39 ± 5.68	38.60 ± 0.00	13.54 ± 4.08	–	–	–	121.00 ± 0.00	0.61 ± 0.05	0.47 ± 0.00	54.67 ± 24.93	47.08 ± 32.00	0.77 ± 0.00	0.67 ± 0.00	[37,38,39] (n = 3)
*Commelina benghalensis*	81.07 ± 18.65	1.70 ± 0.99	86.01 ± 7.34	–	11.18 ± 7.25	9.00 ± 0.00	10.83 ± 7.55	–	–	9.00 ± 0.00	–	–	–	52.24 ± 0.00	31.54 ± 0.00	0.50 ± 0.00	3.83 ± 0.00	[40,41,42] (n = 3)
*Elaeis guineensis*	90.06 ± 0.37	2.85 ± 1.31	90.28 ± 2.03	56.01 ± 1.92	13.62 ± 2.74	22.89 ± 0.00	9.71 ± 2.03	–	–	10.84 ± 0.68	–	–	–	52.59 ± 1.86	34.09 ± 2.49	–	–	[6,43,44] (n = 3)
*Gliricidia sepium*	71.06 ± 34.56	3.48 ± 2.41	90.06 ± 2.08	57.50 ± 0.00	20.71 ± 4.89	13.10 ± 4.03	10.07 ± 1.83	14.89 ± 3.25	17.80 ± 0.00	–	–	–	–	47.35 ± 15.20	30.56 ± 9.37	1.38 ± 0.00	0.31 ± 0.00	[22,24,25,43,45] (n = 5)
*Leucaena leucocephala*	65.17 ± 30.10	3.47 ± 0.47	92.75 ± 2.68	41.96 ± 0.00	25.84 ± 4.78	11.06 ± 3.40	7.29 ± 2.45	19.74 ± 2.34	–	–	19.30 ± 0.00	–	–	46.89 ± 11.43	29.89 ± 8.02	–	–	[13,24,25,35,43,45,46] (n = 7)
*Mangifera indica*	92.35 ± 2.48	3.29 ± 0.51	91.41 ± 3.85	42.76 ± 1.08	10.02 ± 1.29	21.96 ± 3.74	8.69 ± 3.14	–	–	–	–	–	–	48.08 ± 4.59	37.53 ± 1.10	–	–	[43,45,47] (n = 3)
*Manihot esculenta*	95.61 ± 0.00	9.31 ± 4.86	92.15 ± 0.02	49.22 ± 4.39	26.87 ± 2.58	8.87 ± 0.00	7.89 ± 0.09	–	–	–	–	–	–	42.02 ± 11.43	32.29 ± 10.28	–	–	[6,43,45] (n = 3)
*Moringa oleifera*	90.53 ± 3.58	5.90 ± 3.01	86.91 ± 2.65	61.50 ± 13.07	32.90 ± 7.75	10.57 ± 4.28	11.68 ± 3.56	–	–	–	–	–	–	37.69 ± 7.57	26.85 ± 8.01	–	–	[43,45,47] (n = 3)
*Panicum maximum*	66.38 ± 26.40	1.89 ± 0.51	90.08 ± 1.64	58.63 ± 0.00	9.19 ± 2.85	24.00 ± 9.45	9.94 ± 1.62	14.81 ± 2.31	–	13.13 ± 0.00	–	0.09 ± 0.00	0.11 ± 0.00	73.64 ± 5.32	41.36 ± 0.00	0.05 ± 0.00	0.03 ± 0.00	[22,25,41,48,49,50,51] (n = 7)
*Piliostigma thonningii*	76.07 ± 25.98	4.65 ± 1.07	89.87 ± 9.94	36.00 ± 0.00	12.72 ± 2.86	22.88 ± 5.26	11.05 ± 8.64	–	–	–	64.00 ± 0.00	0.46 ± 0.00	0.34 ± 0.00	59.82 ± 1.24	41.69 ± 25.89	0.82 ± 0.00	0.38 ± 0.00	[38,39,45] (n = 3)
*Pterocarpus erinaceus*	61.52 ± 33.76	5.23 ± 1.39	90.72 ± 3.77	44.43 ± 1.80	15.56 ± 3.46	28.30 ± 0.00	9.33 ± 2.79	–	–	14.52 ± 0.00	148.00 ± 0.00	0.56 ± 0.05	0.49 ± 0.00	52.98 ± 1.56	40.45 ± 8.23	0.44 ± 0.00	0.83 ± 0.00	[37,38,39,40,41,43] (n = 6)

DM = dry matter (%); EE = ether extract (% DM); OM = organic matter (% DM); dOM = digestible organic matter (%); CP = crude protein (% DM); CF = crude fibre (% DM); Ash = ash (% DM); GE = gross energy (MJ/kg DM); DE = digestible energy (MJ/kg DM); ME = metabolizable energy (MJ/kg DM); DCP = digestible crude protein (g/kg DM); UFL = feed unit for milk; UFV = feed unit for meat; NDF = neutral detergent fibre (% DM); ADF = acid detergent fibre (% DM); Ca = calcium (% DM); P = phosphorus (% DM). Values are presented as mean ± SD.

## Data Availability

The original contributions presented in this study are included in the article. Further inquiries can be directed to the corresponding author.

## Supplementary Materials

References [6,12,13,22,23,24,25,26,27,28,29,30,31,32,33,34,35,36,37,38,39,40,41,42,43,44,45,46,47,48,49,50,51,53,54,57,58,59,60,61,62,63,64,65] are cited in the Supplementary Materials. The following supporting information can be downloaded at: https://www.mdpi.com/article/10.3390/ani16081215/s1, Table S1: Full search strings used in Scopus, Web of Science, and Google Scholar; Table S2: List of studies included in the systematic review and used for feed composition data extraction in West Africa; Table S3: Chemical composition of agro-industrial by-products for ruminants in West-Africa; Table S4. Table of chemical composition of agricultural by-products for ruminants in West-Africa; Table S5. Table of chemical composition of forages for ruminants in West-Africa.

## References

[1] FAO (2011). World Livestock 2011 Livestock in Food Security.

[2] Carlo A., Derek B., Cheikh L., Nancy Ruth M., Simplice N., Longin N., Patrick O., Ugo P.-C., Joseph S., Alberto Z. Investing in the Livestock Sector: Why Good Numbers Matter—A Sourcebook for Decision Makers on How to Improve Livestock Data. https://documents.worldbank.org/en/publication/documents-reports/documentdetail/850001468149370813.

[3] Assani A.S., Yarou A.K., Dedehou N.V.F.G., Worogo H.S., Baco M.N., Houinato M., Alkoiret I.T. (2024). Towards Indigenous Community-Based Adaptation to Climate Change: A Typological Analysis of Tree-Livestock Integration in Smallholding Systems in Dryland Areas of Benin (West-Africa). Agroforest Syst..

[4] Alimi N., Assani A.S., Sanni Worogo H., Baco N.M., Traoré I.A. (2024). Livestock Feed Resources Used as Alternatives during Feed Shortages and Their Impact on the Environment and Ruminant Performance in West Africa: A Systematic Review. Front. Vet. Sci..

[5] Ayantunde A.A., Amole T.A. (2016). Improving Livestock Productivity: Assessment of Feed Resources and Livestock Management Practices in Sudan-Savanna Zones of West Africa. AJAR.

[6] Koura B.I., Vastolo A., Kiatti D.D., Cutrignelli M.I., Houinato M., Calabrò S. (2022). Nutritional Value of Climate-Resilient Forage Species Sustaining Peri-Urban Dairy Cow Production in the Coastal Grasslands of Benin (West Africa). Animals.

[7] NRC (2001). Nutrient Requirements of Dairy Cattle: Seventh Revised Edition.

[8] INRA (2018). INRA Feeding System for Ruminants.

[9] Brah N., Houndonougbo F.M., Issa S., Chrysostome A.A.M. (2019). Tableur Ouest Africain de Formulation d’Aliments de Volailles (TOAFA–Volaille). Int. J. Biol. Chem. Sci..

[10] Despal D., Yulianti Y.I., Zahera R., Agustiyani I., Rosmalia A., Afnan I.M., Zain M., Tanuwiria U.H. (2023). Comparison of Chemical Composition, In Vitro Digestibility, and Near Infrared Reflectance Spectroscopy in Estimating In Situ Rumen Degradable Protein of Tropical Foliage. Trop. Anim. Sci. J..

[11] Rahimi J., Haas E., Grote R., Kraus D., Smerald A., Laux P., Goopy J., Butterbach-Bahl K. (2021). Beyond Livestock Carrying Capacity in the Sahelian and Sudanian Zones of West Africa. Sci. Rep..

[12] Anele U.Y., Südekum K.-H., Hummel J., Arigbede O.M., Oni A.O., Olanite J.A., Böttger C., Ojo V.O., Jolaosho A.O. (2012). Chemical Characterization, in Vitro Dry Matter and Ruminal Crude Protein Degradability and Microbial Protein Synthesis of Some Cowpea (*Vigna unguiculata* L. Walp) Haulm Varieties. Anim. Feed Sci. Technol..

[13] Anyanwu N.J., Etela I. (2013). Chemical Composition and Dry Matter Degradation Characteristics of Multi-Purpose Trees and Shrubs in the Humid Lowlands of Southeastern Nigeria. Agroforest Syst..

[14] Van Soest P.J. (1994). Nutritional Ecology of the Ruminant.

[15] Mertens D.R. (2002). Gravimetric Determination of Amylase-Treated Neutral Detergent Fiber in Feeds with Refluxing in Beakers or Crucibles: Collaborative Study. J. AOAC Int..

[16] Boukrouh S. (2025). Sustainable Sheep Feed: Nutritional Value, Conservation Methods, and Performance Outcomes.

[17] Boukrouh S., Karouach F., El Aayadi S., El Amiri B., Hornick J.-L., Nilahyane A., Hirich A. (2026). A Systematic Review and Meta-Analysis of the Effects of Inclusion of Microalgae in Dairy Cows’ Diets on Nutrient Digestibility, Fermentation Parameters, Blood Metabolites, Milk Production, and Fatty Acid Profiles. Arch. Anim. Breed..

[18] Makkar H.P.S., Tran G., Heuzé V., Giger-Reverdin S., Lessire M., Lebas F., Ankers P. (2016). Seaweeds for Livestock Diets: A Review. Anim. Feed Sci. Technol..

[19] Hughes M.P., Jennings P.G.A., Mlambo V., Lallo C.H.O. Mitigating the Nutritional Limitations to Animal Production from Tropical Pastures: A Review. Proceedings of the 49th Annual Meeting.

[20] Moher D., Liberati A., Tetzlaff J., Altman D.G., Group T.P. (2009). Preferred Reporting Items for Systematic Reviews and Meta-Analyses: The PRISMA Statement. PLoS Med..

[21] R Core Team (2025). R: A Language and Environment for Statistical Computing.

[22] Lamidi A., Ogunkunle T. (2015). Chemical Composition, Mineral Profile and Phytochemical Properties of Common Feed Resources Used for Small Ruminant Animal Production in South-West, Nigeria. Int. J. Des. Nat..

[23] Montcho M., Babatoundé S., Aboh B., Bahini M.J., Chrysostome A.A.M., Mensah G. (2016). Disponibilite, Valeurs Marchande et Nutritionnelle des Sous-Produits Agricoles et Agroindustriels Utilises Dans L’alimentation des Ruminants Au Benin. Eur. Sci. J. ESJ.

[24] Oduguwa B.O., Oni A.O., Arigbede O.M., Adesunbola J.O., Sudekum K.H. (2013). Feeding Potential of Cassava (*Manihot esculenta* Crantz) Peels Ensiled with *Leucaena leucocephala* and *Gliricidia sepium* Assessed with West African Dwarf Goats. Trop. Anim. Health Prod..

[25] Idrissou Y., Assani A.S., Alkoiret Traoré A., Mensah G. (2017). Performances d’embouche Des Ovins Djallonké Complémentés Avec Les Fourrages de *Gliricidia sepium* et de *Leucaena leucocephala* Au Centre Du Bénin. Bull. Rech. Agron. Bénin.

[26] Sanogo O.M., Doumbia S., Descheemaeker K. (2019). Complémentation des bovins laitiers pour l’amélioration de la production de lait et du fumier en milieu paysan dans le cercle de Koutiala. Rev. Malienne Sci. Technol..

[27] Kondombo S.R., Niannongo A.J. (2001). Perfomance d’ovins Djallonké alimentés à base de résidus de récolte au Burkina Faso. Agron. Afr..

[28] Sidibé S., Tangara M., Cissé S.M., Doumbia S., Maïga A.M., Mallé B., Nantoumé H. (2019). Effets de la fane de Cassia tora sur les performances zootechniques des béliers Djallonké en station. Rev. Malienne Sci. Technol..

[29] Dokui F., Houndonougbo F.M., Djidda S.G., Houndonougbo V.P., Gangbedji E., Agbo G.M., Dedome S.L., Babatoundé S., Toleba S.S., Chrysostome C.A. (2022). Milk Yield of Borgou Cows Improved with Lick Stones Made in Benin. AAVS.

[30] Kiema S., Kini L., Ouedraogo S., Kabore/Zoungrana C.Y. (2019). Effet de l’utilisation des gousses de Faidherbia albida sur les performances de croissance des taurillons à l’Ouest du Burkina Faso. Sci. Nat. Appl..

[31] Abdou N., Nsahlai I.V., Chimonyo M. (2011). Effects of Groundnut Haulms Supplementation on Millet Stover Intake, Digestibility and Growth Performance of Lambs. Anim. Feed Sci. Technol..

[32] Abdou Malam M., Gomma Dan A., Issa S., Yahoussa G., Karimou M., Bagnan S., Moussa Y.Z. (2022). Performance Zootechnique Des Jeunes Ovins Mâles Nourris En Complémentation Au Résidu de Moringa (*Moringa oleifera* Lam.) Au Niger: Zootechnical Performance of Young Male Sheep Fed as a Supplement to Moringa Residue (*Moringa oleifera* Lam.) in Niger. Int. J. Biol. Chem. Sci..

[33] Amole T.A., Panyan E., Adekeye A., Ayantunde A., Duncan A., Blummel M. (2022). Productivity Nutritive Value and Economic Potential of Irrigated Fodder in Two Regions of Ghana. Agron. J..

[34] Nantoumé H., Kouriba A., Togola D., Ouologuem B. (2000). Mesure de la valeur alimentaire de fourrages et de sous-produits utilisés dans l’alimentation des petits ruminants. Rev. D’élevage Méd. Vét. Des Pays Trop..

[35] Idan F., Adogla-Bessa T., Sarkwa F.O., Frimpong Y.O., Antwi C. (2023). Effects of Supplementing Rice Straw with Two Fodder Tree Leaves and Their Combinations on Voluntary Feed Intake, Growth, and Nitrogen Utilization in Sheep. Transl. Anim. Sci..

[36] Sanon H.O., Kanwe A.B., Millogo A., Ledin I. (2013). Chemical Composition, Digestibility, and Voluntary Feed Intake of Mango Residues by Sheep. Trop. Anim. Health Prod..

[37] Avornyo F.K., Partey S.T., Zougmore R.B., Asare S., Agbolosu A.A., Akufo N.M., Sowah N.A., Konlan S.P. (2020). In Vivo Digestibility of Six Selected Fodder Species by Goats in Northern Ghana. Trop. Anim. Health Prod..

[38] Isah O.A., Okunade S.A., Aderinboye R.Y., Olafadehan O.A. (2015). Effect of Browse Plant Foliage Supplementation on the Performance of Buckling Goats Fed Threshed Sorghum Top Basal Diet. Trop. Anim. Health Prod..

[39] Sidi Imorou H., Babatounde S., Sidi Imorou F., Mensah G. (2016). Ligneux Fourragers Des Parcours Naturels Communautaires Du Nord-Bénin: Prédiction de La Valeur Nutritive Au Moyen de Plusieurs Approches Analytiques. J. Anim. Plant Sci..

[40] Sanou K.F., Ouédraogo S., Nacro S., Ouédraogo M., Kaboré-Zoungrana C. (2016). Durabilité de l’offre et valeur nutritive des fourrages commercialisés en zone urbaine de Bobo-Dioulasso, Burkina Faso. Cah. Agric..

[41] Kouadio K.P., Kouadja G.S., Fayama T., Soro S., Badou K.M.E. (2024). Caractérisation des espèces fourragères sur les marchés à bétail dans la commune de Bouaké (Côte d’Ivoire). Sci. Nat. Appl..

[42] Muftau M.A., Musa Z. (2020). Chemical Composition of Some Forages Fed to Ruminants in a Semi-Arid Environment of Kebbi State, Nigeria. Niger. J. Anim. Sci. Technol. (NJAST).

[43] Koura B.I., Yassegoungbe F.P., Afatondji C.U., Cândido M.J.D., Guimaraes V.P., Dossa L.H. (2021). Diversity and Nutritional Values of Leaves of Trees and Shrubs Used as Supplements for Goats in the Sub-Humid Areas of Benin (West Africa). Trop. Anim. Health Prod..

[44] Essen P.O., Njoku G.N., Onya G.U., Zachary B.N., Donkoh D.S., Kuka T.T., Adjei-Mensah B. (2025). Effect of Oil Palm Leaf Meal (*Elaeis guineensis*) on Growth Performance, Haematology and Carcass Characteristics of West African Dwarf Sheep. Vet. Med. Sci..

[45] Mawussi E., Tchaniley L., Nenonene A., Kulo A. (2022). Study of Forage Species of the Maritime Region of Togo Used in Livestock Feed. World J. Adv. Res. Rev..

[46] Osakwe I.I., Steingass H. (2006). Ruminal Fermentation and Nutrient Digestion in West African Dwarf (WAD) Sheep Fed *Leucaena leucocephala* Supplemental Diets. Agroforest Syst..

[47] Sasu P., Attoh-Kotoku V., Akorli D.E., Adjei-Mensah B., Tankouano R.A., Kwaku M. (2023). Nutritional Evaluation of the Leaves of *Oxytenanthera abyssinica*, *Bambusa balcooa*, *Moringa oleifera*, *Terminalia catappa*, *Blighia sapida,* and *Mangifera indica* as Non-Conventional Green Roughages for Ruminants. J. Agric. Food Res..

[48] Gbenou G.X., Hamidou S., Akpo Y., DJENONTIN J., Djenontin P., Sidi H., Babatounde S. (2020). Performances d’engraissement et Économique Des Taurillons Métis (Gir x Borgou) Complémentés Avec La Drêche Sèche de Sorgho Au Pâturage à Panicum Maximum C1 Dans Le Nord-Bénin. Afr. Sci. Rev. Int. Sci. Technol..

[49] Etela I., Larbi A., Bamikole M.A., Ikhatua U.J., Oji U.I. (2008). Rumen Degradation Characteristics of Sweet Potato Foliage and Performance by Local and Crossbred Calves Fed Milk and Foliage from Three Cultivars. Livest. Sci..

[50] Okagbare G.O., Akpodiete O.J., Esiekpe O., Onagbesan O.M. (2004). Evaluation of Gmelina Arborea Leaves Supplemented with Grasses (Panicum Maximum and Pennisetum Purpureum) as Feed for West African Dwarf Goats. Trop. Anim. Health Prod..

[51] Amegnaglo K., Diwediga B., Marra D., Akpavi S., Wala K., Komlan B., Gbandi D.-B., Koffi A. (2018). Biomasse Des Pâturages de La Plaine Du Mono Au Togo: Diversité, Valeurs Nutritionnelle et Fourragère. J. Rech. Sci. L’université Lomé.

[52] Patra A.K. (2010). Effects of Supplementing Low-Quality Roughages with Tree Foliages on Digestibility, Nitrogen Utilization and Rumen Characteristics in Sheep: A Meta-Analysis. J. Anim. Physiol. Anim. Nutr..

[53] Ansah T., Yaccub Z.I., Rahman N.A. (2017). Growth Performance and Hematology of Djallonké Rams Fed Haulms of Four Varieties of Groundnut (*Arachis hypogaea* L.). Anim. Nutr..

[54] Umutoni C., Bado V., Whitbread A., Ayantunde A., Gangashetty P. (2021). Evaluation of Chemical Composition and in Vitro Digestibility of Stovers of Different Pearl Millet Varieties and Their Effect on the Performance of Sheep in the West African Sahel. Acta Agric. Scand. Sect. A—Anim. Sci..

[55] Mutimura M., Ebong C., Rao I.M., Nsahlai I.V. (2015). Nutritional Values of Available Ruminant Feed Resources in Smallholder Dairy Farms in Rwanda. Trop. Anim. Health Prod..

[56] Rharad A.A., Aayadi S.E., Avril C., Souradjou A., Sow F., Camara Y., Hornick J.-L., Boukrouh S. (2025). Meta-Analysis of Dietary Tannins in Small Ruminant Diets: Effects on Growth Performance, Serum Metabolites, Antioxidant Status, Ruminal Fermentation, Meat Quality, and Fatty Acid Profile. Animals.

[57] Akinmoladun O. (2022). Effect of Processing Methods on Chemical and Nutrient Composition of Bamboo (*Bambusae arundinacea*) Leaves. J. Anim. Plant Sci..

[58] Njidda A. Chemical Composition, Fibre Fraction and Anti-Nutritional Substances of Semi-Arid Browse Forages of North-Eastern Nigeria. https://www.cabidigitallibrary.org/doi/full/10.5555/20113202227.

[59] Ogunbosoye D.O., Odedire J.A. (2022). Evaluation of Silage from Maize Stover, Maize Husk and Andropogon Gayanus in Equal Level with Tephrosia Bracteolata as Feed for West African Dwarf Sheep. Trop. Anim. Health Prod..

[60] Okoli I.C., Odoemene E.C., Ezenwata C.C., Ohanaka A.U.C., Odoemelam V.U. (2024). Forage Plants for Small Ruminant Feeding at Rural and Peri-Urban Sites in the Warm Humid Tropical Environment of Southeastern Nigeria. BOKU.

[61] Osakwe I.I., Steingass H., Drochner W. (2004). Effect of Dried *Elaeis guineense* Supplementation on Nitrogen and Energy Partitioning of WAD Sheep Fed a Basal Hay Diet. Anim. Feed Sci. Technol..

[62] Sana Y., Sangare M., Tingueri B.L., Sawadogo L., Kabore-Zoungrana C.Y. (2020). Effet de l’utilisation de quatre rations à base de Panicum maximum C1 sur les performances zootechniques des ovins de race Djallonké, à l’Ouest du Burkina Faso. Revue RAMReS.

[63] Sasu P., Edinam Akorli D., Asare R., Attoh-Kotoku V., Adjei O., Adjei-Mensah B., Adjima Tankouano R., Kweku Mintah F., Anim-Jnr A.S., Kwaku M. (2023). Comparative Nutritional Evaluation of the Leaves of *Bambusa balcooa* (Beema) and *Oxytenanthera abyssinica* (A. Rich.) Munro Bamboos, and the Straws of AGRA and AMANKWATIA Rice Varieties. Cogent Food Agric..

[64] Sissao M., Millogo V., Sidibe/Anago A.G., Djikoldingam R.R., Kere M. (2024). Valeurs bromatologiques des fourrages ensilés en fûts plastiques et consommation volontaire chez les chèvres Djallonké au Burkina Faso. Sci. Nat. Appl..

[65] Yashim S.M., Abdu S.B., Hassan M. (2012). Effect of Processing Methods on the Degradability of Rattle Box (*Crotalaria retusa*) Plant in Yankasa Rams. J. Appl. Anim. Res..

