# Supplementary material for: Chemical Composition Tables of Locally Available Ruminant Feeds in West Africa: A Systematic Review
Source: Animals (Basel). 2026 Apr 16;16(8):1215. doi: 10.3390/ani16081215 (PMC13113009; doi:10.3390/ani16081215)
Supplement: Supplementary file 1 [file animals-16-01215-s001.zip › animals-4185770-supplementary.pdf]

Table S1. Full search strings used in Scopus, Web of Science, and Google Scholar

**1. Scopus**	TITLE-ABS-KEY(("chemical composition" OR "nutritional value" OR "feed analysis" OR "feed composition") AND ("ruminant" OR "cattle" OR "sheep" OR "goat*" OR "livestock") AND ("locally available" OR "local" OR "regional") AND ("feed" OR "forage" OR "diet") AND ("Benin" OR "Burkina Faso" OR "Cape Verde" OR "Côte d'Ivoire" OR "Gambia" OR "Ghana" OR "Guinea" OR "Guinea-Bissau" OR "Liberia" OR "Mali" OR "Niger" OR "Nigeria" OR "Senegal" OR "Sierra Leone" OR "Togo"))
**2. Web of Science**	TS=(("chemical composition" OR "nutritional value" OR "feed analysis" OR "feed composition") AND ("ruminant" OR "cattle" OR "sheep" OR "goat*" OR "livestock") AND ("locally available" OR "local" OR "regional") AND ("feed" OR "forage" OR "diet") AND ("Benin" OR "Burkina Faso" OR "Cape Verde" OR "Côte d'Ivoire" OR "Gambia" OR "Ghana" OR "Guinea" OR "Guinea-Bissau" OR "Liberia" OR "Mali" OR "Niger" OR "Nigeria" OR "Senegal" OR "Sierra Leone" OR "Togo"))
**3. Google Scholar**	"chemical composition" OR "nutritional value" OR "feed analysis" OR "feed composition" AND "ruminant" OR "cattle" OR "sheep" OR "goat" OR "livestock" AND "feed" OR "forage" OR "diet" AND "West Africa"<br>**Note:** Because Google Scholar handles Boolean operators differently from Scopus and Web of Science, keyword combinations were used without strict Boolean nesting, and the first 200 results ranked by relevance were screened.

French keywords also used: "composition chimique" OR "composition des aliments" "ruminant" OR "bovin" OR "ovin" OR "caprin" "sous-produits agro-industriels" OR "sous-produits agricoles" OR "fourrages"

Table S2. List of studies included in the systematic review and used for feed composition data extraction in West Africa

ID	**Authors**	**Title**	**Year**	**Source title**	**DOI**	**Country**
1	Abdou, N.; Nsahlai, I.V.; Chimonyo, M.	Effects of groundnut haulms supplementation on millet stover intake, digestibility and growth performance of lambs	2011	Animal Feed Science and Technology	10.1016/j.anifeedsci.2011.07.002	Niger
2	Akinmoladun O.F.	Effect of Processing Methods on Chemical and Nutrient Composition of Bamboo (Bambusae arundinacea) Leaves	2022	Journal of Animal and Plant Sciences	10.36899/JAPS.2022.2.0429	Nigeria
3	Amegnaglo K.B.; Dourma M.; Akpavi S.; Diwediga B.; Wala K.; Batawila K.; Djaneye-Boundjou G.; Akpagana K.	Biomasse des pâturages de la plaine du Mono au Togo : diversité, valeurs nutritionnelle et fourragère	2018	Journal de la Recherche Scientifique de l'Université de Lomé	—	Togo
4	Amole T.A.; Panyan E.; Adekeye A.; Ayantunde A.; Duncan A.; Blummel M.	Productivity nutritive value and economic potential of irrigated fodder in two regions of Ghana	2022	Agronomy Journal	10.1002/agj2.20884	Ghana
5	Anele U.Y.; Südekum K.-H.; Arigbede O.M.; Lüttgenau H.; Oni A.O.; Bolaji O.J.; Galyean M.L.	Chemical characterization, in vitro dry matter and ruminal crude protein degradability and microbial protein synthesis of some cowpea (Vigna unguiculata L. Walp) haulm varieties	2012	Grass and Forage Science	10.1111/j.1365-2494.2011.00835.x	Nigeria
6	Ansah T.; Yaccub Z.I.; Rahman N.A.	Growth performance and hematology of Djallonké rams fed haulms of four	2017	Animal Nutrition	10.1016/j.aninu.2017.08.006	Ghana

		varieties of groundnut (Arachis hypogaea L.)				
7	Anyanwu N.J.; Etela I.	Chemical composition and dry matter degradation characteristics of multi-purpose trees and shrubs in the humid lowlands of southeastern Nigeria	2013	Agroforestry Systems	10.1007/s10457-012-9593-6	Nigeria
8	Avornyo F.K.; Partey S.T.; Zougmore R.B.; Asare S.; Agbolosu A.A.; Akufo N.M.; Sowah N.A.; Konlan S.P.	In vivo digestibility of six selected fodder species by goats in northern Ghana	2020	Tropical Animal Health and Production	10.1007/s11250-019-01989-w	Ghana
9	Dokui F.; Houndonougbo F.M.; Djidda S.G.; Houndonougbo V.P.; Gangbedji E.; Agbo G.M.; Dedome S.L.; Babatoundé S.; Toleba S.S.; Chrysostome C.A.A.M.	Milk Yield of Borgou Cows Improved with Lick Stones made in Benin	2023	Advances in Animal and Veterinary Sciences	10.17582/journal.aavs/2023/11.3.424.430	Benin
10	Essen P.O.; Njoku G.N.; Onya G.U.; Zachary B.N.; Donkoh D.S.; Kuka T.T.; Adjei-Mensah B.	Effect of Oil Palm Leaf Meal (Elaeis guineensis) on Growth Performance, Haematology and Carcass Characteristics of West African Dwarf Sheep	2025	Veterinary Medicine and Science	10.1002/vms3.70581	Ghana
11	Etela I.; Larbi A.; Bamikole M.A.;	Rumen degradation characteristics of sweet potato foliage and performance	2008	Livestock Science	10.1016/j.livsci.2007.06.004	Nigeria

	Ikhatua U.J.; Oji U.I.	by local and crossbred calves fed milk and foliage from three cultivars				
12	Gbenou G.X.; Hamidou S.; Akpo Y.; Djenontin P.; Sidi H.; Babatounde S.	Performances d'engraissement et économique des taurillons métis (Gir x Borgou) complémentés avec la drêche sèche de sorgho au pâturage à Panicum maximum C1 dans le Nord-Bénin	2020	Afrique SCIENCE	—	Benin
13	Idan F.; Adogla-Bessa T.; Sarkwa F.O.; Frimpong Y.O.; Antwi C.	Effects of supplementing rice straw with two fodder tree leaves and their combinations on voluntary feed intake, growth, and nitrogen utilization in sheep	2023	Translational Animal Science	10.1093/tas/txad004	Ghana
14	Idrissou Y.; Assani S.A.; Alkoiret I.T. Mensah A.G	Performances d'embouche des ovins Djallonké complémentés avec les fourrages de Gliricidia sepium et de Leucaena leucocephala au Centre du Bénin	2017	BRAB	—	Benin
15	Isah O.A.; Okunade S.A.; Aderinboye R.Y.; Olafadehan O.A.	Effect of browse plant foliage supplementation on the performance of buckling goats fed threshed sorghum top basal diet	2015	Tropical Animal Health and Production	10.1007/s11250-015-0823-6	Nigeria
16	Kiéma Sébastien; Kini Louis; Ouédraogo Salifou; Kaboré/Zoungrana Chantal Yvette	Effet de l'utilisation des gousses de Faidherbia albida sur les performances de croissance des taurillons à l'Ouest du Burkina Faso	2019	Science et technique, Sciences naturelles et appliquées	—	Burkina Faso
17	Kondombo S.R.; Niannongo A.J.	Perfomance d'ovins Djallonké alimentés à base de résidus de récolte au Burkina Faso	2001	Agronomie Africaine	—	Burkina Faso
18	Kouadio Kouakou Parfait; Kouadja	Caractérisation des espèces fourragères sur les marchés à bétail	2024	Sciences Naturelles et Appliquées	—	Côte d'Ivoire

	Gouagoua Sévérin; Fayama Tionyélé; Soro Soronikpo; Badou Krah Murielle Etith	dans la commune de Bouaké (Côte d'Ivoire)				
19	Koura B.I.; Yassegoungbe F.P.; Afatondji C.U.; Cândido M.J.D.; Guimaraes V.P.; Dossa L.H.	Diversity and nutritional values of leaves of trees and shrubs used as supplements for goats in the sub-humid areas of Benin (West Africa)	2021	Tropical Animal Health and Production	10.1007/s11250-021-02559-9	Benin
20	Koura B.I.; Vastolo A.; Kiatti D.D.; Cutrignelli M.I.; Houinato M.; Calabrò S.	Nutritional Value of Climate-Resilient Forage Species Sustaining Peri-Urban Dairy Cow Production in the Coastal Grasslands of Benin (West Africa)	2022	Animals	10.3390/ani12243550	Benin
21	Lamidi A.; Ogunkunle T.	Chemical composition, mineral profile and phytochemical properties of common feed resources used for small ruminant animal production in south-west, Nigeria	2015	International Journal of Design and Nature and Ecodynamics	—	Nigeria
22	Mahamadou Abdou Malam; Abdou Gomma Dan; Salissou Issa; Gambo Yahoussa; Moctar Karimou; Salifou Bagnan; Yaou Zakari Moussa	Performance zootechnique des jeunes ovins mâles nourris en complémentation au résidu de moringa (Moringa oleifera Lam.) au Niger	2022	International Journal of Biological and Chemical Sciences	10.4314/ijbcs.v15i5.28	Niger

23	Mawussi E.B.; Tchaniley L.; Nenonene A.Y.; Kulo A.	Study of forage species of the maritime region of Togo used in livestock feed	2022	World Journal of Advanced Research and Reviews	10.30574/wjarr.2022.15.3.0869	Togo
24	Montcho M.; Babatoundé S.; Aboh B.A.; Bahini M.J.D.; Chrysostome A.A.M.C.; Mensah G.A.	Disponibilite, Valeurs Marchande Et Nutritionnelle Des Sous-Produits Agricoles Et Agroindustriels Utilises Dans L'alimentation Des Ruminants Au Benin	2016	European Scientific Journal	10.19044/esj.2016.v12n33p422	Benin
25	Muftau M.A.; Musa Z.	Chemical composition of some forages fed to ruminants in a Semi-arid environment of Kebbi State, Nigeria	2020	Nigerian Journal of Animal Science and Technology	—	Nigeria
26	Nantoumé H.; Kouriba A.; Togola D.; Ouologuem B.	Mesure de la valeur alimentaire de fourrages et de sous-produits utilisés dans l'alimentation des petits ruminants	2000	Revue d'Élevage et de Médecine Vétérinaire des Pays Tropicaux	—	Mali
27	Njidda A.	Chemical Composition, Fibre Fraction and Anti-Nutritional Substances of Semi-arid Browse Forages of North-Eastern Nigeria	2011	Nigerian Journal of Basic and Applied Science	10.4314/njbas.v18i2.64308	Nigeria
28	Oduguwa B.O.; Oni A.O.; Arigbede O.M.; Adesunbola J.O.; Südekum K.H.	Feeding potential of cassava (Manihot esculenta Crantz) peels ensiled with Leucaena leucocephala and Gliricidia sepium assessed with West African dwarf goats	2013	Tropical Animal Health and Production	10.1007/s11250-013-0370-y	Nigeria
29	Ogunbosoye D.O.; Odedire J.A.	Evaluation of silage from maize stover, maize husk and Andropogon gayanus in equal level with Tephrosia bracteolata as feed for West African Dwarf sheep	2022	Tropical Animal Health and Production	10.1007/s11250-022-03149-z	Nigeria

30	Okagbare G.O.; Akpodiete O.J.; Esiekpe O.; Onagbesan O.M.	Evaluation of Gmelina arborea leaves supplemented with grasses (Panicum maximum and Pennisetum purpureum) as feed for West African Dwarf goats	2004	Tropical Animal Health and Production	10.1023/B:TROP.0000040937.42739.c6	Nigeria
31	Okoli I.C.; Odoemene E.C.; Ezenwata C.C.; Chinenye Ohanaka A.U.; Odoemelam V.U.	Forage Plants for Small Ruminant Feeding at Rural and Peri-Urban Sites in the Warm Humid Tropical Environment of Southeastern Nigeria	2024	Bodenkultur	10.2478/boku-2024-0005	Nigeria
32	Osakwe I.I.; Steingass H.	Ruminal fermentation and nutrient digestion in West African Dwarf (WAD) sheep fed Leucaena leucocephala supplemental diets	2006	Agroforestry Systems	10.1007/s10457-005-7474-y	Nigeria
33	Osakwe I.I.; Steingass H.; Drochner W.	Effect of dried Elaeis guineense supplementation on nitrogen and energy partitioning of WAD sheep fed a basal hay diet	2004	Animal Feed Science and Technology	10.1016/j.anifeedsci.2004.07.010	Nigeria
34	Sana, Y., Sangare, M., Tingueri, B.L., Sawadogo, L., Kabore-Zoungrana, C.Y.,	Effet de l’utilisation de quatre rations à base de Panicum maximum C1 sur les performances zootechniques des ovins de race Djallonké, à l’Ouest du Burkina Faso	2020	Revue RAMReS - Sciences de la vie, de la terre et agronomie	—	Burkina Faso
35	Sanogo O.M.; Doumbia S.; Descheemaeker K.	Complémentation des bovins laitiers pour l’amélioration de la production de lait et du fumier en milieu paysan dans le cercle de Koutiala	2019	Revue Malienne de Science et de Technologie	—	Mali
36	Sanon H.O.; Kanwe A.B.; Millogo A.; Ledin I.	Chemical composition, digestibility, and voluntary feed intake of mango residues by sheep	2013	Tropical Animal Health and Production	10.1007/s11250-012-0275-1	Burkina Faso

37	Sanou K.F.; Ouédraogo S.; Nacro S.; Ouédraogo M.; Kaboré-Zoungrana C.	Durabilité de l'offre et valeur nutritive des fourrages commercialisés en zone urbaine de Bobo-Dioulasso, Burkina Faso	2016	Cahiers Agricultures	10.1051/cagri/2016007	Burkina Faso
38	Sasu P.; Attoh-Kotoku V.; Akorli D.E.; Adjei-Mensah B.; Tankouano R.A.; Kwaku M.	Nutritional evaluation of the leaves of Oxytenanthera abyssinica, Bambusa balcooa, Moringa oleifera, Terminalia catappa, Blighia sapida, and Mangifera indica as non-conventional green roughages for ruminants	2023	Journal of Agriculture and Food Research	10.1016/j.jafr.2022.100466	Ghana
39	Sasu P.; Edinam Akorli D.; Asare R.; Attoh-Kotoku V.; Adjei O.; Adjei-Mensah B.; Adjima Tankouano R.; Kweku Mintah F.; Anim-Jnr A.S.; Kwaku M.; Owusu Ansah T.	Comparative nutritional evaluation of the leaves of Bambusa balcooa (Beema) and Oxytenanthera abyssinica (A. Rich.) Munro bamboos, and the straws of AGRA and AMANKWATIA rice varieties	2023	Cogent Food and Agriculture	10.1080/23311932.2023.2263960	Ghana
40	Sidibé S.; Tangara M.; Cissé S.M.; Doumbia S.; Maïga A.M.; Mallé B.; Nantoumé H.	Effets de la fane de Cassia tora sur les performances zootechniques des béliers Djallonké en station	2019	Revue Malienne des Sciences et de la Technologie	—	Mali
41	Sidiimorou H.; Babatoundé S.; Sidiimorou F.; Mensah G.A.	Ligneux fourragers des parcours naturels communautaires du Nord-Bénin : prédiction de la valeur	2016	Journal of Animal & Plant Sciences	—	Benin

		nutritive au moyen de plusieurs approches analytiques				
42	Sissao Mariétou; Millogo Vinsoun; Sidibe/Anago Alice Gisèle; Djikoldingam Remadji Rufine; Kere Michel	Valeurs bromatologiques des fourrages ensilés en fûts plastiques et consommation volontaire chez les chèvres Djallonké au Burkina Faso	2024	Sciences Naturelles et Appliquées	—	Burkina Faso
43	Umutoni C.; Bado V.; Whitbread A.; Ayantunde A.; Gangashetty P.	Evaluation of chemical composition and in vitro digestibility of stovers of different pearl millet varieties and their effect on the performance of sheep in the West African Sahel	2021	Acta Agriculturae Scandinavica A: Animal Sciences	10.1080/09064702.2021.1919193	Niger
44	Yashim S.M.; Abdu S.B.; Hassan M.	Effect of processing methods on the degradability of rattle box (Crotalaria retusa) plant in Yankasa rams	2012	Journal of Applied Animal Research	—	Nigeria

Table S3. Chemical composition of agro-industrial by-products for ruminants in West-Africa

Feed	DM (%)	EE (% DM)	OM (% DM)	dOM (% DM)	CP (% DM)	CF (% DM)	Ash (% DM)	GE (MJ/kg DM)	DE (MJ/kg DM)	ME (MJ/kg DM)	DCP (g/kg DM)	UFL	UFV	NDF (% DM)	ADF (% DM)	Ca (% DM)	P (% DM)	References
Banana peels	85.99	4.59	85.13	75.94	6.66	9.84	14.87	16.07	-	-	3.37	1.08	1.03	53.29	46.99	-	-	Montcho et al., 2016
Average	85.99	4.59	85.13	75.94	6.66	9.84	14.87	16.07	-	-	3.37	1.08	1.03	53.29	46.99	-	-	Montcho et al., 2016 (n=1)
Minimum	85.99	4.59	85.13	75.94	6.66	9.84	14.87	16.07	-	-	3.37	1.08	1.03	53.29	46.99	-	-	
Maximum	85.99	4.59	85.13	75.94	6.66	9.84	14.87	16.07	-	-	3.37	1.08	1.03	53.29	46.99	-	-	
Standard deviation	0	0	0	0	0	0	0	0	-	-	0	0	0	0	0	-	-	
CV (%)	0	0	0	0	0	0	0	0	-	-	0	0	0	0	0	-	-	
Breadfruit peels	93.11	1.99	92.47	73.91	7.12	8.76	7.73	17.08	-	-	3.8	1.02	1	69.43	50.77	-	-	Montcho et al., 2016
Average	93.11	1.99	92.47	73.91	7.12	8.76	7.73	17.08	-	-	3.8	1.02	1	69.43	50.77	-	-	Montcho et al., 2016 (n=1)
Minimum	93.11	1.99	92.47	73.91	7.12	8.76	7.73	17.08	-	-	3.8	1.02	1	69.43	50.77	-	-	
Maximum	93.11	1.99	92.47	73.91	7.12	8.76	7.73	17.08	-	-	3.8	1.02	1	69.43	50.77	-	-	
Standard deviation	0	0	0	0	0	0	0	0	-	-	0	0	0	0	0	-	-	
CV (%)	0	0	0	0	0	0	0	0	-	-	0	0	0	0	0	-	-	
Brewers' grains	86.4	-	82.7	-	24.06	-	17.3	-	-	-	-	-	-	64.9	-	-	-	Etela et al., 2008
Average	86.4	-	82.7	-	24.06	-	17.3	-	-	-	-	-	-	64.9	-	-	-	Etela et al., 2008 (n=1)
Minimum	86.4	-	82.7	-	24.06	-	17.3	-	-	-	-	-	-	64.9	-	-	-	
Maximum	86.4	-	82.7	-	24.06	-	17.3	-	-	-	-	-	-	64.9	-	-	-	
Standard deviation	0	-	0	-	0	-	0	-	-	-	-	-	-	0	-	-	-	
CV (%)	0	-	0	-	0	-	0	-	-	-	-	-	-	0	-	-	-	
Sorghum brewers grains	93.98	10.13	81.87	-	19.5	15.15	13.7	-	-	-	-	-	-	64.74	50.9	-	-	Montcho et al., 2016
Average	93.98	10.13	81.87	-	19.5	15.15	13.7	-	-	-	-	-	-	64.74	50.9	-	-	Montcho et al., 2016 (n=1)
Minimum	93.98	10.13	81.87	-	19.5	15.15	13.7	-	-	-	-	-	-	64.74	50.9	-	-	
Maximum	93.98	10.13	81.87	-	19.5	15.15	13.7	-	-	-	-	-	-	64.74	50.9	-	-	
Standard deviation	0	0	0	-	0	0	0	-	-	-	-	-	-	0	0	-	-	
CV (%)	0	0	0	-	0	0	0	-	-	-	-	-	-	0	0	-	-	
Cassava peels	89.82	4.12	-	-	5.32	16.84	8.94	12.84488	-	-	-	-	-	47.84	21.32	-	-	Lamidi and Ogunkunle, 2015
Cassava peels	92.75	1.8	94.67	68.44	5.26	9.96	5.45	17.14	-	-	-	0.93	0.89	60.89	49.36	0.45	0.8	Montcho et al., 2016
Cassava peels	-	-	94.7	-	5.43	-	5.3	19.59	-	-	-	-	-	53.8	25.4	-	-	Oduguwa et al., 2013
Average	91.28	2.96	94.69	68.44	5.34	13.4	6.56	16.52	-	-	-	0.93	0.89	54.18	32.03	0.45	0.8	Lamidi and Ogunkunle, 2015; Montcho et al., 2016; Oduguwa et al., 2013 (n=3)
Minimum	89.82	1.8	94.67	68.44	5.26	9.96	5.3	12.84	-	-	-	0.93	0.89	47.84	21.32	0.45	0.8	
Maximum	92.75	4.12	94.7	68.44	5.43	16.84	8.94	19.59	-	-	-	0.93	0.89	60.89	49.36	0.45	0.8	
Standard deviation	2.07	1.64	0.02	0	0.09	4.86	2.06	3.41	-	-	-	0	0	6.53	15.15	0	0	
CV (%)	2.27	55.42	0.02	0	1.62	36.31	31.38	20.66	-	-	-	0	0	12.06	47.3	0	0	
Ensiled cassava peels	-	-	93.6	-	9.85	-	6.42	14.49	-	-	-	-	-	39.2	17.7	-	-	Oduguwa et al., 2013
Average	-	-	93.6	-	9.85	-	6.42	14.49	-	-	-	-	-	39.2	17.7	-	-	Oduguwa et al., 2013 (n=1)
Minimum	-	-	93.6	-	9.85	-	6.42	14.49	-	-	-	-	-	39.2	17.7	-	-	
Maximum	-	-	93.6	-	9.85	-	6.42	14.49	-	-	-	-	-	39.2	17.7	-	-	
Standard deviation	-	-	0	-	0	-	0	0	-	-	-	-	-	0	0	-	-	
CV (%)	-	-	0	-	0	-	0	0	-	-	-	-	-	0	0	-	-	
Cocoa pod	85.99	0.97	92.61	60.12	31.97	38.56	7.55	19.56	-	-	26.53	0.77	0.67	91.3	66.39	-	-	Montcho et al., 2016
Average	85.99	0.97	92.61	60.12	31.97	38.56	7.55	19.56	-	-	26.53	0.77	0.67	91.3	66.39	-	-	Montcho et al., 2016 (n=1)

Minimum	85.99	0.97	92.61	60.12	31.97	38.56	7.55	19.56	-	-	26.53	0.77	0.67	91.3	66.39	-	-	
Maximum	85.99	0.97	92.61	60.12	31.97	38.56	7.55	19.56	-	-	26.53	0.77	0.67	91.3	66.39	-	-	
Standard deviation	0	0	0	0	0	0	0	0	-	-	0	0	0	0	0	-	-	
CV (%)	0	0	0	0	0	0	0	0	-	-	0	0	0	0	0	-	-	
Copra cake	91.9	44.89	94.41	-	22.94	0.96	5.4	-	-	-	-	-	-	65.15	58.32	-	-	Montcho et al., 2016
Average	91.9	44.89	94.41	-	22.94	0.96	5.4	-	-	-	-	-	-	65.15	58.32	-	-	Montcho et al., 2016 (n=1)
Minimum	91.9	44.89	94.41	-	22.94	0.96	5.4	-	-	-	-	-	-	65.15	58.32	-	-	
Maximum	91.9	44.89	94.41	-	22.94	0.96	5.4	-	-	-	-	-	-	65.15	58.32	-	-	
Standard deviation	0	0	0	-	0	0	0	-	-	-	-	-	-	0	0	-	-	
CV (%)	0	0	0	-	0	0	0	-	-	-	-	-	-	0	0	-	-	
Cotton seeds	90.1	21.3	95.3	-	18.5	30.3	4.7	-	-	-	157	0.92	-	-	-	-	-	Idrissou et al., 2017
Cotton seeds	91.5	-	-	-	25.5	25.3	-	-	-	-	142	1.19	-	-	-	0.17	0.76	Sanogo et al., 2019
Average	90.8	21.3	95.3	-	22	27.8	4.7	-	-	-	149.5	1.05	-	-	-	0.17	0.76	Idrissou et al., 2017; Sanogo et al., 2019 (n=2)
Minimum	90.1	21.3	95.3	-	18.5	25.3	4.7	-	-	-	142	0.92	-	-	-	0.17	0.76	
Maximum	91.5	21.3	95.3	-	25.5	30.3	4.7	-	-	-	157	1.19	-	-	-	0.17	0.76	
Standard deviation	0.99	0	0	-	4.95	3.54	0	-	-	-	10.61	0.19	-	-	-	0	0	
CV (%)	1.09	0	0	-	22.5	12.72	0	-	-	-	7.09	18.1	-	-	-	0	0	
Cottonseed cake	92.9	16.85	92.68	-	33.43	13.57	7.35	-	-	-	-	-	-	63.45	51.37	-	-	Montcho et al., 2016
Cottonseed cake	92.9	-	-	-	45.8	45.8	-	-	-	-	426	1.04	-	-	-	0.28	1.21	Sanogo et al., 2019
Cottonseed cake	95.49	10.17	-	-	41.63	15.1	6.77	-	-	-	-	-	0.82	28.67	21.14	-	-	Kondombo and Niannongo, 2001
Cottonseed cake	86.35	11.62	-	-	21.94	28.54	4.59	7.8	-	-	-	-	-	-	-	-	-	Sidibé et al., 2019
Cottonseed cake	86	33.43	92.68	-	19.2	-	7	-	-	-	-	-	-	-	-	-	-	Dokui et al., 2023
Average	90.73	18.02	92.68	-	32.4	25.75	6.43	7.8	-	-	426	1.04	0.82	46.06	36.25	0.28	1.21	Montcho et al., 2016; Sanogo et al., 2019; Kondombo and Niannongo, 2001; Sidibé et al., 2019; Dokui et al., 2023 (n=5)
Minimum	86	10.17	92.68	-	19.2	13.57	4.59	7.8	-	-	426	1.04	0.82	28.67	21.14	0.28	1.21	
Maximum	95.49	33.43	92.68	-	45.8	45.8	7.35	7.8	-	-	426	1.04	0.82	63.45	51.37	0.28	1.21	
Standard deviation	4.29	10.67	0	-	11.72	14.96	1.25	0	-	-	0	0	0	24.59	21.38	0	0	
CV (%)	4.73	59.21	0	-	36.17	58.1	19.42	0	-	-	0	0	0	53.39	58.96	0	0	
Cowpea bran	92.07	0.97	96.04	78.49	17.29	14.87	3.96	18.72	-	-	13.09	1.07	1.04	76.96	53.64	-	-	Montcho et al., 2016
Average	92.07	0.97	96.04	78.49	17.29	14.87	3.96	18.72	-	-	13.09	1.07	1.04	76.96	53.64	-	-	Montcho et al., 2016 (n=1)
Minimum	92.07	0.97	96.04	78.49	17.29	14.87	3.96	18.72	-	-	13.09	1.07	1.04	76.96	53.64	-	-	
Maximum	92.07	0.97	96.04	78.49	17.29	14.87	3.96	18.72	-	-	13.09	1.07	1.04	76.96	53.64	-	-	
Standard deviation	0	0	0	0	0	0	0	0	-	-	0	0	0	0	0	-	-	
CV (%)	0	0	0	0	0	0	0	0	-	-	0	0	0	0	0	-	-	
Garigo	90.6	0.91	99.92	73.46	1.69	2.15	0.92	17.29	-	-	-	1.01	0.98	66.77	57.77	-	-	Montcho et al., 2016
Average	90.6	0.91	99.92	73.46	1.69	2.15	0.92	17.29	-	-	-	1.01	0.98	66.77	57.77	-	-	Montcho et al., 2016 (n=1)
Minimum	90.6	0.91	99.92	73.46	1.69	2.15	0.92	17.29	-	-	-	1.01	0.98	66.77	57.77	-	-	
Maximum	90.6	0.91	99.92	73.46	1.69	2.15	0.92	17.29	-	-	-	1.01	0.98	66.77	57.77	-	-	
Standard deviation	0	0	0	0	0	0	0	0	-	-	-	0	0	0	0	-	-	
CV (%)	0	0	0	0	0	0	0	0	-	-	-	0	0	0	0	-	-	
Groundnut cake	90.97	0.89	93.94	-	54.28	16.5	6.11	-	-	-	-	1.12	-	39.67	17.91	-	-	Montcho et al., 2016
Average	90.97	0.89	93.94	-	54.28	16.5	6.11	-	-	-	-	1.12	-	39.67	17.91	-	-	Montcho et al., 2016 (n=1)
Minimum	90.97	0.89	93.94	-	54.28	16.5	6.11	-	-	-	-	1.12	-	39.67	17.91	-	-	
Maximum	90.97	0.89	93.94	-	54.28	16.5	6.11	-	-	-	-	1.12	-	39.67	17.91	-	-	
Standard deviation	0	0	0	-	0	0	0	-	-	-	-	0	-	0	0	-	-	

CV (%)	0	0	0	-	0	0	0	-	-	-	-	0	-	0	0	-	-	
Maize bran	92.75	1.6	94.93	-	14.84	6.7	5.13	-	-	-	-	-	-	65.56	62.61	-	-	Montcho et al., 2016
Maize bran	93.04	-	87.9	-	6.63	16.5	5.14	-	-	-	26.37	-	-	34.69	6.49	-	-	Kiema et al., 2019
Average	92.9	1.6	91.42	-	10.73	11.6	5.13	-	-	-	26.37	-	-	50.12	34.55	-	-	Montcho et al., 2016; Kiema et al., 2019 (n=2)
Minimum	92.75	1.6	87.9	-	6.63	6.7	5.13	-	-	-	26.37	-	-	34.69	6.49	-	-	
Maximum	93.04	1.6	94.93	-	14.84	16.5	5.14	-	-	-	26.37	-	-	65.56	62.61	-	-	
Standard deviation	0.21	0	4.97	-	5.81	6.93	0.01	-	-	-	0	-	-	21.83	39.68	-	-	
CV (%)	0.22	0	5.44	-	54.08	59.74	0.14	-	-	-	0	-	-	43.55	114.86	-	-	
Mango peels	92.2	4.7	94	-	6.7	-	6	-	-	-	-	-	-	38.8	-	-	-	Sanon et al., 2013
Average	92.2	4.7	94	-	6.7	-	6	-	-	-	-	-	-	38.8	-	-	-	Sanon et al., 2013 (n=1)
Minimum	92.2	4.7	94	-	6.7	-	6	-	-	-	-	-	-	38.8	-	-	-	
Maximum	92.2	4.7	94	-	6.7	-	6	-	-	-	-	-	-	38.8	-	-	-	
Standard deviation	0	0	0	-	0	-	0	-	-	-	-	-	-	0	-	-	-	
CV (%)	0	0	0	-	0	-	0	-	-	-	-	-	-	0	-	-	-	
Mango seed kernels	92.7	10.5	95.8	-	7.05	-	4.2	-	-	-	-	-	-	30.6	-	-	-	Sanon et al., 2013
Average	92.7	10.5	95.8	-	7.05	-	4.2	-	-	-	-	-	-	30.6	-	-	-	Sanon et al., 2013 (n=1)
Minimum	92.7	10.5	95.8	-	7.05	-	4.2	-	-	-	-	-	-	30.6	-	-	-	
Maximum	92.7	10.5	95.8	-	7.05	-	4.2	-	-	-	-	-	-	30.6	-	-	-	
Standard deviation	0	0	0	-	0	-	0	-	-	-	-	-	-	0	-	-	-	
CV (%)	0	0	0	-	0	-	0	-	-	-	-	-	-	0	-	-	-	
Millet bran	95.3	-	93.9	-	13.75	-	-	-	-	-	-	-	-	38.3	7.1	0.05	0.61	Abdou et al., 2011
Millet bran	92.3	-	-	-	13.8	4.6	-	-	-	-	90	0.86	-	-	-	0.08	0.48	Sanogo et al., 2019
Average	93.8	-	93.9	-	13.78	4.6	-	-	-	-	90	0.86	-	38.3	7.1	0.07	0.54	Abdou et al., 2011; Sanogo et al., 2019 (n=2)
Minimum	92.3	-	93.9	-	13.75	4.6	-	-	-	-	90	0.86	-	38.3	7.1	0.05	0.48	
Maximum	95.3	-	93.9	-	13.8	4.6	-	-	-	-	90	0.86	-	38.3	7.1	0.08	0.61	
Standard deviation	2.12	-	0	-	0.04	0	-	-	-	-	0	0	-	0	0	0.02	0.09	
CV (%)	2.26	-	0	-	0.26	0	-	-	-	-	0	0	-	0	0	32.64	16.87	
Nere powder	88.59	0.96	95.15	84.97	4.49	10.82	5	17.27	-	-	1.53	1.21	1.11	33.8	24.31	-	-	Montcho et al., 2016
Average	88.59	0.96	95.15	84.97	4.49	10.82	5	17.27	-	-	1.53	1.21	1.11	33.8	24.31	-	-	Montcho et al., 2016 (n=1)
Minimum	88.59	0.96	95.15	84.97	4.49	10.82	5	17.27	-	-	1.53	1.21	1.11	33.8	24.31	-	-	
Maximum	88.59	0.96	95.15	84.97	4.49	10.82	5	17.27	-	-	1.53	1.21	1.11	33.8	24.31	-	-	
Standard deviation	0	0	0	0	0	0	0	0	-	-	0	0	0	0	0	-	-	
CV (%)	0	0	0	0	0	0	0	0	-	-	0	0	0	0	0	-	-	
Orange peels	92.89	2.88	90.35	74.24	6.87	14.03	9.84	17.05	-	-	3.54	1.03	1.01	63.22	41.19	-	-	Montcho et al., 2016
Average	92.89	2.88	90.35	74.24	6.87	14.03	9.84	17.05	-	-	3.54	1.03	1.01	63.22	41.19	-	-	Montcho et al., 2016 (n=1)
Minimum	92.89	2.88	90.35	74.24	6.87	14.03	9.84	17.05	-	-	3.54	1.03	1.01	63.22	41.19	-	-	
Maximum	92.89	2.88	90.35	74.24	6.87	14.03	9.84	17.05	-	-	3.54	1.03	1.01	63.22	41.19	-	-	
Standard deviation	0	0	0	0	0	0	0	0	-	-	0	0	0	0	0	-	-	
CV (%)	0	0	0	0	0	0	0	0	-	-	0	0	0	0	0	-	-	
Palm kernel cake	93.06	22.05	95.59	-	19.78	10.49	4.45	-	-	-	-	-	-	62.76	43.84	-	-	Montcho et al., 2016
Average	93.06	22.05	95.59	-	19.78	10.49	4.45	-	-	-	-	-	-	62.76	43.84	-	-	Montcho et al., 2016 (n=1)
Minimum	93.06	22.05	95.59	-	19.78	10.49	4.45	-	-	-	-	-	-	62.76	43.84	-	-	
Maximum	93.06	22.05	95.59	-	19.78	10.49	4.45	-	-	-	-	-	-	62.76	43.84	-	-	
Standard deviation	0	0	0	-	0	0	0	-	-	-	-	-	-	0	0	-	-	
CV (%)	0	0	0	-	0	0	0	-	-	-	-	-	-	0	0	-	-	

Papaya peels	91.13	3.15	68.77	65.26	1.77	8.95	31.3	12.58	-	-	0	0.97	0.91	92.96	56.19	-	-	Montcho et al., 2016
Average	91.13	3.15	68.77	65.26	1.77	8.95	31.3	12.58	-	-	0	0.97	0.91	92.96	56.19	-	-	Montcho et al., 2016 (n=1)
Minimum	91.13	3.15	68.77	65.26	1.77	8.95	31.3	12.58	-	-	0	0.97	0.91	92.96	56.19	-	-	
Maximum	91.13	3.15	68.77	65.26	1.77	8.95	31.3	12.58	-	-	0	0.97	0.91	92.96	56.19	-	-	
Standard deviation	0	0	0	0	0	0	0	0	-	-	0	0	0	0	0	-	-	
CV (%)	0	0	0	0	0	0	0	0	-	-	0	0	0	0	0	-	-	
Peanut hulls	92.36	14.14	88.81	51.03	20.42	12.98	11.19	20.07	-	-	15.93	0.63	0.51	67.27	46.54	-	-	Montcho et al., 2016
Average	92.36	14.14	88.81	51.03	20.42	12.98	11.19	20.07	-	-	15.93	0.63	0.51	67.27	46.54	-	-	Montcho et al., 2016 (n=1)
Minimum	92.36	14.14	88.81	51.03	20.42	12.98	11.19	20.07	-	-	15.93	0.63	0.51	67.27	46.54	-	-	
Maximum	92.36	14.14	88.81	51.03	20.42	12.98	11.19	20.07	-	-	15.93	0.63	0.51	67.27	46.54	-	-	
Standard deviation	0	0	0	0	0	0	0	0	-	-	0	0	0	0	0	-	-	
CV (%)	0	0	0	0	0	0	0	0	-	-	0	0	0	0	0	-	-	
Pineapple peels	91.39	1.44	89.94	66.54	6.39	12.17	10.1	16.55	-	-	3.14	0.9	0.87	62.64	54.96	-	-	Montcho et al., 2016
Average	91.39	1.44	89.94	66.54	6.39	12.17	10.1	16.55	-	-	3.14	0.9	0.87	62.64	54.96	-	-	Montcho et al., 2016 (n=1)
Minimum	91.39	1.44	89.94	66.54	6.39	12.17	10.1	16.55	-	-	3.14	0.9	0.87	62.64	54.96	-	-	
Maximum	91.39	1.44	89.94	66.54	6.39	12.17	10.1	16.55	-	-	3.14	0.9	0.87	62.64	54.96	-	-	
Standard deviation	0	0	0	0	0	0	0	0	-	-	0	0	0	0	0	-	-	
CV (%)	0	0	0	0	0	0	0	0	-	-	0	0	0	0	0	-	-	
Rice polishings	90.1	0.29	92.57	70.4	10.72	1.68	7.49	16.69	-	-	7.06	0.97	0.88	48.05	62.04	-	-	Montcho et al., 2016
Average	90.1	0.29	92.57	70.4	10.72	1.68	7.49	16.69	-	-	7.06	0.97	0.88	48.05	62.04	-	-	Montcho et al., 2016 (n=1)
Minimum	90.1	0.29	92.57	70.4	10.72	1.68	7.49	16.69	-	-	7.06	0.97	0.88	48.05	62.04	-	-	
Maximum	90.1	0.29	92.57	70.4	10.72	1.68	7.49	16.69	-	-	7.06	0.97	0.88	48.05	62.04	-	-	
Standard deviation	0	0	0	0	0	0	0	0	-	-	0	0	0	0	0	-	-	
CV (%)	0	0	0	0	0	0	0	0	-	-	0	0	0	0	0	-	-	
Rice bran	91.49	12.8	88.55	-	17.14	10.11	11.52	-	-	-	-	-	-	45.51	29.1	-	-	Montcho et al., 2016
Average	91.49	12.8	88.55	-	17.14	10.11	11.52	-	-	-	-	-	-	45.51	29.1	-	-	Montcho et al., 2016 (n=1)
Minimum	91.49	12.8	88.55	-	17.14	10.11	11.52	-	-	-	-	-	-	45.51	29.1	-	-	
Maximum	91.49	12.8	88.55	-	17.14	10.11	11.52	-	-	-	-	-	-	45.51	29.1	-	-	
Standard deviation	0	0	0	-	0	0	0	-	-	-	-	-	-	0	0	-	-	
CV (%)	0	0	0	-	0	0	0	-	-	-	-	-	-	0	0	-	-	
Shea cake	95.16	14.26	92.38	-	27.43	11.17	7.78	-	-	-	-	-	-	68.86	60.23	-	-	Montcho et al., 2016
Average	95.16	14.26	92.38	-	27.43	11.17	7.78	-	-	-	-	-	-	68.86	60.23	-	-	Montcho et al., 2016 (n=1)
Minimum	95.16	14.26	92.38	-	27.43	11.17	7.78	-	-	-	-	-	-	68.86	60.23	-	-	
Maximum	95.16	14.26	92.38	-	27.43	11.17	7.78	-	-	-	-	-	-	68.86	60.23	-	-	
Standard deviation	0	0	0	-	0	0	0	-	-	-	-	-	-	0	0	-	-	
CV (%)	0	0	0	-	0	0	0	-	-	-	-	-	-	0	0	-	-	
Sorghum bran	90.7	-	-	-	10.5	7.9	-	-	-	-	68	0.78	-	-	-	0.09	0.64	Sanogo et al., 2019
Average	90.7	-	-	-	10.5	7.9	-	-	-	-	68	0.78	-	-	-	0.09	0.64	Sanogo et al., 2019 (n=1)
Minimum	90.7	-	-	-	10.5	7.9	-	-	-	-	68	0.78	-	-	-	0.09	0.64	
Maximum	90.7	-	-	-	10.5	7.9	-	-	-	-	68	0.78	-	-	-	0.09	0.64	
Standard deviation	0	-	-	-	0	0	-	-	-	-	0	0	-	-	-	0	0	
CV (%)	0	-	-	-	0	0	-	-	-	-	0	0	-	-	-	0	0	
Sorghum spent grains	96.16	-	76.25	-	19.54	-	23.75	-	-	-	123.96	0.15	0.2	-	-	-	-	Gbenou et al., 2020
Sorghum spent grains	93.98	10.13	81.87	76.68	19.5	15.15	13.7	19.92	-	-	15.13	1.03	0.96	64.74	50.9	-	-	Montcho et al., 2016
Average	95.07	10.13	79.06	76.68	19.52	15.15	18.73	19.92	-	-	69.55	0.59	0.58	64.74	50.9	-	-	Gbenou et al., 2020; Montcho et al., 2016 (n=2)

Minimum	93.98	10.13	76.25	76.68	19.5	15.15	13.7	19.92	-	-	15.13	0.15	0.2	64.74	50.9	-	-	
Maximum	96.16	10.13	81.87	76.68	19.54	15.15	23.75	19.92	-	-	123.96	1.03	0.96	64.74	50.9	-	-	
Standard deviation	1.54	0	3.97	0	0.03	0	7.11	0	-	-	76.95	0.62	0.54	0	0	-	-	
CV (%)	1.62	0	5.03	0	0.14	0	37.95	0	-	-	110.65	105.47	92.66	0	0	-	-	
Soybean cake	93.29	20.72	92.22	-	31.51	17.68	7.85	-	-	-	-	-	-	73.58	46.13	-	-	Montcho et al., 2016
Average	93.29	20.72	92.22	-	31.51	17.68	7.85	-	-	-	-	-	-	73.58	46.13	-	-	Montcho et al., 2016 (n=1)
Minimum	93.29	20.72	92.22	-	31.51	17.68	7.85	-	-	-	-	-	-	73.58	46.13	-	-	
Maximum	93.29	20.72	92.22	-	31.51	17.68	7.85	-	-	-	-	-	-	73.58	46.13	-	-	
Standard deviation	0	0	0	-	0	0	0	-	-	-	-	-	-	0	0	-	-	
CV (%)	0	0	0	-	0	0	0	-	-	-	-	-	-	0	0	-	-	
Wheat bran	94.6	-	94.7	-	16.38	9.6	10.9	-	-	-	-	-	-	48.2	14	1.07	0.97	Abdou et al., 2011
Wheat bran	96	3.77	93.9	-	18	11.6	6.1	-	-	-	-	-	-	-	-	-	-	Mahamadou Abdou Malam et al., 2021
Wheat bran	89.88	3.04	-	-	12.38	17.96	11.92	12.9704	-	-	-	-	-	64.28	43.37	-	-	Lamidi and Ogunkunle, 2015
Wheat bran	94.5	4.42	-	-	16.16	7.26	4.26	-	-	-	-	-	-	35.94	10.19	-	-	Kondombo and Niannongo, 2001
Average	93.75	3.74	94.3	-	15.73	11.61	8.29	12.97	-	-	-	-	-	49.47	22.52	1.07	0.97	Abdou et al., 2011; Mahamadou Abdou Malam et al., 2021; Lamidi and Ogunkunle, 2015; Kondombo and Niannongo, 2001 (n=4)
Minimum	89.88	3.04	93.9	-	12.38	7.26	4.26	12.97	-	-	-	-	-	35.94	10.19	1.07	0.97	
Maximum	96	4.42	94.7	-	18	17.96	11.92	12.97	-	-	-	-	-	64.28	43.37	1.07	0.97	
Standard deviation	2.67	0.69	0.57	-	2.38	4.59	3.7	0	-	-	-	-	-	14.21	18.16	0	0	
CV (%)	2.84	18.44	0.6	-	15.13	39.58	44.58	0	-	-	-	-	-	28.73	80.63	0	0	
Yam peels	88.99	0.32	93.97	77.11	7.99	4.44	5.5	17	-	-	-	1.07	1.07	75.31	66.19	-	-	Montcho et al., 2016
Average	88.99	0.32	93.97	77.11	7.99	4.44	5.5	17	-	-	-	1.07	1.07	75.31	66.19	-	-	Montcho et al., 2016 (n=1)
Minimum	88.99	0.32	93.97	77.11	7.99	4.44	5.5	17	-	-	-	1.07	1.07	75.31	66.19	-	-	
Maximum	88.99	0.32	93.97	77.11	7.99	4.44	5.5	17	-	-	-	1.07	1.07	75.31	66.19	-	-	
Standard deviation	0	0	0	0	0	0	0	0	-	-	-	0	0	0	0	-	-	
CV (%)	0	0	0	0	0	0	0	0	-	-	-	0	0	0	0	-	-	
Cottonseed meal	85.6	-	94.6	-	24.13	-	5.4	-	-	-	-	-	-	70.9	-	-	-	Etela et al., 2008
Average	85.6	-	94.6	-	24.13	-	5.4	-	-	-	-	-	-	70.9	-	-	-	Etela et al., 2008 (n=1)
Minimum	85.6	-	94.6	-	24.13	-	5.4	-	-	-	-	-	-	70.9	-	-	-	
Maximum	85.6	-	94.6	-	24.13	-	5.4	-	-	-	-	-	-	70.9	-	-	-	
Standard deviation	0	-	0	-	0	-	0	-	-	-	-	-	-	0	-	-	-	
CV (%)	0	-	0	-	0	-	0	-	-	-	-	-	-	0	-	-	-	

Table S4. Table of chemical composition of agricultural by-products for ruminants in West-Africa

Feed	DM (%)	EE (% DM)	OM (% DM)	dOM (% DM)	CP (% DM)	CF (% DM)	Ash (% DM)	GE (MJ/kg DM)	DE (MJ/kg DM)	ME (MJ/kg DM)	DCP (g/kg DM)	UFL	UFV	NDF (% DM)	ADF (% DM)	Ca (% DM)	P (% DM)	References
Cowpea haulms	92.01	2.15	88.46	-	13.48	30.5	11.66	-	-	-	-	-	-	71.44	56.28	-	-	Montcho et al., 2016
Cowpea haulms	93.9	-	84.28	-	6.11	24.93	9.62	-	-	-	2.15	-	-	44.09	24.88	-	-	Kiema et al., 2019
Cowpea haulms																		
Cowpea haulms	94.8	-	-	-	18.1	19.4	9.43	-	-	-	-	-	-	56.9	39.9	-	-	Anele et al., 2012
Cowpea haulms	-	-	-	55.5	10	-	12	-	-	7.68	-	-	-	45.6	72.6	-	-	Amole et al., 2022
Cowpea haulms	97.26	8.33	-	-	8.31	50.12	7.61	-	-	-	-	-	0.41	69.04	53.44	-	-	Kondombo and Niannongo, 2001
Cowpea haulms	92.2	-	-	-	13.2	23	-	-	-	-	8.6	-	-	-	-	-	-	Sanogo et al., 2019
Average	94.03	5.24	86.37	55.5	11.53	29.59	10.06	-	-	7.68	5.38	-	0.41	57.41	49.42	-	-	Montcho et al., 2016; Kiema et al., 2019; Anele et al., 2012; Amole et al., 2022; Kondombo and Niannongo, 2001; Sanogo et al., 2019 (n=6)
Minimum	92.01	2.15	84.28	55.5	6.11	19.4	7.61	-	-	7.68	2.15	-	0.41	44.09	24.88	-	-	
Maximum	97.26	8.33	88.46	55.5	18.1	50.12	12	-	-	7.68	8.6	-	0.41	71.44	72.6	-	-	
Standard deviation	2.15	4.37	2.96	0	4.29	12.16	1.8	-	-	0	4.56	-	0	12.74	17.98	-	-	
CV (%)	2.29	83.4	3.42	0	37.16	41.09	17.86	-	-	0	84.85	-	0	22.19	36.39	-	-	
Groundnut haulms	93.7	-	92.4	-	9.44	-	-	-	-	-	-	-	-	56.6	42.2	0.87	0.07	Abdou et al., 2011
Groundnut haulms	86.92	10.45	93.46	-	15.48	22.09	6.57	-	-	-	-	-	-	65.63	43.52	-	-	Montcho et al., 2016
Groundnut haulms	86.2	-	89.7	-	11.1	37.5	10.3	-	-	-	5.76	0.76	-	-	-	0.74	0.12	Nantoumé et al., 2000
Average	88.94	10.45	91.85	-	12.01	29.8	8.44	-	-	-	5.76	0.76	-	61.11	42.86	0.8	0.1	Abdou et al., 2011; Montcho et al., 2016; Nantoumé et al., 2000 (n=3)
Minimum	86.2	10.45	89.7	-	9.44	22.09	6.57	-	-	-	5.76	0.76	-	56.6	42.2	0.74	0.07	
Maximum	93.7	10.45	93.46	-	15.48	37.5	10.3	-	-	-	5.76	0.76	-	65.63	43.52	0.87	0.12	
Standard deviation	4.14	0	1.94	-	3.12	10.9	2.64	-	-	-	0	0	-	6.39	0.93	0.09	0.04	
CV (%)	4.65	0	2.11	-	25.99	36.57	31.27	-	-	-	0	0	-	10.45	2.18	11.42	37.22	
Groundnut haulms (Azivivi)	84.9	-	89.1	-	11.2	-	10.9	-	-	-	-	-	-	42.5	39	-	-	Ansah et al., 2017
Average	84.9	-	89.1	-	11.2	-	10.9	-	-	-	-	-	-	42.5	39	-	-	Ansah et al., 2017 (n=1)
Minimum	84.9	-	89.1	-	11.2	-	10.9	-	-	-	-	-	-	42.5	39	-	-	
Maximum	84.9	-	89.1	-	11.2	-	10.9	-	-	-	-	-	-	42.5	39	-	-	
Standard deviation	0	-	0	-	0	-	0	-	-	-	-	-	-	0	0	-	-	
CV (%)	0	-	0	-	0	-	0	-	-	-	-	-	-	0	0	-	-	
Groundnut haulms (Manipinta)	87.2	-	91.9	-	10.3	-	8.1	-	-	-	-	-	-	44.9	36.7	-	-	Ansah et al., 2017
Average	87.2	-	91.9	-	10.3	-	8.1	-	-	-	-	-	-	44.9	36.7	-	-	Ansah et al., 2017 (n=1)
Minimum	87.2	-	91.9	-	10.3	-	8.1	-	-	-	-	-	-	44.9	36.7	-	-	
Maximum	87.2	-	91.9	-	10.3	-	8.1	-	-	-	-	-	-	44.9	36.7	-	-	
Standard deviation	0	-	0	-	0	-	0	-	-	-	-	-	-	0	0	-	-	
CV (%)	0	-	0	-	0	-	0	-	-	-	-	-	-	0	0	-	-	
Groundnut haulms (Obolo)	84.7	-	89.9	-	6.8	-	10.1	-	-	-	-	-	-	53	49.5	-	-	Ansah et al., 2017
Average	84.7	-	89.9	-	6.8	-	10.1	-	-	-	-	-	-	53	49.5	-	-	Ansah et al., 2017 (n=1)
Minimum	84.7	-	89.9	-	6.8	-	10.1	-	-	-	-	-	-	53	49.5	-	-	

Maximum	84.7	-	89.9	-	6.8	-	10.1	-	-	-	-	-	-	53	49.5	-	-	
Standard deviation	0	-	0	-	0	-	0	-	-	-	-	-	-	0	0	-	-	
CV (%)	0	-	0	-	0	-	0	-	-	-	-	-	-	0	0	-	-	
Groundnut haulms (Yenyawoso)	88.2	-	90.3	-	11.4	-	9.7	-	-	-	-	-	-	42.7	36.6	-	-	Ansah et al., 2017
Average	88.2	-	90.3	-	11.4	-	9.7	-	-	-	-	-	-	42.7	36.6	-	-	Ansah et al., 2017 (n=1)
Minimum	88.2	-	90.3	-	11.4	-	9.7	-	-	-	-	-	-	42.7	36.6	-	-	
Maximum	88.2	-	90.3	-	11.4	-	9.7	-	-	-	-	-	-	42.7	36.6	-	-	
Standard deviation	0	-	0	-	0	-	0	-	-	-	-	-	-	0	0	-	-	
CV (%)	0	-	0	-	0	-	0	-	-	-	-	-	-	0	0	-	-	
Faidherbia albida pods	92.57	-	88.55	-	6.1	28.04	4.02	-	-	-	21.44	-	-	40.58	29.92	-	-	Kiéma et al., 2019
Average	92.57	-	88.55	-	6.1	28.04	4.02	-	-	-	21.44	-	-	40.58	29.92	-	-	Kiéma et al., 2019 (n=1)
Minimum	92.57	-	88.55	-	6.1	28.04	4.02	-	-	-	21.44	-	-	40.58	29.92	-	-	
Maximum	92.57	-	88.55	-	6.1	28.04	4.02	-	-	-	21.44	-	-	40.58	29.92	-	-	
Standard deviation	0	-	0	-	0	0	0	-	-	-	0	-	-	0	0	-	-	
CV (%)	0	-	0	-	0	0	0	-	-	-	0	-	-	0	0	-	-	
Lablab haulms	86.3	-	87.3	-	11.9	36.7	12.7	-	-	-	4.97	0.63	-	-	-	0.85	0.19	Nantoumé et al., 2000
Average	86.3	-	87.3	-	11.9	36.7	12.7	-	-	-	4.97	0.63	-	-	-	0.85	0.19	Nantoumé et al., 2000 (n=1)
Minimum	86.3	-	87.3	-	11.9	36.7	12.7	-	-	-	4.97	0.63	-	-	-	0.85	0.19	
Maximum	86.3	-	87.3	-	11.9	36.7	12.7	-	-	-	4.97	0.63	-	-	-	0.85	0.19	
Standard deviation	0	-	0	-	0	0	0	-	-	-	0	0	-	-	-	0	0	
CV (%)	0	-	0	-	0	0	0	-	-	-	0	0	-	-	-	0	0	
Maize straw	89.9	-	92	-	2.2	41.8	8	-	-	-	0	0.5	-	-	-	-	-	Nantoumé et al., 2000
Maize straw	93.31	-	83.93	-	4.37	37.23	9.38	-	-	-	0.54	-	-	68.63	35.98	-	-	Kiema et al., 2019
Average	91.61	-	87.97	-	3.29	39.52	8.69	-	-	-	0.27	0.5	-	68.63	35.98	-	-	Nantoumé et al., 2000; Kiema et al., 2019 (n=2)
Minimum	89.9	-	83.93	-	2.2	37.23	8	-	-	-	0	0.5	-	68.63	35.98	-	-	
Maximum	93.31	-	92	-	4.37	41.8	9.38	-	-	-	0.54	0.5	-	68.63	35.98	-	-	
Standard deviation	2.41	-	5.71	-	1.53	3.23	0.98	-	-	-	0.38	0	-	0	0	-	-	
CV (%)	2.63	-	6.49	-	46.71	8.18	11.23	-	-	-	141.42	0	-	0	0	-	-	
Millet stover	93.8	-	93.5	-	4.62	-	-	-	-	-	-	-	-	85.9	53.2	0.2	0.04	Abdou et al., 2011
Millet stover	-	-	-	35	14.9	-	7.5	-	-	5.24	-	-	-	64.1	71.1	-	-	Amole et al., 2022
Millet stover	85	-	-	-	5.6	41.4	-	-	-	-	1.9	-	-	-	-	-	-	Sanogo et al., 2019
Average	89.4	-	93.5	35	8.37	41.4	7.5	-	-	5.24	1.9	-	-	75	62.15	0.2	0.04	Abdou et al., 2011; Amole et al., 2022; Sanogo et al., 2019 (n=3)
Minimum	85	-	93.5	35	4.62	41.4	7.5	-	-	5.24	1.9	-	-	64.1	53.2	0.2	0.04	
Maximum	93.8	-	93.5	35	14.9	41.4	7.5	-	-	5.24	1.9	-	-	85.9	71.1	0.2	0.04	
Standard deviation	6.22	-	0	0	5.67	0	0	-	-	0	0	-	-	15.41	12.66	0	0	
CV (%)	6.96	-	0	0	67.76	0	0	-	-	0	0	-	-	20.55	20.37	0	0	
Millet straw	89.1	-	84.3	-	3.9	45.8	15.7	-	-	-	0	0.42	-	-	-	-	-	Nantoumé et al., 2000
Average	89.1	-	84.3	-	3.9	45.8	15.7	-	-	-	0	0.42	-	-	-	-	-	Nantoumé et al., 2000 (n=1)
Minimum	89.1	-	84.3	-	3.9	45.8	15.7	-	-	-	0	0.42	-	-	-	-	-	
Maximum	89.1	-	84.3	-	3.9	45.8	15.7	-	-	-	0	0.42	-	-	-	-	-	
Standard deviation	0	-	0	-	0	0	0	-	-	-	0	0	-	-	-	-	-	

CV (%)	0	-	0	-	0	0	0	-	-	-	0	0	-	-	-	-	-	
Néré pods	85.81	1.11	94.26	-	0.32	21.77	5.8	-	-	-	-	-	-	72.34	59.32	-	-	Montcho et al., 2016
Average	85.81	1.11	94.26	-	0.32	21.77	5.8	-	-	-	-	-	-	72.34	59.32	-	-	Montcho et al., 2016 (n=1)
Minimum	85.81	1.11	94.26	-	0.32	21.77	5.8	-	-	-	-	-	-	72.34	59.32	-	-	
Maximum	85.81	1.11	94.26	-	0.32	21.77	5.8	-	-	-	-	-	-	72.34	59.32	-	-	
Standard deviation	0	0	0	-	0	0	0	-	-	-	-	-	-	0	0	-	-	
CV (%)	0	0	0	-	0	0	0	-	-	-	-	-	-	0	0	-	-	
Peanut haulms	93.7	-	92.4	-	-	25.9	10.31	-	-	-	-	-	-	56.6	42.2	1.1	1.5	Abdou et al., 2011
Peanut haulms	-	-	-	65.1	20.7	-	10.9	-	-	4.97	-	-	-	12.9	35.5	-	-	Amole et al., 2022
Peanut haulms	96.45	0.55	-	-	7.35	43.4	13.72	-	-	-	-	-	0.42	59.26	52.51	-	-	Kondombo and Niannongo, 2001
Peanut haulms	86.92	10.45	93.46	57.84	15.48	22.09	6.57	20.35	-	-	10.92	0.73	0.62	65.63	43.52	-	-	Montcho et al., 2016
Peanut haulms	90.7	-	-	-	20.1	22.7	-	-	-	-	15.6	-	-	-	-	-	-	Sanogo et al., 2019
Peanut haulms	94.5	2.11	-	-	17.32	29.75	8.12	16.35	-	-	-	-	-	-	-	-	-	Sidibé et al., 2019
Average	92.45	4.37	92.93	61.47	16.19	28.77	9.92	18.35	-	4.97	13.26	0.73	0.52	48.6	43.43	1.1	1.5	Abdou et al., 2011; Amole et al., 2022; Kondombo and Niannongo, 2001; Montcho et al., 2016; Sanogo et al., 2019; Sidibé et al., 2019 (n=6)
Minimum	86.92	0.55	92.4	57.84	7.35	22.09	6.57	16.35	-	4.97	10.92	0.73	0.42	12.9	35.5	1.1	1.5	
Maximum	96.45	10.45	93.46	65.1	20.7	43.4	13.72	20.35	-	4.97	15.6	0.73	0.62	65.63	52.51	1.1	1.5	
Standard deviation	3.72	5.32	0.75	5.13	5.37	8.73	2.74	2.83	-	0	3.31	0	0.14	24.1	7	0	0	
CV (%)	4.03	121.81	0.81	8.35	33.2	30.34	27.6	15.41	-	0	24.96	0	27.2	49.59	16.11	0	0	
Rice hulls	93.57	0.98	75.21	36.23	1.89	37.68	24.77	14.44	-	-	-	0.46	0.38	87.24	76.22	-	-	Montcho et al., 2016
Average	93.57	0.98	75.21	36.23	1.89	37.68	24.77	14.44	-	-	-	0.46	0.38	87.24	76.22	-	-	Montcho et al., 2016 (n=1)
Minimum	93.57	0.98	75.21	36.23	1.89	37.68	24.77	14.44	-	-	-	0.46	0.38	87.24	76.22	-	-	
Maximum	93.57	0.98	75.21	36.23	1.89	37.68	24.77	14.44	-	-	-	0.46	0.38	87.24	76.22	-	-	
Standard deviation	0	0	0	0	0	0	0	0	-	-	-	0	0	0	0	-	-	
CV (%)	0	0	0	0	0	0	0	0	-	-	-	0	0	0	0	-	-	
AGRA rice straw	89.33	0.79	87.1	31.29	7.09	28.28	12.9	-	-	3.35	-	-	-	58.99	42.67	-	-	Sasu et al., 2023
Average	89.33	0.79	87.1	31.29	7.09	28.28	12.9	-	-	3.35	-	-	-	58.99	42.67	-	-	Sasu et al., 2023 (n=1)
Minimum	89.33	0.79	87.1	31.29	7.09	28.28	12.9	-	-	3.35	-	-	-	58.99	42.67	-	-	
Maximum	89.33	0.79	87.1	31.29	7.09	28.28	12.9	-	-	3.35	-	-	-	58.99	42.67	-	-	
Standard deviation	0	0	0	0	0	0	0	-	-	0	-	-	-	0	0	-	-	
CV (%)	0	0	0	0	0	0	0	-	-	0	-	-	-	0	0	-	-	
AMANKWATIA rice straw	87.23	1.44	83.48	32.55	6.68	22.72	16.52	-	-	3.39	-	-	-	57.88	45.77	-	-	Sasu et al., 2023
Average	87.23	1.44	83.48	32.55	6.68	22.72	16.52	-	-	3.39	-	-	-	57.88	45.77	-	-	Sasu et al., 2023 (n=1)
Minimum	87.23	1.44	83.48	32.55	6.68	22.72	16.52	-	-	3.39	-	-	-	57.88	45.77	-	-	
Maximum	87.23	1.44	83.48	32.55	6.68	22.72	16.52	-	-	3.39	-	-	-	57.88	45.77	-	-	
Standard deviation	0	0	0	0	0	0	0	-	-	0	-	-	-	0	0	-	-	
CV (%)	0	0	0	0	0	0	0	-	-	0	-	-	-	0	0	-	-	
Rice straw	93.7	-	82.69	-	6.68	-	17.31	-	-	-	-	-	-	58.9	51.7	-	-	Idan et al., 2023
Rice straw	93.3	-	-	-	5.93	38	-	-	-	-	-	-	-	-	-	-	-	Sanogo et al., 2019
Rice straw	93	-	-	-	4.25	-	12.9	-	-	-	-	-	-	61.7	-	-	-	Sanon et al., 2013

Average	93.33	-	82.69	-	5.62	38	15.11	-	-	-	-	-	-	60.3	51.7	-	-	Idan et al., 2023; Sanogo et al., 2019; Sanon et al., 2013 (n=3)
Minimum	93	-	82.69	-	4.25	38	12.9	-	-	-	-	-	-	58.9	51.7	-	-	
Maximum	93.7	-	82.69	-	6.68	38	17.31	-	-	-	-	-	-	61.7	51.7	-	-	
Standard deviation	0.35	-	0	-	1.24	0	3.12	-	-	-	-	-	-	1.98	0	-	-	
CV (%)	0.38	-	0	-	22.14	0	20.64	-	-	-	-	-	-	3.28	0	-	-	
Sorghum hulls	92.61	2.98	95.65	31.96	4.05	29.19	4.49	18.51	-	-	-	0.38	0.26	87.33	82.66	-	-	Montcho et al., 2016
Average	92.61	2.98	95.65	31.96	4.05	29.19	4.49	18.51	-	-	-	0.38	0.26	87.33	82.66	-	-	Montcho et al., 2016 (n=1)
Minimum	92.61	2.98	95.65	31.96	4.05	29.19	4.49	18.51	-	-	-	0.38	0.26	87.33	82.66	-	-	
Maximum	92.61	2.98	95.65	31.96	4.05	29.19	4.49	18.51	-	-	-	0.38	0.26	87.33	82.66	-	-	
Standard deviation	0	0	0	0	0	0	0	0	-	-	-	0	0	0	0	-	-	
CV (%)	0	0	0	0	0	0	0	0	-	-	-	0	0	0	0	-	-	
Sorghum stover	94.53	0.64	93.69	-	3.29	45.18	6.56	-	-	-	-	-	-	91.59	89.88	-	-	Montcho et al., 2016
Sorghum stover	-	-	-	46.7	4	-	10.7	-	-	6.9	-	-	-	73.6	48.2	-	-	Amole et al., 2022
Sorghum stover	85	-	-	-	3.8	38.6	-	-	-	-	1.4	-	-	-	-	-	-	Sanogo et al., 2019
Sorghum stover	96	1.51	76	-	8.2	32.6	24	-	-	-	-	-	-	-	-	-	-	Mahamadou Abdou Malam et al., 2021
Average	91.84	1.07	84.84	46.7	4.82	38.79	13.75	-	-	6.9	1.4	-	-	82.59	69.04	-	-	Montcho et al., 2016; Amole et al., 2022; Sanogo et al., 2019; Mahamadou Abdou Malam et al., 2021 (n=4)
Minimum	85	0.64	76	46.7	3.29	32.6	6.56	-	-	6.9	1.4	-	-	73.6	48.2	-	-	
Maximum	96	1.51	93.69	46.7	8.2	45.18	24	-	-	6.9	1.4	-	-	91.59	89.88	-	-	
Standard deviation	5.97	0.62	12.51	0	2.27	6.29	9.11	-	-	0	0	-	-	12.72	29.47	-	-	
CV (%)	6.5	57.23	14.74	0	47.1	16.22	66.25	-	-	0	0	-	-	15.4	42.69	-	-	
Sorghum stems	96	1.51	76	-	8.2	32.6	24	-	-	-	-	-	-	-	-	-	-	Mahamadou Abdou Malam et al., 2021
Average	96	1.51	76	-	8.2	32.6	24	-	-	-	-	-	-	-	-	-	-	Mahamadou Abdou Malam et al., 2021 (n=1)
Minimum	96	1.51	76	-	8.2	32.6	24	-	-	-	-	-	-	-	-	-	-	
Maximum	96	1.51	76	-	8.2	32.6	24	-	-	-	-	-	-	-	-	-	-	
Standard deviation	0	0	0	-	0	0	0	-	-	-	-	-	-	-	-	-	-	
CV (%)	0	0	0	-	0	0	0	-	-	-	-	-	-	-	-	-	-	
Sorghum straw	97.51	1.52	-	-	3	42.17	6.92	-	-	-	-	-	0.3	78.02	45.27	-	-	Kondombo and Niannongo, 2001
Sorghum straw	91.9	-	89.7	-	3.3	45.5	10.3	-	-	-	0.1	0.6	-	72.2	59.3	-	-	Nantoumé et al., 2000
Average	94.71	1.52	89.7	-	3.15	43.84	8.61	-	-	-	0.1	0.6	0.3	75.11	52.28	-	-	Kondombo and Niannongo, 2001; Nantoumé et al., 2000 (n=2)
Minimum	91.9	1.52	89.7	-	3	42.17	6.92	-	-	-	0.1	0.6	0.3	72.2	45.27	-	-	
Maximum	97.51	1.52	89.7	-	3.3	45.5	10.3	-	-	-	0.1	0.6	0.3	78.02	59.3	-	-	
Standard deviation	3.97	0	0	-	0.21	2.35	2.39	-	-	-	0	0	0	4.12	9.92	-	-	
CV (%)	4.19	0	0	-	6.73	5.37	27.76	-	-	-	0	0	0	5.48	18.97	-	-	
Soybean pods	93.66	0.65	94.77	-	5.9	49.11	5.6	-	-	-	-	-	-	89.12	77.31	-	-	Montcho et al., 2016
Average	93.66	0.65	94.77	-	5.9	49.11	5.6	-	-	-	-	-	-	89.12	77.31	-	-	Montcho et al., 2016 (n=1)
Minimum	93.66	0.65	94.77	-	5.9	49.11	5.6	-	-	-	-	-	-	89.12	77.31	-	-	
Maximum	93.66	0.65	94.77	-	5.9	49.11	5.6	-	-	-	-	-	-	89.12	77.31	-	-	
Standard deviation	0	0	0	-	0	0	0	-	-	-	-	-	-	0	0	-	-	

CV (%)	0	0	0	-	0	0	0	-	-	-	-	-	-	0	0	-	-	
Sweet potato peels	88.8	0.87	92.82	81.31	8.04	5.51	7.56	16.83	-	-	4.62	1.16	0.99	53.59	43.2	-	-	Montcho et al., 2016
Average	88.8	0.87	92.82	81.31	8.04	5.51	7.56	16.83	-	-	4.62	1.16	0.99	53.59	43.2	-	-	Montcho et al., 2016 (n=1)
Minimum	88.8	0.87	92.82	81.31	8.04	5.51	7.56	16.83	-	-	4.62	1.16	0.99	53.59	43.2	-	-	
Maximum	88.8	0.87	92.82	81.31	8.04	5.51	7.56	16.83	-	-	4.62	1.16	0.99	53.59	43.2	-	-	
Standard deviation	0	0	0	0	0	0	0	0	-	-	0	0	0	0	0	-	-	
CV (%)	0	0	0	0	0	0	0	0	-	-	0	0	0	0	0	-	-	
Yam peels	88.99	0.32	93.97	77.11	7.99	4.44	5.5	17	-	-	4.58	1.07	1.07	75.31	66.19	-	-	Montcho et al., 2016
Average	88.99	0.32	93.97	77.11	7.99	4.44	5.5	17	-	-	4.58	1.07	1.07	75.31	66.19	-	-	Montcho et al., 2016 (n=1)
Minimum	88.99	0.32	93.97	77.11	7.99	4.44	5.5	17	-	-	4.58	1.07	1.07	75.31	66.19	-	-	
Maximum	88.99	0.32	93.97	77.11	7.99	4.44	5.5	17	-	-	4.58	1.07	1.07	75.31	66.19	-	-	
Standard deviation	0	0	0	0	0	0	0	0	-	-	0	0	0	0	0	-	-	
CV (%)	0	0	0	0	0	0	0	0	-	-	0	0	0	0	0	-	-	
Threshed sorghum top	-	1.77	93.54	-	6.68	-	6.46	-	-	-	-	-	-	65.42	34.81	0.06	0.04	Isah et al., 2015
Average	-	1.77	93.54	-	6.68	-	6.46	-	-	-	-	-	-	65.42	34.81	0.06	0.04	Isah et al., 2015 (n=1)
Minimum	-	1.77	93.54	-	6.68	-	6.46	-	-	-	-	-	-	65.42	34.81	0.06	0.04	
Maximum	-	1.77	93.54	-	6.68	-	6.46	-	-	-	-	-	-	65.42	34.81	0.06	0.04	
Standard deviation	-	0	0	-	0	-	0	-	-	-	-	-	-	0	0	0	0	
CV (%)	-	0	0	-	0	-	0	-	-	-	-	-	-	0	0	0	0	
Maize stover	30.43	1.3	93.17	-	7.03	31.28	6.83	-	-	-	-	-	-	68.57	38.8	-	-	Ogunbosoye and Odedire, 2022
Average	30.43	1.3	93.17	-	7.03	31.28	6.83	-	-	-	-	-	-	68.57	38.8	-	-	Ogunbosoye and Odedire, 2022 (n=1)
Minimum	30.43	1.3	93.17	-	7.03	31.28	6.83	-	-	-	-	-	-	68.57	38.8	-	-	
Maximum	30.43	1.3	93.17	-	7.03	31.28	6.83	-	-	-	-	-	-	68.57	38.8	-	-	
Standard deviation	0	0	0	-	0	0	0	-	-	-	-	-	-	0	0	-	-	
CV (%)	0	0	0	-	0	0	0	-	-	-	-	-	-	0	0	-	-	
Maize husk	29.15	0.81	97.02	-	4.5	31.31	2.98	-	-	-	-	-	-	63.74	41.85	-	-	Ogunbosoye and Odedire, 2022
Maize husk	-	-	-	57.9	1.9	-	5.9	-	-	8.85	-	-	-	87.1	43.1	4.7	3.4	Amole et al., 2022
Average	29.15	0.81	97.02	57.9	3.2	31.31	4.44	-	-	8.85	-	-	-	75.42	42.48	4.7	3.4	Ogunbosoye and Odedire, 2022; Amole et al., 2022 (n=2)
Minimum	29.15	0.81	97.02	57.9	1.9	31.31	2.98	-	-	8.85	-	-	-	63.74	41.85	4.7	3.4	
Maximum	29.15	0.81	97.02	57.9	4.5	31.31	5.9	-	-	8.85	-	-	-	87.1	43.1	4.7	3.4	
Standard deviation	0	0	0	0	1.84	0	2.06	-	-	0	-	-	-	16.52	0.88	0	0	
CV (%)	0	0	0	0	57.45	0	46.5	-	-	0	-	-	-	21.9	2.08	0	0	
Pearl millet stover [Somno]	93.2	-	91.8	50	5.62	-	8.2	-	-	7.4	-	-	-	73.8	44.1	-	-	Umutoni et al., 2021
Average	93.2	-	91.8	50	5.62	-	8.2	-	-	7.4	-	-	-	73.8	44.1	-	-	Umutoni et al., 2021 (n=1)
Minimum	93.2	-	91.8	50	5.62	-	8.2	-	-	7.4	-	-	-	73.8	44.1	-	-	
Maximum	93.2	-	91.8	50	5.62	-	8.2	-	-	7.4	-	-	-	73.8	44.1	-	-	
Standard deviation	0	-	0	0	0	-	0	-	-	0	-	-	-	0	0	-	-	
CV (%)	0	-	0	0	0	-	0	-	-	0	-	-	-	0	0	-	-	
Pearl millet stover [Chakti]	93.1	-	93.5	49	5	-	6.5	-	-	7.3	-	-	-	75.7	46.3	-	-	Umutoni et al., 2021
Average	93.1	-	93.5	49	5	-	6.5	-	-	7.3	-	-	-	75.7	46.3	-	-	Umutoni et al., 2021 (n=1)

Minimum	93.1	-	93.5	49	5	-	6.5	-	-	7.3	-	-	-	75.7	46.3	-	-	
Maximum	93.1	-	93.5	49	5	-	6.5	-	-	7.3	-	-	-	75.7	46.3	-	-	
Standard deviation	0	-	0	0	0	-	0	-	-	0	-	-	-	0	0	-	-	
CV (%)	0	-	0	0	0	-	0	-	-	0	-	-	-	0	0	-	-	
Pearl millet stover [ICMV167005]	92.5	-	91.6	51.3	6.25	-	8.4	-	-	7.6	-	-	-	68.8	42.1	-	-	Umutoni et al., 2021
Average	92.5	-	91.6	51.3	6.25	-	8.4	-	-	7.6	-	-	-	68.8	42.1	-	-	Umutoni et al., 2021 (n=1)
Minimum	92.5	-	91.6	51.3	6.25	-	8.4	-	-	7.6	-	-	-	68.8	42.1	-	-	
Maximum	92.5	-	91.6	51.3	6.25	-	8.4	-	-	7.6	-	-	-	68.8	42.1	-	-	
Standard deviation	0	-	0	0	0	-	0	-	-	0	-	-	-	0	0	-	-	
CV (%)	0	-	0	0	0	-	0	-	-	0	-	-	-	0	0	-	-	
Pearl millet stover [ICMV167006]	92.6	-	88.7	48.1	4.38	-	11.3	-	-	7.1	-	-	-	66.7	46.4	-	-	Umutoni et al., 2021
Average	92.6	-	88.7	48.1	4.38	-	11.3	-	-	7.1	-	-	-	66.7	46.4	-	-	Umutoni et al., 2021 (n=1)
Minimum	92.6	-	88.7	48.1	4.38	-	11.3	-	-	7.1	-	-	-	66.7	46.4	-	-	
Maximum	92.6	-	88.7	48.1	4.38	-	11.3	-	-	7.1	-	-	-	66.7	46.4	-	-	
Standard deviation	0	-	0	0	0	-	0	-	-	0	-	-	-	0	0	-	-	
CV (%)	0	-	0	0	0	-	0	-	-	0	-	-	-	0	0	-	-	
Pearl millet stover [ICMV167002]	92.6	-	91.2	51.8	6.25	-	8.8	-	-	7.7	-	-	-	67.6	40.9	-	-	Umutoni et al., 2021
Average	92.6	-	91.2	51.8	6.25	-	8.8	-	-	7.7	-	-	-	67.6	40.9	-	-	Umutoni et al., 2021 (n=1)
Minimum	92.6	-	91.2	51.8	6.25	-	8.8	-	-	7.7	-	-	-	67.6	40.9	-	-	
Maximum	92.6	-	91.2	51.8	6.25	-	8.8	-	-	7.7	-	-	-	67.6	40.9	-	-	
Standard deviation	0	-	0	0	0	-	0	-	-	0	-	-	-	0	0	-	-	
CV (%)	0	-	0	0	0	-	0	-	-	0	-	-	-	0	0	-	-	
Cassia tora leaves	94.4	2.81	-	-	18.81	29.34	17	15.68	-	-	-	-	-	-	-	-	-	Sidibé et al., 2019
Average	94.4	2.81	-	-	18.81	29.34	17	15.68	-	-	-	-	-	-	-	-	-	Sidibé et al., 2019 (n=1)
Minimum	94.4	2.81	-	-	18.81	29.34	17	15.68	-	-	-	-	-	-	-	-	-	
Maximum	94.4	2.81	-	-	18.81	29.34	17	15.68	-	-	-	-	-	-	-	-	-	
Standard deviation	0	0	-	-	0	0	0	0	-	-	-	-	-	-	-	-	-	
CV (%)	0	0	-	-	0	0	0	0	-	-	-	-	-	-	-	-	-	
Maize cobs	86.7	-	-	-	7.6	28.7	-	-	-	-	3	-	-	-	-	-	-	Sanogo et al., 2019
Average	86.7	-	-	-	7.6	28.7	-	-	-	-	3	-	-	-	-	-	-	Sanogo et al., 2019 (n=1)
Minimum	86.7	-	-	-	7.6	28.7	-	-	-	-	3	-	-	-	-	-	-	
Maximum	86.7	-	-	-	7.6	28.7	-	-	-	-	3	-	-	-	-	-	-	
Standard deviation	0	-	-	-	0	0	-	-	-	-	0	-	-	-	-	-	-	
CV (%)	0	-	-	-	0	0	-	-	-	-	0	-	-	-	-	-	-	
Corn stalks	-	-	-	55.6	4.4	-	5.3	-	-	7.87	-	-	-	67.2	42.5	3.2	0.8	Amole et al., 2022
Average	-	-	-	55.6	4.4	-	5.3	-	-	7.87	-	-	-	67.2	42.5	3.2	0.8	Amole et al., 2022 (n=1)
Minimum	-	-	-	55.6	4.4	-	5.3	-	-	7.87	-	-	-	67.2	42.5	3.2	0.8	
Maximum	-	-	-	55.6	4.4	-	5.3	-	-	7.87	-	-	-	67.2	42.5	3.2	0.8	
Standard deviation	-	-	-	0	0	-	0	-	-	0	-	-	-	0	0	0	0	
CV (%)	-	-	-	0	0	-	0	-	-	0	-	-	-	0	0	0	0	

Dried corn	87.3	-	-	-	10.6	2.4	-	-	-	-	6.6	-	-	-	-	-	-	Sanogo et al., 2019
Average	87.3	-	-	-	10.6	2.4	-	-	-	-	6.6	-	-	-	-	-	-	Sanogo et al., 2019 (n=1)
Minimum	87.3	-	-	-	10.6	2.4	-	-	-	-	6.6	-	-	-	-	-	-	
Maximum	87.3	-	-	-	10.6	2.4	-	-	-	-	6.6	-	-	-	-	-	-	
Standard deviation	0	-	-	-	0	0	-	-	-	-	0	-	-	-	-	-	-	
CV (%)	0	-	-	-	0	0	-	-	-	-	0	-	-	-	-	-	-	
Millet muslin	-	-	-	44.1	3.6	-	13.4	-	-	6.07	-	-	-	59.7	39.9	-	-	Amole et al., 2022
Average	-	-	-	44.1	3.6	-	13.4	-	-	6.07	-	-	-	59.7	39.9	-	-	Amole et al., 2022 (n=1)
Minimum	-	-	-	44.1	3.6	-	13.4	-	-	6.07	-	-	-	59.7	39.9	-	-	
Maximum	-	-	-	44.1	3.6	-	13.4	-	-	6.07	-	-	-	59.7	39.9	-	-	
Standard deviation	-	-	-	0	0	-	0	-	-	0	-	-	-	0	0	-	-	
CV (%)	-	-	-	0	0	-	0	-	-	0	-	-	-	0	0	-	-	

Table S5. Table of chemical composition of forages for ruminants in West-Africa

Feed	DM (%)	EE (% DM)	OM (% DM)	dOM (% DM)	CP (% DM)	CF (% DM)	Ash (% DM)	GE (MJ/kg DM)	DE (MJ/kg DM)	ME (MJ/kg DM)	DCP (g/kg DM)	UFL	UFV	NDF (% DM)	ADF (% DM)	Ca (% DM)	P (% DM)	References
Afzelia africana	40.69	-	89.34	-	13.67	-	10.66	-	-	-	-	0.65	-	-	-	-	-	Avornyo et al., 2020
Afzelia africana	-	6.38	83.57	-	24.69	-	16.43	-	-	-	-	-	-	37.05	24.45	0.77	0.67	Isah et al., 2015
Afzelia africana	38.7	-	95	43	16.8	38.6	-	-	-	-	121	0.58	0.47	72.3	69.7	-	-	Sidiimorou et al., 2016
Average	39.7	6.38	89.3	43	18.39	38.6	13.54	-	-	-	121	0.61	0.47	54.67	47.08	0.77	0.67	Avornyo et al., 2020; Isah et al., 2015; Sidiimorou et al., 2016 (n=3)
Minimum	38.7	6.38	83.57	43	13.67	38.6	10.66	-	-	-	121	0.58	0.47	37.05	24.45	0.77	0.67	
Maximum	40.69	6.38	95	43	24.69	38.6	16.43	-	-	-	121	0.65	0.47	72.3	69.7	0.77	0.67	
Standard deviation	1.41	0	5.72	0	5.68	0	4.08	-	-	-	0	0.05	0	24.93	32	0	0	
CV (%)	3.54	0	6.4	0	30.89	0	30.12	-	-	-	0	8.05	0	45.59	67.97	0	0	
Albizia ferruginea	-	-	-	-	16.5	-	-	-	-	-	-	-	-	61	45.3	-	-	Anyanwu and Etela, 2013
Average	-	-	-	-	16.5	-	-	-	-	-	-	-	-	61	45.3	-	-	Anyanwu and Etela, 2013 (n=1)
Minimum	-	-	-	-	16.5	-	-	-	-	-	-	-	-	61	45.3	-	-	
Maximum	-	-	-	-	16.5	-	-	-	-	-	-	-	-	61	45.3	-	-	
Standard deviation	-	-	-	-	0	-	-	-	-	-	-	-	-	0	0	-	-	
CV (%)	-	-	-	-	0	-	-	-	-	-	-	-	-	0	0	-	-	
Albizia glaberrima	-	1.31	96.16	14.65	25.23	-	3.84	-	-	-	-	-	-	67.26	51.85	-	-	Koura et al., 2021
Average	-	1.31	96.16	14.65	25.23	-	3.84	-	-	-	-	-	-	67.26	51.85	-	-	Koura et al., 2021 (n=1)
Minimum	-	1.31	96.16	14.65	25.23	-	3.84	-	-	-	-	-	-	67.26	51.85	-	-	
Maximum	-	1.31	96.16	14.65	25.23	-	3.84	-	-	-	-	-	-	67.26	51.85	-	-	
Standard deviation	-	0	0	0	0	-	0	-	-	-	-	-	-	0	0	-	-	
CV (%)	-	0	0	0	0	-	0	-	-	-	-	-	-	0	0	-	-	
Albizia gummifera	-	-	-	-	20.5	-	-	-	-	-	-	-	-	70.6	54.9	-	-	Anyanwu and Etela, 2013
Average	-	-	-	-	20.5	-	-	-	-	-	-	-	-	70.6	54.9	-	-	Anyanwu and Etela, 2013 (n=1)
Minimum	-	-	-	-	20.5	-	-	-	-	-	-	-	-	70.6	54.9	-	-	
Maximum	-	-	-	-	20.5	-	-	-	-	-	-	-	-	70.6	54.9	-	-	
Standard deviation	-	-	-	-	0	-	-	-	-	-	-	-	-	0	0	-	-	
CV (%)	-	-	-	-	0	-	-	-	-	-	-	-	-	0	0	-	-	
Albizia niopoides	-	-	-	-	19.9	-	-	-	-	-	-	-	-	58.8	46.7	-	-	Anyanwu and Etela, 2013
Average	-	-	-	-	19.9	-	-	-	-	-	-	-	-	58.8	46.7	-	-	Anyanwu and Etela, 2013 (n=1)
Minimum	-	-	-	-	19.9	-	-	-	-	-	-	-	-	58.8	46.7	-	-	
Maximum	-	-	-	-	19.9	-	-	-	-	-	-	-	-	58.8	46.7	-	-	
Standard deviation	-	-	-	-	0	-	-	-	-	-	-	-	-	0	0	-	-	
CV (%)	-	-	-	-	0	-	-	-	-	-	-	-	-	0	0	-	-	
Andropogon gayanus	27.79	8.7	90.27	-	5.9	27.2	9.73	-	-	-	-	-	-	66.13	34.97	-	-	Ogunbosoye and Odedire, 2022
Andropogon gayanus	-	-	-	-	4.38	7.1	-	-	-	-	-	-	-	-	-	0.027	0.05	Amegnaglo et al., 2018
Average	27.79	8.7	90.27	-	5.14	17.15	9.73	-	-	-	-	-	-	66.13	34.97	0.03	0.05	Ogunbosoye and Odedire, 2022; Amegnaglo et al., 2018 (n=2)
Minimum	27.79	8.7	90.27	-	4.38	7.1	9.73	-	-	-	-	-	-	66.13	34.97	0.03	0.05	
Maximum	27.79	8.7	90.27	-	5.9	27.2	9.73	-	-	-	-	-	-	66.13	34.97	0.03	0.05	
Standard deviation	0	0	0	-	1.07	14.21	0	-	-	-	-	-	-	0	0	0	0	
CV (%)	0	0	0	-	20.91	82.87	0	-	-	-	-	-	-	0	0	0	0	

Andropogon virginicus	90.82	1.1	88.43	51.03	7.84	-	11.57	-	-	5.84	-	-	-	73.88	47.52	-	-	Koura et al., 2022
Average	90.82	1.1	88.43	51.03	7.84	-	11.57	-	-	5.84	-	-	-	73.88	47.52	-	-	Koura et al., 2022 (n=1)
Minimum	90.82	1.1	88.43	51.03	7.84	-	11.57	-	-	5.84	-	-	-	73.88	47.52	-	-	
Maximum	90.82	1.1	88.43	51.03	7.84	-	11.57	-	-	5.84	-	-	-	73.88	47.52	-	-	
Standard deviation	0	0	0	0	0	-	0	-	-	0	-	-	-	0	0	-	-	
CV (%)	0	0	0	0	0	-	0	-	-	0	-	-	-	0	0	-	-	
Annona senegalensis	-	3.51	83.29	-	11.46	-	16.71	-	-	-	-	-	-	37.23	21.84	0.57	0.6	Isah et al., 2015
Annona senegalensis	34.9	-	93.1	43.2	10.5	21.2	-	-	-	-	62	0.6	0.5	51.5	43.2	-	-	Sidiimorou et al., 2016
Average	34.9	3.51	88.19	43.2	10.98	21.2	16.71	-	-	-	62	0.6	0.5	44.36	32.52	0.57	0.6	Isah et al., 2015; Sidiimorou et al., 2016 (n=2)
Minimum	34.9	3.51	83.29	43.2	10.5	21.2	16.71	-	-	-	62	0.6	0.5	37.23	21.84	0.57	0.6	
Maximum	34.9	3.51	93.1	43.2	11.46	21.2	16.71	-	-	-	62	0.6	0.5	51.5	43.2	0.57	0.6	
Standard deviation	0	0	6.94	0	0.68	0	0	-	-	-	0	0	0	10.09	15.1	0	0	
CV (%)	0	0	7.87	0	6.18	0	0	-	-	-	0	0	0	22.74	46.44	0	0	
Antiaris Africana	-	-	88.37	-	20.79	-	11.63	-	-	-	-	-	-	47.55	21.88	-	-	Koura et al., 2021
Average	-	-	88.37	-	20.79	-	11.63	-	-	-	-	-	-	47.55	21.88	-	-	Koura et al., 2021 (n=1)
Minimum	-	-	88.37	-	20.79	-	11.63	-	-	-	-	-	-	47.55	21.88	-	-	
Maximum	-	-	88.37	-	20.79	-	11.63	-	-	-	-	-	-	47.55	21.88	-	-	
Standard deviation	-	-	0	-	0	-	0	-	-	-	-	-	-	0	0	-	-	
CV (%)	-	-	0	-	0	-	0	-	-	-	-	-	-	0	0	-	-	
Antiaris africana	-	2.77	88.37	54.95	20.79	-	11.63	-	-	-	-	-	-	47.55	21.88	-	-	Koura et al., 2021
Antiaris africana	92.8007	3.5323	-	-	-	15.5142	16.7247	-	-	-	-	-	-	-	-	-	-	Mawussi et al., 2022
Average	92.8	3.15	88.37	54.95	20.79	15.51	14.18	-	-	-	-	-	-	47.55	21.88	-	-	Koura et al., 2021; Mawussi et al., 2022 (n=2)
Minimum	92.8	2.77	88.37	54.95	20.79	15.51	11.63	-	-	-	-	-	-	47.55	21.88	-	-	
Maximum	92.8	3.53	88.37	54.95	20.79	15.51	16.72	-	-	-	-	-	-	47.55	21.88	-	-	
Standard deviation	0	0.54	0	0	0	0	3.6	-	-	-	-	-	-	0	0	-	-	
CV (%)	0	17.11	0	0	0	0	25.41	-	-	-	-	-	-	0	0	-	-	
Arachis hypogea	28	-	81.48	-	9.04	-	18.52	-	-	-	-	0.58	0.51	-	-	-	-	Avornyo et al., 2020
Arachis hypogea	93.27	-	85.37	-	12.46	-	14.63	-	-	-	-	-	-	-	-	-	-	Sanou et al., 2016
Average	60.63	-	83.43	-	10.75	-	16.57	-	-	-	-	0.58	0.51	-	-	-	-	Avornyo et al., 2020; Sanou et al., 2016 (n=2)
Minimum	28	-	81.48	-	9.04	-	14.63	-	-	-	-	0.58	0.51	-	-	-	-	
Maximum	93.27	-	85.37	-	12.46	-	18.52	-	-	-	-	0.58	0.51	-	-	-	-	
Standard deviation	46.15	-	2.75	-	2.42	-	2.75	-	-	-	-	0	0	-	-	-	-	
CV (%)	76.12	-	3.3	-	22.5	-	16.6	-	-	-	-	0	0	-	-	-	-	
Bambusa balcooa	92.47	4.43	82.1	39.03	10.48	27.32	17.9	-	-	4	-	-	-	52.2	35.5	-	-	Sasu et al., 2023
Average	92.47	4.43	82.1	39.03	10.48	27.32	17.9	-	-	4	-	-	-	52.2	35.5	-	-	Sasu et al., 2023 (n=1)
Minimum	92.47	4.43	82.1	39.03	10.48	27.32	17.9	-	-	4	-	-	-	52.2	35.5	-	-	
Maximum	92.47	4.43	82.1	39.03	10.48	27.32	17.9	-	-	4	-	-	-	52.2	35.5	-	-	
Standard deviation	0	0	0	0	0	0	0	-	-	0	-	-	-	0	0	-	-	
CV (%)	0	0	0	0	0	0	0	-	-	0	-	-	-	0	0	-	-	
Bambusa vulgaris	-	3.36	83.35	38.23	19.02	-	16.65	-	-	-	-	-	-	68.69	38.37	-	-	Koura et al., 2021

Bambusa vulgaris	91.77	-	86.97	-	15.29	31.94	13.03	-	-	-	-	-	-	-	-	-	-	Sasu et al., 2023
Average	91.77	3.36	85.16	38.23	17.16	31.94	14.84	-	-	-	-	-	-	68.69	38.37	-	-	Koura et al., 2021; Sasu et al., 2023 (n=2)
Minimum	91.77	3.36	83.35	38.23	15.29	31.94	13.03	-	-	-	-	-	-	68.69	38.37	-	-	
Maximum	91.77	3.36	86.97	38.23	19.02	31.94	16.65	-	-	-	-	-	-	68.69	38.37	-	-	
Standard deviation	0	0	2.56	0	2.64	0	2.56	-	-	-	-	-	-	0	0	-	-	
CV (%)	0	0	3.01	0	15.37	0	17.25	-	-	-	-	-	-	0	0	-	-	
Batryospermum paradoxum	95.8	5	87.8	-	14.63	-	8	-	-	-	-	-	-	47.6	32.1	1.2	1.125	Njidda, 2011
Average	95.8	5	87.8	-	14.63	-	8	-	-	-	-	-	-	47.6	32.1	1.2	1.12	Njidda, 2011 (n=1)
Minimum	95.8	5	87.8	-	14.63	-	8	-	-	-	-	-	-	47.6	32.1	1.2	1.12	
Maximum	95.8	5	87.8	-	14.63	-	8	-	-	-	-	-	-	47.6	32.1	1.2	1.12	
Standard deviation	0	0	0	-	0	-	0	-	-	-	-	-	-	0	0	0	0	
CV (%)	0	0	0	-	0	-	0	-	-	-	-	-	-	0	0	0	0	
Bauhimia monandra	-	-	-	-	16.2	-	-	-	-	-	-	-	-	59.9	42.6	-	-	Anyanwu and Etela, 2013
Average	-	-	-	-	16.2	-	-	-	-	-	-	-	-	59.9	42.6	-	-	Anyanwu and Etela, 2013 (n=1)
Minimum	-	-	-	-	16.2	-	-	-	-	-	-	-	-	59.9	42.6	-	-	
Maximum	-	-	-	-	16.2	-	-	-	-	-	-	-	-	59.9	42.6	-	-	
Standard deviation	-	-	-	-	0	-	-	-	-	-	-	-	-	0	0	-	-	
CV (%)	-	-	-	-	0	-	-	-	-	-	-	-	-	0	0	-	-	
Berlinia grandiflora	-	-	-	-	14.1	-	-	-	-	-	-	-	-	68.2	54.3	-	-	Anyanwu and Etela, 2013
Average	-	-	-	-	14.1	-	-	-	-	-	-	-	-	68.2	54.3	-	-	Anyanwu and Etela, 2013 (n=1)
Minimum	-	-	-	-	14.1	-	-	-	-	-	-	-	-	68.2	54.3	-	-	
Maximum	-	-	-	-	14.1	-	-	-	-	-	-	-	-	68.2	54.3	-	-	
Standard deviation	-	-	-	-	0	-	-	-	-	-	-	-	-	0	0	-	-	
CV (%)	-	-	-	-	0	-	-	-	-	-	-	-	-	0	0	-	-	
Bicolor sorghum	94.93	-	94.02	-	1.14	-	5.98	-	-	-	-	0.71	-	-	-	-	-	Sanou et al., 2016
Average	94.93	-	94.02	-	1.14	-	5.98	-	-	-	-	0.71	-	-	-	-	-	Sanou et al., 2016 (n=1)
Minimum	94.93	-	94.02	-	1.14	-	5.98	-	-	-	-	0.71	-	-	-	-	-	
Maximum	94.93	-	94.02	-	1.14	-	5.98	-	-	-	-	0.71	-	-	-	-	-	
Standard deviation	0	-	0	-	0	-	0	-	-	-	-	0	-	-	-	-	-	
CV (%)	0	-	0	-	0	-	0	-	-	-	-	0	-	-	-	-	-	
Blighia sapida	-	3.54	91.25	37.1	18	-	8.75	-	-	-	-	-	-	64.18	15.16	-	-	Koura et al., 2021
Blighia sapida	90.5	3.32	89.9	-	17.2	13.2	10.2	-	-	-	-	-	-	48	34.8	-	-	Sasu et al., 2023
Average	90.5	3.43	90.58	37.1	17.6	13.2	9.47	-	-	-	-	-	-	56.09	24.98	-	-	Koura et al., 2021; Sasu et al., 2023 (n=2)
Minimum	90.5	3.32	89.9	37.1	17.2	13.2	8.75	-	-	-	-	-	-	48	15.16	-	-	
Maximum	90.5	3.54	91.25	37.1	18	13.2	10.2	-	-	-	-	-	-	64.18	34.8	-	-	
Standard deviation	0	0.16	0.95	0	0.57	0	1.03	-	-	-	-	-	-	11.44	13.89	-	-	
CV (%)	0	4.54	1.05	0	3.21	0	10.82	-	-	-	-	-	-	20.4	55.59	-	-	
Blighia sapida (Leaves)	-	-	91.25	-	18	-	8.75	-	-	-	-	-	-	64.18	15.16	-	-	Koura et al., 2021
Average	-	-	91.25	-	18	-	8.75	-	-	-	-	-	-	64.18	15.16	-	-	Koura et al., 2021 (n=1)
Minimum	-	-	91.25	-	18	-	8.75	-	-	-	-	-	-	64.18	15.16	-	-	
Maximum	-	-	91.25	-	18	-	8.75	-	-	-	-	-	-	64.18	15.16	-	-	

Standard deviation	-	-	0	-	0	-	0	-	-	-	-	-	-	0	0	-	-	
CV (%)	-	-	0	-	0	-	0	-	-	-	-	-	-	0	0	-	-	
Brachiaria decumbens	82.1	-	88.69	-	9.15	28.58	11.31	-	-	-	-	-	-	-	-	-	-	Sasu et al., 2023
Average	82.1	-	88.69	-	9.15	28.58	11.31	-	-	-	-	-	-	-	-	-	-	Sasu et al., 2023 (n=1)
Minimum	82.1	-	88.69	-	9.15	28.58	11.31	-	-	-	-	-	-	-	-	-	-	
Maximum	82.1	-	88.69	-	9.15	28.58	11.31	-	-	-	-	-	-	-	-	-	-	
Standard deviation	0	-	0	-	0	0	0	-	-	-	-	-	-	-	-	-	-	
CV (%)	0	-	0	-	0	0	0	-	-	-	-	-	-	-	-	-	-	
Brachiaria deflexa	91.03	1.31	91.9	55.27	7.07	-	8.1	-	-	5.56	-	-	-	75.92	41.87	-	-	Koura et al., 2022
Average	91.03	1.31	91.9	55.27	7.07	-	8.1	-	-	5.56	-	-	-	75.92	41.87	-	-	Koura et al., 2022 (n=1)
Minimum	91.03	1.31	91.9	55.27	7.07	-	8.1	-	-	5.56	-	-	-	75.92	41.87	-	-	
Maximum	91.03	1.31	91.9	55.27	7.07	-	8.1	-	-	5.56	-	-	-	75.92	41.87	-	-	
Standard deviation	0	0	0	0	0	-	0	-	-	0	-	-	-	0	0	-	-	
CV (%)	0	0	0	0	0	-	0	-	-	0	-	-	-	0	0	-	-	
Bush grass hay	85	-	-	-	9.2	30	-	-	-	-	6.5	0.55	-	-	-	-	-	Sanogo et al., 2019
Average	85	-	-	-	9.2	30	-	-	-	-	6.5	0.55	-	-	-	-	-	Sanogo et al., 2019 (n=1)
Minimum	85	-	-	-	9.2	30	-	-	-	-	6.5	0.55	-	-	-	-	-	
Maximum	85	-	-	-	9.2	30	-	-	-	-	6.5	0.55	-	-	-	-	-	
Standard deviation	0	-	-	-	0	0	-	-	-	-	0	0	-	-	-	-	-	
CV (%)	0	-	-	-	0	0	-	-	-	-	0	0	-	-	-	-	-	
Bush hay	93.6	-	91	-	3.1	39.4	9	-	-	-	0.3	0.71	-	81.2	46.4	-	-	Nantoumé et al., 2000
Average	93.6	-	91	-	3.1	39.4	9	-	-	-	0.3	0.71	-	81.2	46.4	-	-	Nantoumé et al., 2000 (n=1)
Minimum	93.6	-	91	-	3.1	39.4	9	-	-	-	0.3	0.71	-	81.2	46.4	-	-	
Maximum	93.6	-	91	-	3.1	39.4	9	-	-	-	0.3	0.71	-	81.2	46.4	-	-	
Standard deviation	0	-	0	-	0	0	0	-	-	-	0	0	-	0	0	-	-	
CV (%)	0	-	0	-	0	0	0	-	-	-	0	0	-	0	0	-	-	
Celtis integuifolis	96.2	3	80.2	-	15.89	-	16	-	-	-	-	-	-	42.3	31.2	1.95	1.125	Njidda, 2011
Average	96.2	3	80.2	-	15.89	-	16	-	-	-	-	-	-	42.3	31.2	1.95	1.12	Njidda, 2011 (n=1)
Minimum	96.2	3	80.2	-	15.89	-	16	-	-	-	-	-	-	42.3	31.2	1.95	1.12	
Maximum	96.2	3	80.2	-	15.89	-	16	-	-	-	-	-	-	42.3	31.2	1.95	1.12	
Standard deviation	0	0	0	-	0	-	0	-	-	-	-	-	-	0	0	0	0	
CV (%)	0	0	0	-	0	-	0	-	-	-	-	-	-	0	0	0	0	
Cenchorus biflorus	90.35	1.39	91.78	51.9	7.83	-	8.22	-	-	5.93	-	-	-	73.81	46.18	-	-	Koura et al., 2022
Average	90.35	1.39	91.78	51.9	7.83	-	8.22	-	-	5.93	-	-	-	73.81	46.18	-	-	Koura et al., 2022 (n=1)
Minimum	90.35	1.39	91.78	51.9	7.83	-	8.22	-	-	5.93	-	-	-	73.81	46.18	-	-	
Maximum	90.35	1.39	91.78	51.9	7.83	-	8.22	-	-	5.93	-	-	-	73.81	46.18	-	-	
Standard deviation	0	0	0	0	0	-	0	-	-	0	-	-	-	0	0	-	-	
CV (%)	0	0	0	0	0	-	0	-	-	0	-	-	-	0	0	-	-	
Cenchrus purpureus	79.07	-	93.57	-	8	44.4	6.43	-	-	-	-	0.51	-	-	-	-	-	Sasu et al., 2023
Average	79.07	-	93.57	-	8	44.4	6.43	-	-	-	-	0.51	-	-	-	-	-	Sasu et al., 2023 (n=1)
Minimum	79.07	-	93.57	-	8	44.4	6.43	-	-	-	-	0.51	-	-	-	-	-	
Maximum	79.07	-	93.57	-	8	44.4	6.43	-	-	-	-	0.51	-	-	-	-	-	
Standard deviation	0	-	0	-	0	0	0	-	-	-	-	0	-	-	-	-	-	

CV (%)	0	-	0	-	0	0	0	-	-	-	-	0	-	-	-	-	-	
Chamaecrista rotundifolia	90.99	3.34	94.01	52.94	13.19	-	5.99	-	-	9.84	-	-	-	54.79	-	-	-	Koura et al., 2022
Average	90.99	3.34	94.01	52.94	13.19	-	5.99	-	-	9.84	-	-	-	54.79	-	-	-	Koura et al., 2022 (n=1)
Minimum	90.99	3.34	94.01	52.94	13.19	-	5.99	-	-	9.84	-	-	-	54.79	-	-	-	
Maximum	90.99	3.34	94.01	52.94	13.19	-	5.99	-	-	9.84	-	-	-	54.79	-	-	-	
Standard deviation	0	0	0	0	0	-	0	-	-	0	-	-	-	0	-	-	-	
CV (%)	0	0	0	0	0	-	0	-	-	0	-	-	-	0	-	-	-	
Commelina benghalensis	89.51	-	80.82	-	10.51	-	19.18	-	-	-	-	-	-	52.24	31.54	-	-	Sanou et al., 2016
Commelina benghalensis	59.69	2.4	91.2	-	18.75	-	8.8	-	-	9.003131<br>2	-	-	-	-	-	-	-	Kouadio et al., 2024
Commelina benghalensis	94	1	-	-	4.29	9	4.5	-	-	-	-	-	-	-	-	0.5	3.83	Muftau and Musa, 2020
Average	81.07	1.7	86.01	-	11.18	9	10.83	-	-	9	-	-	-	52.24	31.54	0.5	3.83	Sanou et al., 2016; Kouadio et al., 2024; Muftau and Musa, 2020 (n=3)
Minimum	59.69	1	80.82	-	4.29	9	4.5	-	-	9	-	-	-	52.24	31.54	0.5	3.83	
Maximum	94	2.4	91.2	-	18.75	9	19.18	-	-	9	-	-	-	52.24	31.54	0.5	3.83	
Standard deviation	18.65	0.99	7.34	-	7.25	0	7.55	-	-	0	-	-	-	0	0	0	0	
CV (%)	23	58.23	8.53	-	64.86	0	69.71	-	-	0	-	-	-	0	0	0	0	
Crotalaria retusa (dried leaves in the shade)	96.67	-	-	-	14	29.91	-	7.72	-	-	-	-	-	-	-	-	-	Yashim et al., 2012
Average	96.67	-	-	-	14	29.91	-	7.72	-	-	-	-	-	-	-	-	-	Yashim et al., 2012 (n=1)
Minimum	96.67	-	-	-	14	29.91	-	7.72	-	-	-	-	-	-	-	-	-	
Maximum	96.67	-	-	-	14	29.91	-	7.72	-	-	-	-	-	-	-	-	-	
Standard deviation	0	-	-	-	0	0	-	0	-	-	-	-	-	-	-	-	-	
CV (%)	0	-	-	-	0	0	-	0	-	-	-	-	-	-	-	-	-	
Crotalaria retusa (fresh leaves)	58.06	-	93.13	-	15.69	24.55	6.87	-	-	-	-	-	-	-	-	-	-	Yashim et al., 2012
Average	58.06	-	93.13	-	15.69	24.55	6.87	-	-	-	-	-	-	-	-	-	-	Yashim et al., 2012 (n=1)
Minimum	58.06	-	93.13	-	15.69	24.55	6.87	-	-	-	-	-	-	-	-	-	-	
Maximum	58.06	-	93.13	-	15.69	24.55	6.87	-	-	-	-	-	-	-	-	-	-	
Standard deviation	0	-	0	-	0	0	0	-	-	-	-	-	-	-	-	-	-	
CV (%)	0	-	0	-	0	0	0	-	-	-	-	-	-	-	-	-	-	
Crotalaria retusa (oven-dried leaves)	99.42	-	92.91	-	15.44	24.42	7.09	-	-	-	-	-	-	-	-	-	-	Yashim et al., 2012
Average	99.42	-	92.91	-	15.44	24.42	7.09	-	-	-	-	-	-	-	-	-	-	Yashim et al., 2012 (n=1)
Minimum	99.42	-	92.91	-	15.44	24.42	7.09	-	-	-	-	-	-	-	-	-	-	
Maximum	99.42	-	92.91	-	15.44	24.42	7.09	-	-	-	-	-	-	-	-	-	-	
Standard deviation	0	-	0	-	0	0	0	-	-	-	-	-	-	-	-	-	-	
CV (%)	0	-	0	-	0	0	0	-	-	-	-	-	-	-	-	-	-	
Crotalaria retusa (sun-dried leaves)	98.3	-	93.98	-	12.31	22.86	6.02	-	-	-	-	-	-	-	-	-	-	Yashim et al., 2012
Average	98.3	-	93.98	-	12.31	22.86	6.02	-	-	-	-	-	-	-	-	-	-	Yashim et al., 2012 (n=1)

Minimum	98.3	-	93.98	-	12.31	22.86	6.02	-	-	-	-	-	-	-	-	-	-	
Maximum	98.3	-	93.98	-	12.31	22.86	6.02	-	-	-	-	-	-	-	-	-	-	
Standard deviation	0	-	0	-	0	0	0	-	-	-	-	-	-	-	-	-	-	
CV (%)	0	-	0	-	0	0	0	-	-	-	-	-	-	-	-	-	-	
Dactyloctenium aegyptium	88.45	1.79	89.97	55.09	8.23	-	10.03	-	-	5.98	-	-	-	70.61	46.33	-	-	Koura et al., 2022
Average	88.45	1.79	89.97	55.09	8.23	-	10.03	-	-	5.98	-	-	-	70.61	46.33	-	-	Koura et al., 2022 (n=1)
Minimum	88.45	1.79	89.97	55.09	8.23	-	10.03	-	-	5.98	-	-	-	70.61	46.33	-	-	
Maximum	88.45	1.79	89.97	55.09	8.23	-	10.03	-	-	5.98	-	-	-	70.61	46.33	-	-	
Standard deviation	0	0	0	0	0	-	0	-	-	0	-	-	-	0	0	-	-	
CV (%)	0	0	0	0	0	-	0	-	-	0	-	-	-	0	0	-	-	
Dalbergia sissoo	-	-	-	-	16.1	-	-	-	-	-	-	-	-	55.9	35.4	-	-	Anyanwu and Etela, 2013
Average	-	-	-	-	16.1	-	-	-	-	-	-	-	-	55.9	35.4	-	-	Anyanwu and Etela, 2013 (n=1)
Minimum	-	-	-	-	16.1	-	-	-	-	-	-	-	-	55.9	35.4	-	-	
Maximum	-	-	-	-	16.1	-	-	-	-	-	-	-	-	55.9	35.4	-	-	
Standard deviation	-	-	-	-	0	-	-	-	-	-	-	-	-	0	0	-	-	
CV (%)	-	-	-	-	0	-	-	-	-	-	-	-	-	0	0	-	-	
Daniellia oliveri (Leaves)	-	-	84.84	-	14.48	-	-	-	-	-	-	-	-	47.89	26.27	-	-	Isah et al., 2015
Daniellia oliveri (Leaves)	-	-	-	70.5	18.9	-	6.4	-	-	10.19	-	-	-	56.9	42.2	-	-	Amole et al., 2022
Average	-	-	84.84	70.5	16.69	-	6.4	-	-	10.19	-	-	-	52.39	34.23	-	-	Isah et al., 2015; Amole et al., 2022 (n=2)
Minimum	-	-	84.84	70.5	14.48	-	6.4	-	-	10.19	-	-	-	47.89	26.27	-	-	
Maximum	-	-	84.84	70.5	18.9	-	6.4	-	-	10.19	-	-	-	56.9	42.2	-	-	
Standard deviation	-	-	0	0	3.13	-	0	-	-	0	-	-	-	6.37	11.26	-	-	
CV (%)	-	-	0	0	18.73	-	0	-	-	0	-	-	-	12.16	32.9	-	-	
Daniellia oliveri	-	3.75	84.84	-	14.48	-	15.16	-	-	-	-	-	-	47.89	26.27	0.9	0.23	Isah et al., 2015
Daniellia oliveri	42.7	-	96.6	54.6	18.5	35.8	-	-	-	-	137	0.73	0.64	76.6	67.5	-	-	Sidiimorou et al., 2016
Average	42.7	3.75	90.72	54.6	16.49	35.8	15.16	-	-	-	137	0.73	0.64	62.24	46.88	0.9	0.23	Isah et al., 2015; Sidiimorou et al., 2016 (n=2)
Minimum	42.7	3.75	84.84	54.6	14.48	35.8	15.16	-	-	-	137	0.73	0.64	47.89	26.27	0.9	0.23	
Maximum	42.7	3.75	96.6	54.6	18.5	35.8	15.16	-	-	-	137	0.73	0.64	76.6	67.5	0.9	0.23	
Standard deviation	0	0	8.32	0	2.84	0	0	-	-	-	0	0	0	20.3	29.15	0	0	
CV (%)	0	0	9.17	0	17.24	0	0	-	-	-	0	0	0	32.61	62.18	0	0	
Dialium guineense	-	-	-	-	12.1	-	-	-	-	-	-	-	-	61.9	48.5	-	-	Anyanwu and Etela, 2013
Average	-	-	-	-	12.1	-	-	-	-	-	-	-	-	61.9	48.5	-	-	Anyanwu and Etela, 2013 (n=1)
Minimum	-	-	-	-	12.1	-	-	-	-	-	-	-	-	61.9	48.5	-	-	
Maximum	-	-	-	-	12.1	-	-	-	-	-	-	-	-	61.9	48.5	-	-	
Standard deviation	-	-	-	-	0	-	-	-	-	-	-	-	-	0	0	-	-	
CV (%)	-	-	-	-	0	-	-	-	-	-	-	-	-	0	0	-	-	
Dichapetalum guineense	-	3.13	87.38	50.39	14.33	-	12.62	-	-	-	-	-	-	55.06	28.12	-	-	Koura et al., 2021
Average	-	3.13	87.38	50.39	14.33	-	12.62	-	-	-	-	-	-	55.06	28.12	-	-	Koura et al., 2021 (n=1)
Minimum	-	3.13	87.38	50.39	14.33	-	12.62	-	-	-	-	-	-	55.06	28.12	-	-	
Maximum	-	3.13	87.38	50.39	14.33	-	12.62	-	-	-	-	-	-	55.06	28.12	-	-	

Standard deviation	-	0	0	0	0	-	0	-	-	-	-	-	-	0	0	-	-	
CV (%)	-	0	0	0	0	-	0	-	-	-	-	-	-	0	0	-	-	
Digitaria horizontalis	93.78	-	87.5	-	6.89	-	12.5	-	-	-	-	0.6	-	-	-	-	-	Sanou et al., 2016
Digitaria horizontalis	-	-	-	-	3.63	10	-	-	-	-	-	-	-	-	-	0.035	0.036	Amegnaglo et al., 2018
Average	93.78	-	87.5	-	5.26	10	12.5	-	-	-	-	0.6	-	-	-	0.04	0.04	Sanou et al., 2016; Amegnaglo et al., 2018 (n=2)
Minimum	93.78	-	87.5	-	3.63	10	12.5	-	-	-	-	0.6	-	-	-	0.04	0.04	
Maximum	93.78	-	87.5	-	6.89	10	12.5	-	-	-	-	0.6	-	-	-	0.04	0.04	
Standard deviation	0	-	0	-	2.31	0	0	-	-	-	-	0	-	-	-	0	0	
CV (%)	0	-	0	-	43.82	0	0	-	-	-	-	0	-	-	-	0	0	
Echinochloa colona	92.78	-	84.84	-	1.14	-	15.16	-	-	-	-	0.58	-	-	-	-	-	Sanou et al., 2016
Average	92.78	-	84.84	-	1.14	-	15.16	-	-	-	-	0.58	-	-	-	-	-	Sanou et al., 2016 (n=1)
Minimum	92.78	-	84.84	-	1.14	-	15.16	-	-	-	-	0.58	-	-	-	-	-	
Maximum	92.78	-	84.84	-	1.14	-	15.16	-	-	-	-	0.58	-	-	-	-	-	
Standard deviation	0	-	0	-	0	-	0	-	-	-	-	0	-	-	-	-	-	
CV (%)	0	-	0	-	0	-	0	-	-	-	-	0	-	-	-	-	-	
Echinochloa stagnina	92.93	-	87.5	-	8.12	-	12.5	-	-	-	-	0.63	-	-	-	-	-	Sanou et al., 2016
Average	92.93	-	87.5	-	8.12	-	12.5	-	-	-	-	0.63	-	-	-	-	-	Sanou et al., 2016 (n=1)
Minimum	92.93	-	87.5	-	8.12	-	12.5	-	-	-	-	0.63	-	-	-	-	-	
Maximum	92.93	-	87.5	-	8.12	-	12.5	-	-	-	-	0.63	-	-	-	-	-	
Standard deviation	0	-	0	-	0	-	0	-	-	-	-	0	-	-	-	-	-	
CV (%)	0	-	0	-	0	-	0	-	-	-	-	0	-	-	-	-	-	
Elaeis guineensis	-	3.81	92	55.39	14.01	-	8	-	-	-	-	-	-	53.66	32.66	-	-	Koura et al., 2021 (GCZ)
Elaeis guineensis	-	2.58	91.51	54.47	16.55	-	8.49	-	-	-	-	-	-	50.44	32.64	-	-	Koura et al., 2021 (SGZ)
Elaeis guineensis	90.32	1.1	90.16	58.16	14.01	-	9.84	-	-	10.36	-	-	-	53.66	36.97	-	-	Koura et al., 2022
Average	90.32	2.5	91.22	56.01	14.86	-	8.78	-	-	10.36	-	-	-	52.59	34.09	-	-	Koura et al., 2021 (GCZ); Koura et al., 2021 (SGZ); Koura et al., 2022 (n=3)
Minimum	90.32	1.1	90.16	54.47	14.01	-	8	-	-	10.36	-	-	-	50.44	32.64	-	-	
Maximum	90.32	3.81	92	58.16	16.55	-	9.84	-	-	10.36	-	-	-	53.66	36.97	-	-	
Standard deviation	0	1.36	0.95	1.92	1.47	-	0.95	-	-	0	-	-	-	1.86	2.49	-	-	
CV (%)	0	54.35	1.04	3.43	9.87	-	10.86	-	-	0	-	-	-	3.54	7.32	-	-	
Elaeis guineensis (from the Guineo-Congolian zone of Benin)	-	-	92	-	14.04	-	8	-	-	-	-	-	-	53.66	32.66	-	-	Koura et al., 2021
Average	-	-	92	-	14.04	-	8	-	-	-	-	-	-	53.66	32.66	-	-	Koura et al., 2021 (n=1)
Minimum	-	-	92	-	14.04	-	8	-	-	-	-	-	-	53.66	32.66	-	-	
Maximum	-	-	92	-	14.04	-	8	-	-	-	-	-	-	53.66	32.66	-	-	
Standard deviation	-	-	0	-	0	-	0	-	-	-	-	-	-	0	0	-	-	
CV (%)	-	-	0	-	0	-	0	-	-	-	-	-	-	0	0	-	-	
Elaeis guineensis (from the Sudano-Guinean zone of Benin)	-	-	91.51	-	16.55	-	8.49	-	-	-	-	-	-	50.44	32.64	-	-	Koura et al., 2021
Average	-	-	91.51	-	16.55	-	8.49	-	-	-	-	-	-	50.44	32.64	-	-	Koura et al., 2021 (n=1)
Minimum	-	-	91.51	-	16.55	-	8.49	-	-	-	-	-	-	50.44	32.64	-	-	

Maximum	-	-	91.51	-	16.55	-	8.49	-	-	-	-	-	-	50.44	32.64	-	-	
Standard deviation	-	-	0	-	0	-	0	-	-	-	-	-	-	0	0	-	-	
CV (%)	-	-	0	-	0	-	0	-	-	-	-	-	-	0	0	-	-	
Elaeis guineensis leaves	-	2.1	90.7	-	9	-	9.3	19.8	-	-	-	-	-	70	54.5	-	-	Osakwe et al., 2004
Average	-	2.1	90.7	-	9	-	9.3	19.8	-	-	-	-	-	70	54.5	-	-	Osakwe et al., 2004 (n=1)
Minimum	-	2.1	90.7	-	9	-	9.3	19.8	-	-	-	-	-	70	54.5	-	-	
Maximum	-	2.1	90.7	-	9	-	9.3	19.8	-	-	-	-	-	70	54.5	-	-	
Standard deviation	-	0	0	-	0	-	0	0	-	-	-	-	-	0	0	-	-	
CV (%)	-	0	0	-	0	-	0	0	-	-	-	-	-	0	0	-	-	
Eleusine indica	94.86	-	78.36	-	1.15	-	21.64	-	-	-	-	-	-	56.4	33.2	-	-	Sanou et al., 2016
Eleusine indica	94.5668	2.87	-	-	-	20.7245	12.9514	-	-	-	-	-	-	-	-	-	-	Mawussi et al., 2022
Average	94.71	2.87	78.36	-	1.15	20.72	17.3	-	-	-	-	-	-	56.4	33.2	-	-	Sanou et al., 2016; Mawussi et al., 2022 (n=2)
Minimum	94.57	2.87	78.36	-	1.15	20.72	12.95	-	-	-	-	-	-	56.4	33.2	-	-	
Maximum	94.86	2.87	78.36	-	1.15	20.72	21.64	-	-	-	-	-	-	56.4	33.2	-	-	
Standard deviation	0.21	0	0	-	0	0	6.14	-	-	-	-	-	-	0	0	-	-	
CV (%)	0.22	0	0	-	0	0	35.52	-	-	-	-	-	-	0	0	-	-	
Enterolobium cyclocarpum	-	-	-	-	19.3	-	-	-	-	-	-	-	-	61.6	45.7	-	-	Anyanwu and Etela, 2013
Average	-	-	-	-	19.3	-	-	-	-	-	-	-	-	61.6	45.7	-	-	Anyanwu and Etela, 2013 (n=1)
Minimum	-	-	-	-	19.3	-	-	-	-	-	-	-	-	61.6	45.7	-	-	
Maximum	-	-	-	-	19.3	-	-	-	-	-	-	-	-	61.6	45.7	-	-	
Standard deviation	-	-	-	-	0	-	-	-	-	-	-	-	-	0	0	-	-	
CV (%)	-	-	-	-	0	-	-	-	-	-	-	-	-	0	0	-	-	
Eragrostis tremula	91.75	1.16	95.01	42.3	6.45	-	4.99	-	-	5.13	-	-	-	81.8	-	-	-	Koura et al., 2022
Eragrostis tremula	98.5	2	-	-	7.61	14	4.5	-	-	-	-	-	-	-	-	0.8	4.2	Muftau and Musa, 2020
Average	95.12	1.58	95.01	42.3	7.03	14	4.75	-	-	5.13	-	-	-	81.8	-	0.8	4.2	Koura et al., 2022; Muftau and Musa, 2020 (n=2)
Minimum	91.75	1.16	95.01	42.3	6.45	14	4.5	-	-	5.13	-	-	-	81.8	-	0.8	4.2	
Maximum	98.5	2	95.01	42.3	7.61	14	4.99	-	-	5.13	-	-	-	81.8	-	0.8	4.2	
Standard deviation	4.77	0.59	0	0	0.82	0	0.35	-	-	0	-	-	-	0	-	0	0	
CV (%)	5.02	37.59	0	0	11.67	0	7.3	-	-	0	-	-	-	0	-	0	0	
Faidherbia albida (Crushed pods)	90	-	96.2	-	11.4	-	3.8	-	-	-	-	-	-	41.8	30.7	-	-	Sana et al., 2021
Average	90	-	96.2	-	11.4	-	3.8	-	-	-	-	-	-	41.8	30.7	-	-	Sana et al., 2021 (n=1)
Minimum	90	-	96.2	-	11.4	-	3.8	-	-	-	-	-	-	41.8	30.7	-	-	
Maximum	90	-	96.2	-	11.4	-	3.8	-	-	-	-	-	-	41.8	30.7	-	-	
Standard deviation	0	-	0	-	0	-	0	-	-	-	-	-	-	0	0	-	-	
CV (%)	0	-	0	-	0	-	0	-	-	-	-	-	-	0	0	-	-	
Ficus gnaphalocarpa	36.35	-	75.86	-	11.59	-	24.14	-	-	-	-	-	-	66.93	50.6	-	-	Avornyo et al., 2020
Ficus gnaphalocarpa	60.3	-	96	35.9	9.6	18.7	-	-	-	-	54	0.47	0.35	45.4	44.1	-	-	Sidiimorou et al., 2016
Average	48.33	-	85.93	35.9	10.59	18.7	24.14	-	-	-	54	0.47	0.35	56.17	47.35	-	-	Avornyo et al., 2020; Sidiimorou et al., 2016 (n=2)
Minimum	36.35	-	75.86	35.9	9.6	18.7	24.14	-	-	-	54	0.47	0.35	45.4	44.1	-	-	

Maximum	60.3	-	96	35.9	11.59	18.7	24.14	-	-	-	54	0.47	0.35	66.93	50.6	-	-	
Standard deviation	16.94	-	14.24	0	1.41	0	0	-	-	-	0	0	0	15.22	4.6	-	-	
CV (%)	35.04	-	16.57	0	13.28	0	0	-	-	-	0	0	0	27.11	9.71	-	-	
Ficus polita	95.2	3	85.2	-	16.21	-	10	-	-	-	-	-	-	37.3	27.2	1.75	0.5	Njidda, 2011
Average	95.2	3	85.2	-	16.21	-	10	-	-	-	-	-	-	37.3	27.2	1.75	0.5	Njidda, 2011 (n=1)
Minimum	95.2	3	85.2	-	16.21	-	10	-	-	-	-	-	-	37.3	27.2	1.75	0.5	
Maximum	95.2	3	85.2	-	16.21	-	10	-	-	-	-	-	-	37.3	27.2	1.75	0.5	
Standard deviation	0	0	0	-	0	-	0	-	-	-	-	-	-	0	0	0	0	
CV (%)	0	0	0	-	0	-	0	-	-	-	-	-	-	0	0	0	0	
Ficus thonningii	-	3.36	84.33	60.7	13.48	-	15.67	-	-	-	-	-	-	46.47	24.01	-	-	Koura et al., 2021
Average	-	3.36	84.33	60.7	13.48	-	15.67	-	-	-	-	-	-	46.47	24.01	-	-	Koura et al., 2021 (n=1)
Minimum	-	3.36	84.33	60.7	13.48	-	15.67	-	-	-	-	-	-	46.47	24.01	-	-	
Maximum	-	3.36	84.33	60.7	13.48	-	15.67	-	-	-	-	-	-	46.47	24.01	-	-	
Standard deviation	-	0	0	0	0	-	0	-	-	-	-	-	-	0	0	-	-	
CV (%)	-	0	0	0	0	-	0	-	-	-	-	-	-	0	0	-	-	
Ficus thonningii (Leaves)	-	-	84.33	-	13.48	-	15.67	-	-	-	-	-	-	46.47	24.01	-	-	Koura et al., 2021
Ficus thonningii (Leaves)	95.2	2	77.2	-	16.47	-	18	-	-	-	-	-	-	51.2	41.2	0.75	0.4125	Njidda, 2011
Average	95.2	2	80.77	-	14.97	-	16.84	-	-	-	-	-	-	48.84	32.61	0.75	0.41	Koura et al., 2021; Njidda, 2011 (n=2)
Minimum	95.2	2	77.2	-	13.48	-	15.67	-	-	-	-	-	-	46.47	24.01	0.75	0.41	
Maximum	95.2	2	84.33	-	16.47	-	18	-	-	-	-	-	-	51.2	41.2	0.75	0.41	
Standard deviation	0	0	5.04	-	2.11	-	1.65	-	-	-	-	-	-	3.34	12.16	0	0	
CV (%)	0	0	6.24	-	14.12	-	9.79	-	-	-	-	-	-	6.85	37.28	0	0	
Ficus umbellata	-	3.56	89.48	41.45	15.42	-	10.52	-	-	-	-	-	-	45.66	24.8	-	-	Koura et al., 2021 (GCZ)
Ficus umbellata	-	4.72	88.09	49.57	15.92	-	11.91	-	-	-	-	-	-	46.06	33.45	-	-	Koura et al., 2021 (SGZ)
Average	-	4.14	88.78	45.51	15.67	-	11.21	-	-	-	-	-	-	45.86	29.12	-	-	Koura et al., 2021 (GCZ); Koura et al., 2021 (SGZ) (n=2)
Minimum	-	3.56	88.09	41.45	15.42	-	10.52	-	-	-	-	-	-	45.66	24.8	-	-	
Maximum	-	4.72	89.48	49.57	15.92	-	11.91	-	-	-	-	-	-	46.06	33.45	-	-	
Standard deviation	-	0.82	0.98	5.74	0.35	-	0.98	-	-	-	-	-	-	0.28	6.12	-	-	
CV (%)	-	19.81	1.11	12.62	2.26	-	8.76	-	-	-	-	-	-	0.62	21	-	-	
Ficus umbellata (from the Guineo-Congolian zone of Benin)	-	-	89.48	-	15.42	-	10.52	-	-	-	-	-	-	45.66	24.8	-	-	Koura et al., 2021
Average	-	-	89.48	-	15.42	-	10.52	-	-	-	-	-	-	45.66	24.8	-	-	Koura et al., 2021 (n=1)
Minimum	-	-	89.48	-	15.42	-	10.52	-	-	-	-	-	-	45.66	24.8	-	-	
Maximum	-	-	89.48	-	15.42	-	10.52	-	-	-	-	-	-	45.66	24.8	-	-	
Standard deviation	-	-	0	-	0	-	0	-	-	-	-	-	-	0	0	-	-	
CV (%)	-	-	0	-	0	-	0	-	-	-	-	-	-	0	0	-	-	
Ficus umbellata (from the Sudano-Guinean zone of Benin)	-	-	88.09	-	15.92	-	11.91	-	-	-	-	-	-	46.06	33.45	-	-	Koura et al., 2021
Average	-	-	88.09	-	15.92	-	11.91	-	-	-	-	-	-	46.06	33.45	-	-	Koura et al., 2021 (n=1)

Minimum	-	-	88.09	-	15.92	-	11.91	-	-	-	-	-	-	46.06	33.45	-	-	
Maximum	-	-	88.09	-	15.92	-	11.91	-	-	-	-	-	-	46.06	33.45	-	-	
Standard deviation	-	-	0	-	0	-	0	-	-	-	-	-	-	0	0	-	-	
CV (%)	-	-	0	-	0	-	0	-	-	-	-	-	-	0	0	-	-	
Gliricidia sepium	31.2	-	90.7	-	25.1	13.5	9.3	-	17.8	-	-	-	-	-	-	-	-	Idrissou et al., 2017
Gliricidia sepium	-	5.95	88.83	57.5	17.35	-	11.17	-	-	-	-	-	-	37.97	25.5	-	-	Koura et al., 2021
Gliricidia sepium	89.26	1.14	88.03	-	15.68	16.92	11.97	12.59384	-	-	-	-	-	64.89	41.37	-	-	Lamidi and Ogunkunle, 2015
Gliricidia sepium	-	-	92.7	-	24.7	-	7.3	17.19	-	-	-	-	-	39.2	24.8	1.38	0.31	Oduguwa et al., 2013
Gliricidia sepium	92.7189	3.3622	-	-	-	8.8875	10.6196	-	-	-	-	-	-	-	-	-	-	Mawussi et al., 2022
Average	71.06	3.48	90.06	57.5	20.71	13.1	10.07	14.89	17.8	-	-	-	-	47.35	30.56	1.38	0.31	Idrissou et al., 2017; Koura et al., 2021; Lamidi and Ogunkunle, 2015; Oduguwa et al., 2013; Mawussi et al., 2022 (n=5)
Minimum	31.2	1.14	88.03	57.5	15.68	8.89	7.3	12.59	17.8	-	-	-	-	37.97	24.8	1.38	0.31	
Maximum	92.72	5.95	92.7	57.5	25.1	16.92	11.97	17.19	17.8	-	-	-	-	64.89	41.37	1.38	0.31	
Standard deviation	34.56	2.41	2.08	0	4.89	4.03	1.83	3.25	0	-	-	-	-	15.2	9.37	0	0	
CV (%)	48.64	69.09	2.31	0	23.62	30.76	18.16	21.82	0	-	-	-	-	32.1	30.67	0	0	
Gliricidia sepium leaves	-	-	92.7	-	24.7	-	7.3	17.2	-	-	-	-	-	32.9	21.6	0.36	0.45	Oduguwa et al., 2013
Average	-	-	92.7	-	24.7	-	7.3	17.2	-	-	-	-	-	32.9	21.6	0.36	0.45	Oduguwa et al., 2013 (n=1)
Minimum	-	-	92.7	-	24.7	-	7.3	17.2	-	-	-	-	-	32.9	21.6	0.36	0.45	
Maximum	-	-	92.7	-	24.7	-	7.3	17.2	-	-	-	-	-	32.9	21.6	0.36	0.45	
Standard deviation	-	-	0	-	0	-	0	0	-	-	-	-	-	0	0	0	0	
CV (%)	-	-	0	-	0	-	0	0	-	-	-	-	-	0	0	0	0	
Gmelina arborea	89.82	1.62	87.11	-	14.51	13.49	12.89	12.46832	-	-	-	-	-	65.86	42.38	-	-	Lamidi and Ogunkunle, 2015
Gmelina arborea	76.65	3.3	91.11	-	22.29	10.09	8.89	17.61	-	-	-	-	-	-	-	-	-	Okagbare et al., 2004
Average	83.23	2.46	89.11	-	18.4	11.79	10.89	15.04	-	-	-	-	-	65.86	42.38	-	-	Lamidi and Ogunkunle, 2015; Okagbare et al., 2004 (n=2)
Minimum	76.65	1.62	87.11	-	14.51	10.09	8.89	12.47	-	-	-	-	-	65.86	42.38	-	-	
Maximum	89.82	3.3	91.11	-	22.29	13.49	12.89	17.61	-	-	-	-	-	65.86	42.38	-	-	
Standard deviation	9.31	1.19	2.83	-	5.5	2.4	2.83	3.64	-	-	-	-	-	0	0	-	-	
CV (%)	11.19	48.29	3.17	-	29.9	20.39	25.97	24.17	-	-	-	-	-	0	0	-	-	
Griffonia simplicifolia	80.42	10.4	91.7	-	25.9	-	8.3	-	-	14.00937088	-	-	-	-	-	-	-	Kouadio et al., 2024
Griffonia simplicifolia	93.1523	1.0969	-	-	-	18.2237	12.4505	-	-	-	-	-	-	-	-	-	-	Mawussi et al., 2022
Average	86.79	5.75	91.7	-	25.9	18.22	10.38	-	-	14.01	-	-	-	-	-	-	-	Kouadio et al., 2024; Mawussi et al., 2022 (n=2)
Minimum	80.42	1.1	91.7	-	25.9	18.22	8.3	-	-	14.01	-	-	-	-	-	-	-	
Maximum	93.15	10.4	91.7	-	25.9	18.22	12.45	-	-	14.01	-	-	-	-	-	-	-	
Standard deviation	9	6.58	0	-	0	0	2.93	-	-	0	-	-	-	-	-	-	-	
CV (%)	10.37	114.44	0	-	0	0	28.29	-	-	0	-	-	-	-	-	-	-	
Imperial Naples	-	-	-	-	10.7	-	-	-	-	-	-	-	-	58.4	52.9	-	-	Anyanwu and Etela, 2013
Average	-	-	-	-	10.7	-	-	-	-	-	-	-	-	58.4	52.9	-	-	Anyanwu and Etela, 2013 (n=1)
Minimum	-	-	-	-	10.7	-	-	-	-	-	-	-	-	58.4	52.9	-	-	

Maximum	-	-	-	-	10.7	-	-	-	-	-	-	-	-	58.4	52.9	-	-	
Standard deviation	-	-	-	-	0	-	-	-	-	-	-	-	-	0	0	-	-	
CV (%)	-	-	-	-	0	-	-	-	-	-	-	-	-	0	0	-	-	
Ipomoea batatas	92.87	-	88.63	-	10.45	-	11.37	-	-	-	-	-	-	-	-	-	-	Sanou et al., 2016
Average	92.87	-	88.63	-	10.45	-	11.37	-	-	-	-	-	-	-	-	-	-	Sanou et al., 2016 (n=1)
Minimum	92.87	-	88.63	-	10.45	-	11.37	-	-	-	-	-	-	-	-	-	-	
Maximum	92.87	-	88.63	-	10.45	-	11.37	-	-	-	-	-	-	-	-	-	-	
Standard deviation	0	-	0	-	0	-	0	-	-	-	-	-	-	-	-	-	-	
CV (%)	0	-	0	-	0	-	0	-	-	-	-	-	-	-	-	-	-	
Ipomoea eriocarpa	94.35	-	88.21	-	7	-	11.79	-	-	-	-	-	-	-	-	-	-	Sanou et al., 2016
Average	94.35	-	88.21	-	7	-	11.79	-	-	-	-	-	-	-	-	-	-	Sanou et al., 2016 (n=1)
Minimum	94.35	-	88.21	-	7	-	11.79	-	-	-	-	-	-	-	-	-	-	
Maximum	94.35	-	88.21	-	7	-	11.79	-	-	-	-	-	-	-	-	-	-	
Standard deviation	0	-	0	-	0	-	0	-	-	-	-	-	-	-	-	-	-	
CV (%)	0	-	0	-	0	-	0	-	-	-	-	-	-	-	-	-	-	
Jussiaea perennis	-	4.66	93.9	24.37	11.15	-	6.1	-	-	-	-	-	-	61.18	37.92	-	-	Koura et al., 2021
Average	-	4.66	93.9	24.37	11.15	-	6.1	-	-	-	-	-	-	61.18	37.92	-	-	Koura et al., 2021 (n=1)
Minimum	-	4.66	93.9	24.37	11.15	-	6.1	-	-	-	-	-	-	61.18	37.92	-	-	
Maximum	-	4.66	93.9	24.37	11.15	-	6.1	-	-	-	-	-	-	61.18	37.92	-	-	
Standard deviation	-	0	0	0	0	-	0	-	-	-	-	-	-	0	0	-	-	
CV (%)	-	0	0	0	0	-	0	-	-	-	-	-	-	0	0	-	-	
Khaya senegalensis (Leaves)	97	3	87	-	14.11	-	10	-	-	-	-	-	-	44.6	32.1	0.95	0.2625	Njidda, 2011
Khaya senegalensis (Leaves)	-	-	-	52.2	14.9	-	12.7	-	-	7.17	-	-	-	42.5	34.7	-	-	Amole et al., 2022
Average	97	3	87	52.2	14.5	-	11.35	-	-	7.17	-	-	-	43.55	33.4	0.95	0.26	Njidda, 2011; Amole et al., 2022 (n=2)
Minimum	97	3	87	52.2	14.11	-	10	-	-	7.17	-	-	-	42.5	32.1	0.95	0.26	
Maximum	97	3	87	52.2	14.9	-	12.7	-	-	7.17	-	-	-	44.6	34.7	0.95	0.26	
Standard deviation	0	0	0	0	0.56	-	1.91	-	-	0	-	-	-	1.48	1.84	0	0	
CV (%)	0	0	0	0	3.85	-	16.82	-	-	0	-	-	-	3.41	5.5	0	0	
Kigalia africana	96.4	3	78.4	-	13.85	-	18	-	-	-	-	-	-	38.4	29.6	0.9	1	Njidda, 2011
Average	96.4	3	78.4	-	13.85	-	18	-	-	-	-	-	-	38.4	29.6	0.9	1	Njidda, 2011 (n=1)
Minimum	96.4	3	78.4	-	13.85	-	18	-	-	-	-	-	-	38.4	29.6	0.9	1	
Maximum	96.4	3	78.4	-	13.85	-	18	-	-	-	-	-	-	38.4	29.6	0.9	1	
Standard deviation	0	0	0	-	0	-	0	-	-	-	-	-	-	0	0	0	0	
CV (%)	0	0	0	-	0	-	0	-	-	-	-	-	-	0	0	0	0	
Leptadenia lancifolia	95.8	4	77.8	-	16.65	-	18	-	-	-	-	-	-	41.2	31.7	1.05	0.3125	Njidda, 2011
Average	95.8	4	77.8	-	16.65	-	18	-	-	-	-	-	-	41.2	31.7	1.05	0.31	Njidda, 2011 (n=1)
Minimum	95.8	4	77.8	-	16.65	-	18	-	-	-	-	-	-	41.2	31.7	1.05	0.31	
Maximum	95.8	4	77.8	-	16.65	-	18	-	-	-	-	-	-	41.2	31.7	1.05	0.31	
Standard deviation	0	0	0	-	0	-	0	-	-	-	-	-	-	0	0	0	0	
CV (%)	0	0	0	-	0	-	0	-	-	-	-	-	-	0	0	0	0	

Leptochloa caerulescens	91.33	1.27	91.09	54.28	5.51	-	8.91	-	-	5.33	-	-	-	78.88	46.77	-	-	Koura et al., 2022
Average	91.33	1.27	91.09	54.28	5.51	-	8.91	-	-	5.33	-	-	-	78.88	46.77	-	-	Koura et al., 2022 (n=1)
Minimum	91.33	1.27	91.09	54.28	5.51	-	8.91	-	-	5.33	-	-	-	78.88	46.77	-	-	
Maximum	91.33	1.27	91.09	54.28	5.51	-	8.91	-	-	5.33	-	-	-	78.88	46.77	-	-	
Standard deviation	0	0	0	0	0	-	0	-	-	0	-	-	-	0	0	-	-	
CV (%)	0	0	0	0	0	-	0	-	-	0	-	-	-	0	0	-	-	
Leucaena leucocephala	-	-	-	-	18.6	-	-	-	-	-	-	-	-	49.8	34.3	-	-	Anyanwu and Etela, 2013
Leucaena leucocephala	-	3.14	95.98	41.96	29.44	-	4.02	-	-	-	-	-	-	58.26	36.48	-	-	Koura et al., 2021
Leucaena leucocephala	67	-	89.84	-	27.11	-	10.16	-	-	-	-	-	-	30.04	17.56	-	-	Idan et al., 2023
Leucaena leucocephala	-	-	91.5	-	30.1	14.7	8.5	21.4	-	-	-	-	-	49.9	30.6	-	-	Osakwe and Steingass, 2006
Average	67	3.14	92.44	41.96	26.31	14.7	7.56	21.4	-	-	-	-	-	47	29.73	-	-	Anyanwu and Etela, 2013; Koura et al., 2021; Idan et al., 2023; Osakwe and Steingass, 2006 (n=4)
Minimum	67	3.14	89.84	41.96	18.6	14.7	4.02	21.4	-	-	-	-	-	30.04	17.56	-	-	
Maximum	67	3.14	95.98	41.96	30.1	14.7	10.16	21.4	-	-	-	-	-	58.26	36.48	-	-	
Standard deviation	0	0	3.18	0	5.3	0	3.18	0	-	-	-	-	-	11.98	8.47	-	-	
CV (%)	0	0	3.44	0	20.14	0	42.01	0	-	-	-	-	-	25.49	28.49	-	-	
Leucaena leucocephala leaves	-	-	92.7	-	24.7	-	7.3	18.1	-	-	-	-	-	32.2	19.5	0.23	0.31	Oduguwa et al., 2013
Average	-	-	92.7	-	24.7	-	7.3	18.1	-	-	-	-	-	32.2	19.5	0.23	0.31	Oduguwa et al., 2013 (n=1)
Minimum	-	-	92.7	-	24.7	-	7.3	18.1	-	-	-	-	-	32.2	19.5	0.23	0.31	
Maximum	-	-	92.7	-	24.7	-	7.3	18.1	-	-	-	-	-	32.2	19.5	0.23	0.31	
Standard deviation	-	-	0	-	0	-	0	0	-	-	-	-	-	0	0	0	0	
CV (%)	-	-	0	-	0	-	0	0	-	-	-	-	-	0	0	0	0	
Leuceana leucocephala	-	-	-	-	18.6	-	-	-	-	-	-	-	-	49.8	34.3	-	-	Anyanwu and Etela, 2013
Leuceana leucocephala	34.2	-	90.5	-	28.7	10.5	9.5	-	-	-	19.3	-	-	-	-	-	-	Idrissou et al., 2017
Leuceana leucocephala	-	-	95.98	-	29.44	-	4.02	-	-	-	-	-	-	58.26	36.48	-	-	Koura et al., 2021
Leuceana leucocephala	-	-	92.7	-	24.7	-	7.3	18.09	-	-	-	-	-	32.2	19.5	-	-	Oduguwa et al., 2013
Leuceana leucocephala	94.3223	3.8061	-	-	-	7.9676	7.5205	-	-	-	-	-	-	-	-	-	-	Mawussi et al., 2022
Average	64.26	3.81	93.06	-	25.36	9.23	7.09	18.09	-	-	19.3	-	-	46.75	30.09	-	-	Anyanwu and Etela, 2013; Idrissou et al., 2017; Koura et al., 2021; Oduguwa et al., 2013; Mawussi et al., 2022 (n=5)
Minimum	34.2	3.81	90.5	-	18.6	7.97	4.02	18.09	-	-	19.3	-	-	32.2	19.5	-	-	
Maximum	94.32	3.81	95.98	-	29.44	10.5	9.5	18.09	-	-	19.3	-	-	58.26	36.48	-	-	
Standard deviation	42.51	0	2.76	-	4.96	1.79	2.27	0	-	-	0	-	-	13.29	9.24	-	-	
CV (%)	66.16	0	2.96	-	19.58	19.39	32.04	0	-	-	0	-	-	28.44	30.7	-	-	
Lonchocarpus sericeus	-	-	-	-	12.5	-	-	-	-	-	-	-	-	77.1	66.1	-	-	Anyanwu and Etela, 2013
Average	-	-	-	-	12.5	-	-	-	-	-	-	-	-	77.1	66.1	-	-	Anyanwu and Etela, 2013 (n=1)
Minimum	-	-	-	-	12.5	-	-	-	-	-	-	-	-	77.1	66.1	-	-	
Maximum	-	-	-	-	12.5	-	-	-	-	-	-	-	-	77.1	66.1	-	-	
Standard deviation	-	-	-	-	0	-	-	-	-	-	-	-	-	0	0	-	-	
CV (%)	-	-	-	-	0	-	-	-	-	-	-	-	-	0	0	-	-	
Loudetia aroundinacea	91.06	1.04	89.16	51.38	6.53	-	10.84	-	-	5.25	-	-	-	73.88	51.24	-	-	Koura et al., 2022
Average	91.06	1.04	89.16	51.38	6.53	-	10.84	-	-	5.25	-	-	-	73.88	51.24	-	-	Koura et al., 2022 (n=1)

Minimum	91.06	1.04	89.16	51.38	6.53	-	10.84	-	-	5.25	-	-	-	73.88	51.24	-	-	
Maximum	91.06	1.04	89.16	51.38	6.53	-	10.84	-	-	5.25	-	-	-	73.88	51.24	-	-	
Standard deviation	0	0	0	0	0	-	0	-	-	0	-	-	-	0	0	-	-	
CV (%)	0	0	0	0	0	-	0	-	-	0	-	-	-	0	0	-	-	
Mallotus oppositifolius	-	5.12	90.76	55.28	16.37	-	9.24	-	-	-	-	-	-	41.07	30.89	-	-	Koura et al., 2021
Average	-	5.12	90.76	55.28	16.37	-	9.24	-	-	-	-	-	-	41.07	30.89	-	-	Koura et al., 2021 (n=1)
Minimum	-	5.12	90.76	55.28	16.37	-	9.24	-	-	-	-	-	-	41.07	30.89	-	-	
Maximum	-	5.12	90.76	55.28	16.37	-	9.24	-	-	-	-	-	-	41.07	30.89	-	-	
Standard deviation	-	0	0	0	0	-	0	-	-	-	-	-	-	0	0	-	-	
CV (%)	-	0	0	0	0	-	0	-	-	-	-	-	-	0	0	-	-	
Mangifera indica	-	3.27	92.9	41.99	9.38	-	7.1	-	-	-	-	-	-	53.23	38.8	-	-	Koura et al., 2021 (GCZ)
Mangifera indica	-	3.97	87.04	43.52	9.18	-	12.96	-	-	-	-	-	-	44.42	36.9	-	-	Koura et al., 2021 (SGZ)
Mangifera indica	90.6	2.73	94.3	-	11.5	24.6	5.73	-	-	-	-	-	-	46.6	36.9	-	-	Sasu et al., 2023
Mangifera indica	94.1063	3.1958	-	-	-	19.3168	8.965	-	-	-	-	-	-	-	-	-	-	Mawussi et al., 2022
Average	92.35	3.29	91.41	42.76	10.02	21.96	8.69	-	-	-	-	-	-	48.08	37.53	-	-	Koura et al., 2021 (GCZ); Koura et al., 2021 (SGZ); Sasu et al., 2023; Mawussi et al., 2022 (n=4)
Minimum	90.6	2.73	87.04	41.99	9.18	19.32	5.73	-	-	-	-	-	-	44.42	36.9	-	-	
Maximum	94.11	3.97	94.3	43.52	11.5	24.6	12.96	-	-	-	-	-	-	53.23	38.8	-	-	
Standard deviation	2.48	0.51	3.85	1.08	1.29	3.74	3.14	-	-	-	-	-	-	4.59	1.1	-	-	
CV (%)	2.68	15.54	4.21	2.53	12.83	17.01	36.15	-	-	-	-	-	-	9.54	2.92	-	-	
Manguifera indica (from the Guineo-Congolian zone of Benin)	-	-	92.9	-	9.38	-	7.1	-	-	-	-	-	-	53.23	38.8	-	-	Koura et al., 2021
Average	-	-	92.9	-	9.38	-	7.1	-	-	-	-	-	-	53.23	38.8	-	-	Koura et al., 2021 (n=1)
Minimum	-	-	92.9	-	9.38	-	7.1	-	-	-	-	-	-	53.23	38.8	-	-	
Maximum	-	-	92.9	-	9.38	-	7.1	-	-	-	-	-	-	53.23	38.8	-	-	
Standard deviation	-	-	0	-	0	-	0	-	-	-	-	-	-	0	0	-	-	
CV (%)	-	-	0	-	0	-	0	-	-	-	-	-	-	0	0	-	-	
Manguifera indica L. (Leaves)	-	-	87.04	-	9.18	-	12.96	-	-	-	-	-	-	44.42	36.9	-	-	Koura et al., 2021
Manguifera indica L. (Leaves)	-	-	-	45.89	8.2	-	8.6	-	-	6.52	-	-	-	45	38.9	1.69	1.5	Amole et al., 2022
Average	-	-	87.04	45.89	8.69	-	10.78	-	-	6.52	-	-	-	44.71	37.9	1.69	1.5	Koura et al., 2021; Amole et al., 2022 (n=2)
Minimum	-	-	87.04	45.89	8.2	-	8.6	-	-	6.52	-	-	-	44.42	36.9	1.69	1.5	
Maximum	-	-	87.04	45.89	9.18	-	12.96	-	-	6.52	-	-	-	45	38.9	1.69	1.5	
Standard deviation	-	-	0	0	0.69	-	3.08	-	-	0	-	-	-	0.41	1.41	0	0	
CV (%)	-	-	0	0	7.97	-	28.6	-	-	0	-	-	-	0.92	3.73	0	0	
Manihot esculenta	-	-	92.14	-	25.38	-	7.86	-	-	-	-	-	-	-	-	-	-	Koura et al., 2021
Manihot esculenta	-	6.88	92.17	46.12	29.85	-	7.83	-	-	-	-	-	-	50.1	39.56	-	-	Koura et al., 2021 (GCZ)
Manihot esculenta	-	14.9	92.14	52.33	25.38	-	7.86	-	-	-	-	-	-	33.93	25.02	-	-	Koura et al., 2021 (SGZ)
Manihot esculenta	95.6112	6.1415	-	-	-	8.8719	8.0202	-	-	-	-	-	-	-	-	-	-	Mawussi et al., 2022

Average	95.61	9.31	92.15	49.22	26.87	8.87	7.89	-	-	-	-	-	-	42.02	32.29	-	-	Koura et al., 2021; Koura et al., 2021 (GCZ); Koura et al., 2021 (SGZ); Mawussi et al., 2022 (n=4)
Minimum	95.61	6.14	92.14	46.12	25.38	8.87	7.83	-	-	-	-	-	-	33.93	25.02	-	-	
Maximum	95.61	14.9	92.17	52.33	29.85	8.87	8.02	-	-	-	-	-	-	50.1	39.56	-	-	
Standard deviation	0	4.86	0.02	4.39	2.58	0	0.09	-	-	-	-	-	-	11.43	10.28	-	-	
CV (%)	0	52.19	0.02	8.92	9.6	0	1.09	-	-	-	-	-	-	27.21	31.84	-	-	
Manihot esculenta (from the Guineo-Congolian zone of Benin)	-	-	92.17	-	29.85	-	7.83	-	-	-	-	0.5	-	-	-	-	-	Koura et al., 2021
Average	-	-	92.17	-	29.85	-	7.83	-	-	-	-	0.5	-	-	-	-	-	Koura et al., 2021 (n=1)
Minimum	-	-	92.17	-	29.85	-	7.83	-	-	-	-	0.5	-	-	-	-	-	
Maximum	-	-	92.17	-	29.85	-	7.83	-	-	-	-	0.5	-	-	-	-	-	
Standard deviation	-	-	0	-	0	-	0	-	-	-	-	0	-	-	-	-	-	
CV (%)	-	-	0	-	0	-	0	-	-	-	-	0	-	-	-	-	-	
Milletia griffoneanus	-	-	-	-	14.5	-	-	-	-	-	-	-	-	62.7	53.5	-	-	Anyanwu and Etela, 2013
Average	-	-	-	-	14.5	-	-	-	-	-	-	-	-	62.7	53.5	-	-	Anyanwu and Etela, 2013 (n=1)
Minimum	-	-	-	-	14.5	-	-	-	-	-	-	-	-	62.7	53.5	-	-	
Maximum	-	-	-	-	14.5	-	-	-	-	-	-	-	-	62.7	53.5	-	-	
Standard deviation	-	-	-	-	0	-	-	-	-	-	-	-	-	0	0	-	-	
CV (%)	-	-	-	-	0	-	-	-	-	-	-	-	-	0	0	-	-	
Milletia thoningii	-	-	-	-	10.7	-	-	-	-	-	-	-	-	63.7	50.8	-	-	Anyanwu and Etela, 2013
Average	-	-	-	-	10.7	-	-	-	-	-	-	-	-	63.7	50.8	-	-	Anyanwu and Etela, 2013 (n=1)
Minimum	-	-	-	-	10.7	-	-	-	-	-	-	-	-	63.7	50.8	-	-	
Maximum	-	-	-	-	10.7	-	-	-	-	-	-	-	-	63.7	50.8	-	-	
Standard deviation	-	-	-	-	0	-	-	-	-	-	-	-	-	0	0	-	-	
CV (%)	-	-	-	-	0	-	-	-	-	-	-	-	-	0	0	-	-	
Millettia thonningii	-	3.12	91.71	33.41	21.04	-	8.29	-	-	-	-	-	-	60.52	38.91	-	-	Koura et al., 2021
Average	-	3.12	91.71	33.41	21.04	-	8.29	-	-	-	-	-	-	60.52	38.91	-	-	Koura et al., 2021 (n=1)
Minimum	-	3.12	91.71	33.41	21.04	-	8.29	-	-	-	-	-	-	60.52	38.91	-	-	
Maximum	-	3.12	91.71	33.41	21.04	-	8.29	-	-	-	-	-	-	60.52	38.91	-	-	
Standard deviation	-	0	0	0	0	-	0	-	-	-	-	-	-	0	0	-	-	
CV (%)	-	0	0	0	0	-	0	-	-	-	-	-	-	0	0	-	-	
Morinda lucida	-	6.89	91.26	58.54	16.2	-	8.74	-	-	-	-	-	-	46.76	34.94	-	-	Koura et al., 2021
Average	-	6.89	91.26	58.54	16.2	-	8.74	-	-	-	-	-	-	46.76	34.94	-	-	Koura et al., 2021 (n=1)
Minimum	-	6.89	91.26	58.54	16.2	-	8.74	-	-	-	-	-	-	46.76	34.94	-	-	
Maximum	-	6.89	91.26	58.54	16.2	-	8.74	-	-	-	-	-	-	46.76	34.94	-	-	
Standard deviation	-	0	0	0	0	-	0	-	-	-	-	-	-	0	0	-	-	
CV (%)	-	0	0	0	0	-	0	-	-	-	-	-	-	0	0	-	-	
Moringa oleifera	-	6.02	85.79	52.26	41.39	-	14.21	-	-	-	-	-	-	36.71	27.87	-	-	Koura et al., 2021 (GCZ)
Moringa oleifera	-	10.1	89.93	70.74	26.2	-	10.07	-	-	-	-	-	-	30.65	18.38	-	-	Koura et al., 2021 (SGZ)
Moringa oleifera	88	3.43	85	-	31.1	13.6	15	-	-	-	-	-	-	45.7	34.3	-	-	Sasu et al., 2023
Moringa oleifera	93.0571	4.038	-	-	-	7.5468	7.4464	-	-	-	-	-	-	-	-	-	-	Mawussi et al., 2022

Average	90.53	5.9	86.91	61.5	32.9	10.57	11.68	-	-	-	-	-	-	37.69	26.85	-	-	Koura et al., 2021 (GCZ); Koura et al., 2021 (SGZ); Sasu et al., 2023; Mawussi et al., 2022 (n=4)
Minimum	88	3.43	85	52.26	26.2	7.55	7.45	-	-	-	-	-	-	30.65	18.38	-	-	
Maximum	93.06	10.1	89.93	70.74	41.39	13.6	15	-	-	-	-	-	-	45.7	34.3	-	-	
Standard deviation	3.58	3.01	2.65	13.07	7.75	4.28	3.56	-	-	-	-	-	-	7.57	8.01	-	-	
CV (%)	3.95	51.08	3.05	21.25	23.57	40.48	30.44	-	-	-	-	-	-	20.09	29.83	-	-	
Moringa oleifera (from the Guineo-Congolian zone of Benin)	-	-	85.79	-	41.39	-	14.21	-	-	-	-	-	-	-	-	-	-	Koura et al., 2021
Average	-	-	85.79	-	41.39	-	14.21	-	-	-	-	-	-	-	-	-	-	Koura et al., 2021 (n=1)
Minimum	-	-	85.79	-	41.39	-	14.21	-	-	-	-	-	-	-	-	-	-	
Maximum	-	-	85.79	-	41.39	-	14.21	-	-	-	-	-	-	-	-	-	-	
Standard deviation	-	-	0	-	0	-	0	-	-	-	-	-	-	-	-	-	-	
CV (%)	-	-	0	-	0	-	0	-	-	-	-	-	-	-	-	-	-	
Moringa oleifera (leaves)	-	-	89.93	-	26.2	-	10.07	-	-	-	-	-	1.84	-	-	-	-	Koura et al., 2021
Average	-	-	89.93	-	26.2	-	10.07	-	-	-	-	-	1.84	-	-	-	-	Koura et al., 2021 (n=1)
Minimum	-	-	89.93	-	26.2	-	10.07	-	-	-	-	-	1.84	-	-	-	-	
Maximum	-	-	89.93	-	26.2	-	10.07	-	-	-	-	-	1.84	-	-	-	-	
Standard deviation	-	-	0	-	0	-	0	-	-	-	-	-	0	-	-	-	-	
CV (%)	-	-	0	-	0	-	0	-	-	-	-	-	0	-	-	-	-	
Moringa oleifera residues																		
Average	-	-	-	-	-	-	-	-	-	-	-	-	-	-	-	-	-	
Minimum	-	-	-	-	-	-	-	-	-	-	-	-	-	-	-	-	-	
Maximum	-	-	-	-	-	-	-	-	-	-	-	-	-	-	-	-	-	
Standard deviation	-	-	-	-	-	-	-	-	-	-	-	-	-	-	-	-	-	
CV (%)	-	-	-	-	-	-	-	-	-	-	-	-	-	-	-	-	-	
Musa sapientum	-	8.57	87.38	27.66	18.61	-	12.62	-	-	-	-	-	-	69.97	36.26	-	-	Koura et al., 2021 (GCZ)
Musa sapientum	-	15.33	86.57	32.27	13.86	-	13.43	-	-	-	-	-	-	50.86	13.87	-	-	Koura et al., 2021 (SGZ)
Average	-	11.95	86.97	29.97	16.23	-	13.02	-	-	-	-	-	-	60.41	25.06	-	-	Koura et al., 2021 (GCZ); Koura et al., 2021 (SGZ) (n=2)
Minimum	-	8.57	86.57	27.66	13.86	-	12.62	-	-	-	-	-	-	50.86	13.87	-	-	
Maximum	-	15.33	87.38	32.27	18.61	-	13.43	-	-	-	-	-	-	69.97	36.26	-	-	
Standard deviation	-	4.78	0.57	3.26	3.36	-	0.57	-	-	-	-	-	-	13.51	15.83	-	-	
CV (%)	-	40	0.66	10.88	20.69	-	4.4	-	-	-	-	-	-	22.37	63.16	-	-	
Musa sapientum (from the Sudano-Guinean zone of Benin)	-	-	86.57	-	13.86	-	13.43	-	-	-	-	-	-	50.86	13.87	-	-	Koura et al., 2021
Average	-	-	86.57	-	13.86	-	13.43	-	-	-	-	-	-	50.86	13.87	-	-	Koura et al., 2021 (n=1)
Minimum	-	-	86.57	-	13.86	-	13.43	-	-	-	-	-	-	50.86	13.87	-	-	
Maximum	-	-	86.57	-	13.86	-	13.43	-	-	-	-	-	-	50.86	13.87	-	-	
Standard deviation	-	-	0	-	0	-	0	-	-	-	-	-	-	0	0	-	-	
CV (%)	-	-	0	-	0	-	0	-	-	-	-	-	-	0	0	-	-	

Musa sapientum – (from the Guineo-Congolian zone of Benin)	-	-	87.38	-	18.61	-	12.62	-	-	-	-	-	-	69.97	36.26	-	-	Koura et al., 2021
Average	-	-	87.38	-	18.61	-	12.62	-	-	-	-	-	-	69.97	36.26	-	-	Koura et al., 2021 (n=1)
Minimum	-	-	87.38	-	18.61	-	12.62	-	-	-	-	-	-	69.97	36.26	-	-	
Maximum	-	-	87.38	-	18.61	-	12.62	-	-	-	-	-	-	69.97	36.26	-	-	
Standard deviation	-	-	0	-	0	-	0	-	-	-	-	-	-	0	0	-	-	
CV (%)	-	-	0	-	0	-	0	-	-	-	-	-	-	0	0	-	-	
Newbouldia laevis	-	8.11	91.39	25.77	15.98	-	8.61	-	-	-	-	-	-	67.22	44.08	-	-	Koura et al., 2021
Average	-	8.11	91.39	25.77	15.98	-	8.61	-	-	-	-	-	-	67.22	44.08	-	-	Koura et al., 2021 (n=1)
Minimum	-	8.11	91.39	25.77	15.98	-	8.61	-	-	-	-	-	-	67.22	44.08	-	-	
Maximum	-	8.11	91.39	25.77	15.98	-	8.61	-	-	-	-	-	-	67.22	44.08	-	-	
Standard deviation	-	0	0	0	0	-	0	-	-	-	-	-	-	0	0	-	-	
CV (%)	-	0	0	0	0	-	0	-	-	-	-	-	-	0	0	-	-	
Newbouldia lavis	-	-	91.39	-	15.98	-	8.61	-	-	-	-	-	-	67.22	44.08	-	-	Koura et al., 2021
Average	-	-	91.39	-	15.98	-	8.61	-	-	-	-	-	-	67.22	44.08	-	-	Koura et al., 2021 (n=1)
Minimum	-	-	91.39	-	15.98	-	8.61	-	-	-	-	-	-	67.22	44.08	-	-	
Maximum	-	-	91.39	-	15.98	-	8.61	-	-	-	-	-	-	67.22	44.08	-	-	
Standard deviation	-	-	0	-	0	-	0	-	-	-	-	-	-	0	0	-	-	
CV (%)	-	-	0	-	0	-	0	-	-	-	-	-	-	0	0	-	-	
Oryza barthii	93.07	-	78.34	-	9.19	-	21.66	-	-	-	-	0.55	-	-	-	-	-	Sanou et al., 2016
Average	93.07	-	78.34	-	9.19	-	21.66	-	-	-	-	0.55	-	-	-	-	-	Sanou et al., 2016 (n=1)
Minimum	93.07	-	78.34	-	9.19	-	21.66	-	-	-	-	0.55	-	-	-	-	-	
Maximum	93.07	-	78.34	-	9.19	-	21.66	-	-	-	-	0.55	-	-	-	-	-	
Standard deviation	0	-	0	-	0	-	0	-	-	-	-	0	-	-	-	-	-	
CV (%)	0	-	0	-	0	-	0	-	-	-	-	0	-	-	-	-	-	
Oxytenanthera abyssinica	91.8	4.05	85.92	36.55	12.8	26.9	14.08	-	-	3.93	-	-	-	51.43	33.43	-	-	Sasu et al., 2023
Average	91.8	4.05	85.92	36.55	12.8	26.9	14.08	-	-	3.93	-	-	-	51.43	33.43	-	-	Sasu et al., 2023 (n=1)
Minimum	91.8	4.05	85.92	36.55	12.8	26.9	14.08	-	-	3.93	-	-	-	51.43	33.43	-	-	
Maximum	91.8	4.05	85.92	36.55	12.8	26.9	14.08	-	-	3.93	-	-	-	51.43	33.43	-	-	
Standard deviation	0	0	0	0	0	0	0	-	-	0	-	-	-	0	0	-	-	
CV (%)	0	0	0	0	0	0	0	-	-	0	-	-	-	0	0	-	-	
Megathyrsus maximus	89.47	-	91.12	-	5.37	49.24	8.88	-	-	-	-	-	-	-	-	-	-	Sasu et al., 2023
Average	89.47	-	91.12	-	5.37	49.24	8.88	-	-	-	-	-	-	-	-	-	-	Sasu et al., 2023 (n=1)
Minimum	89.47	-	91.12	-	5.37	49.24	8.88	-	-	-	-	-	-	-	-	-	-	
Maximum	89.47	-	91.12	-	5.37	49.24	8.88	-	-	-	-	-	-	-	-	-	-	
Standard deviation	0	-	0	-	0	0	0	-	-	-	-	-	-	-	-	-	-	
CV (%)	0	-	0	-	0	0	0	-	-	-	-	-	-	-	-	-	-	
Panicum maximum	92.08	-	90.1	58.63	6.45	-	9.9	-	-	-	-	0.09	0.11	-	-	-	-	Gbenou et al., 2020
Panicum maximum	42.7	-	90.1	-	10.1	31.3	9.9	-	-	-	-	-	-	-	-	-	-	Idrissou et al., 2017
Panicum maximum	89.67	1.87	86.91	-	12.26	17.89	13.09	13.1796	-	-	-	-	-	69.87	41.36	-	-	Lamidi and Ogunkunle, 2015

Panicum maximum	29	-	91.2	-	4.63	-	8.9	-	-	-	-	-	-	77.4	-	-	-	Etela et al., 2008
Panicum maximum	61.2	1.39	91.33	-	9.03	32.8	8.67	16.45	-	-	-	-	-	-	-	-	-	Okagbare et al., 2004
Panicum maximum	83.63	2.4	90.82	-	12.33	-	9.18	-	-	13.131484	-	-	-	-	-	-	-	Kouadio et al., 2024
Panicum maximum	-	-	-	-	9.56	14	-	-	-	-	-	-	-	-	-	0.047	0.033	Amegnaglo et al., 2018
Average	66.38	1.89	90.08	58.63	9.19	24	9.94	14.81	-	13.13	-	0.09	0.11	73.64	41.36	0.05	0.03	Gbenou et al., 2020; Idrissou et al., 2017; Lamidi and Ogunkunle, 2015; Etela et al., 2008; Okagbare et al., 2004; Kouadio et al., 2024; Amegnaglo et al., 2018 (n=7)
Minimum	29	1.39	86.91	58.63	4.63	14	8.67	13.18	-	13.13	-	0.09	0.11	69.87	41.36	0.05	0.03	
Maximum	92.08	2.4	91.33	58.63	12.33	32.8	13.09	16.45	-	13.13	-	0.09	0.11	77.4	41.36	0.05	0.03	
Standard deviation	26.4	0.51	1.64	0	2.85	9.45	1.62	2.31	-	0	-	0	0	5.32	0	0	0	
CV (%)	39.77	26.78	1.82	0	30.95	39.39	16.34	15.61	-	0	-	0	0	7.23	0	0	0	
Parkia bicolor	-	-	-	-	10.4	-	-	-	-	-	-	-	-	66.1	59.1	-	-	Anyanwu and Etela, 2013
Average	-	-	-	-	10.4	-	-	-	-	-	-	-	-	66.1	59.1	-	-	Anyanwu and Etela, 2013 (n=1)
Minimum	-	-	-	-	10.4	-	-	-	-	-	-	-	-	66.1	59.1	-	-	
Maximum	-	-	-	-	10.4	-	-	-	-	-	-	-	-	66.1	59.1	-	-	
Standard deviation	-	-	-	-	0	-	-	-	-	-	-	-	-	0	0	-	-	
CV (%)	-	-	-	-	0	-	-	-	-	-	-	-	-	0	0	-	-	
Paspalum notatum	91.36	1.43	93.17	52.35	8.42	-	6.83	-	-	6.62	-	-	-	71.19	41.95	-	-	Koura et al., 2022
Average	91.36	1.43	93.17	52.35	8.42	-	6.83	-	-	6.62	-	-	-	71.19	41.95	-	-	Koura et al., 2022 (n=1)
Minimum	91.36	1.43	93.17	52.35	8.42	-	6.83	-	-	6.62	-	-	-	71.19	41.95	-	-	
Maximum	91.36	1.43	93.17	52.35	8.42	-	6.83	-	-	6.62	-	-	-	71.19	41.95	-	-	
Standard deviation	0	0	0	0	0	-	0	-	-	0	-	-	-	0	0	-	-	
CV (%)	0	0	0	0	0	-	0	-	-	0	-	-	-	0	0	-	-	
Paspalum vaginatum	90.26	1.4	86.88	66.87	6.09	-	13.12	-	-	5.6	-	-	-	73.93	47.55	-	-	Koura et al., 2022
Average	90.26	1.4	86.88	66.87	6.09	-	13.12	-	-	5.6	-	-	-	73.93	47.55	-	-	Koura et al., 2022 (n=1)
Minimum	90.26	1.4	86.88	66.87	6.09	-	13.12	-	-	5.6	-	-	-	73.93	47.55	-	-	
Maximum	90.26	1.4	86.88	66.87	6.09	-	13.12	-	-	5.6	-	-	-	73.93	47.55	-	-	
Standard deviation	0	0	0	0	0	-	0	-	-	0	-	-	-	0	0	-	-	
CV (%)	0	0	0	0	0	-	0	-	-	0	-	-	-	0	0	-	-	
Pennisetum glaucum	93.59	-	94.73	-	1.13	-	5.27	-	-	-	-	0.73	0.57	-	-	-	-	Sanou et al., 2016
Average	93.59	-	94.73	-	1.13	-	5.27	-	-	-	-	0.73	0.57	-	-	-	-	Sanou et al., 2016 (n=1)
Minimum	93.59	-	94.73	-	1.13	-	5.27	-	-	-	-	0.73	0.57	-	-	-	-	
Maximum	93.59	-	94.73	-	1.13	-	5.27	-	-	-	-	0.73	0.57	-	-	-	-	
Standard deviation	0	-	0	-	0	-	0	-	-	-	-	0	0	-	-	-	-	
CV (%)	0	-	0	-	0	-	0	-	-	-	-	0	0	-	-	-	-	
Pennisetum pedicellatum	93.41	-	80.16	-	9.24	-	19.84	-	-	7.46	-	-	-	59.48	46.69	-	-	Sissao et al., 2024
Pennisetum pedicellatum	96.5	2.5	-	-	4.9	14.5	7.5	-	-	-	-	-	-	-	-	0.65	4.28	Muftau and Musa, 2020
Average	94.95	2.5	80.16	-	7.07	14.5	13.67	-	-	7.46	-	-	-	59.48	46.69	0.65	4.28	Sissao et al., 2024; Muftau and Musa, 2020 (n=2)
Minimum	93.41	2.5	80.16	-	4.9	14.5	7.5	-	-	7.46	-	-	-	59.48	46.69	0.65	4.28	

Maximum	96.5	2.5	80.16	-	9.24	14.5	19.84	-	-	7.46	-	-	-	59.48	46.69	0.65	4.28	
Standard deviation	2.18	0	0	-	3.07	0	8.73	-	-	0	-	-	-	0	0	0	0	
CV (%)	2.3	0	0	-	43.41	0	63.83	-	-	0	-	-	-	0	0	0	0	
Pennisetum purpureum	91.11	1.47	92.21	48.92	4.62	-	7.79	-	-	4.32	-	-	-	78.45	51.27	-	-	Koura et al., 2022
Pennisetum purpureum	81.2	0.09	91.74	-	9.91	35.1	8.26	24.02	-	-	-	-	-	-	-	-	-	Okagbare et al., 2004
Average	86.16	0.78	91.97	48.92	7.27	35.1	8.03	24.02	-	4.32	-	-	-	78.45	51.27	-	-	Koura et al., 2022; Okagbare et al., 2004 (n=2)
Minimum	81.2	0.09	91.74	48.92	4.62	35.1	7.79	24.02	-	4.32	-	-	-	78.45	51.27	-	-	
Maximum	91.11	1.47	92.21	48.92	9.91	35.1	8.26	24.02	-	4.32	-	-	-	78.45	51.27	-	-	
Standard deviation	7.01	0.98	0.33	0	3.74	0	0.33	0	-	0	-	-	-	0	0	-	-	
CV (%)	8.13	125.1	0.36	0	51.49	0	4.14	0	-	0	-	-	-	0	0	-	-	
Pennisetum purpureum (leaves)	91.11	-	92.21	48.92	4.62	-	7.79	-	-	4.32	-	-	-	78.45	51.27	-	-	Koura et al., 2022
Average	91.11	-	92.21	48.92	4.62	-	7.79	-	-	4.32	-	-	-	78.45	51.27	-	-	Koura et al., 2022 (n=1)
Minimum	91.11	-	92.21	48.92	4.62	-	7.79	-	-	4.32	-	-	-	78.45	51.27	-	-	
Maximum	91.11	-	92.21	48.92	4.62	-	7.79	-	-	4.32	-	-	-	78.45	51.27	-	-	
Standard deviation	0	-	0	0	0	-	0	-	-	0	-	-	-	0	0	-	-	
CV (%)	0	-	0	0	0	-	0	-	-	0	-	-	-	0	0	-	-	
Pericopsis laxiflora	-	-	-	-	-	-	-	-	-	-	-	-	-	-	-	-	-	-
Average	-	-	-	-	-	-	-	-	-	-	-	-	-	-	-	-	-	- (n=1)
Minimum	-	-	-	-	-	-	-	-	-	-	-	-	-	-	-	-	-	
Maximum	-	-	-	-	-	-	-	-	-	-	-	-	-	-	-	-	-	
Standard deviation	-	-	-	-	-	-	-	-	-	-	-	-	-	-	-	-	-	
CV (%)	-	-	-	-	-	-	-	-	-	-	-	-	-	-	-	-	-	
Persea americana	-	5.91	93.51	13.21	14.09	-	6.49	-	-	-	-	-	-	60.95	51.63	-	-	Koura et al., 2021
Average	-	5.91	93.51	13.21	14.09	-	6.49	-	-	-	-	-	-	60.95	51.63	-	-	Koura et al., 2021 (n=1)
Minimum	-	5.91	93.51	13.21	14.09	-	6.49	-	-	-	-	-	-	60.95	51.63	-	-	
Maximum	-	5.91	93.51	13.21	14.09	-	6.49	-	-	-	-	-	-	60.95	51.63	-	-	
Standard deviation	-	0	0	0	0	-	0	-	-	-	-	-	-	0	0	-	-	
CV (%)	-	0	0	0	0	-	0	-	-	-	-	-	-	0	0	-	-	
Phyllanthus discoideus	-	7.29	93.78	61.51	15.45	-	6.22	-	-	-	-	-	-	41.51	20.79	-	-	Koura et al., 2021
Average	-	7.29	93.78	61.51	15.45	-	6.22	-	-	-	-	-	-	41.51	20.79	-	-	Koura et al., 2021 (n=1)
Minimum	-	7.29	93.78	61.51	15.45	-	6.22	-	-	-	-	-	-	41.51	20.79	-	-	
Maximum	-	7.29	93.78	61.51	15.45	-	6.22	-	-	-	-	-	-	41.51	20.79	-	-	
Standard deviation	-	0	0	0	0	-	0	-	-	-	-	-	-	0	0	-	-	
CV (%)	-	0	0	0	0	-	0	-	-	-	-	-	-	0	0	-	-	
Piliostigma thonningii	-	3.89	82.84	-	14.75	-	17.16	-	-	-	-	-	-	58.94	23.38	0.82	0.38	Isah et al., 2015
Piliostigma thonningii	57.7	-	96.9	36	10.7	26.6	-	-	-	-	64	0.46	0.34	60.7	60	-	-	Sidiimorou et al., 2016
Piliostigma thonningii	94.4373	5.41	-	-	-	19.1636	4.9345	-	-	-	-	-	-	-	-	-	-	Mawussi et al., 2022
Average	76.07	4.65	89.87	36	12.72	22.88	11.05	-	-	-	64	0.46	0.34	59.82	41.69	0.82	0.38	Isah et al., 2015; Sidiimorou et al., 2016; Mawussi et al., 2022 (n=3)
Minimum	57.7	3.89	82.84	36	10.7	19.16	4.93	-	-	-	64	0.46	0.34	58.94	23.38	0.82	0.38	
Maximum	94.44	5.41	96.9	36	14.75	26.6	17.16	-	-	-	64	0.46	0.34	60.7	60	0.82	0.38	
Standard deviation	25.98	1.07	9.94	0	2.86	5.26	8.64	-	-	-	0	0	0	1.24	25.89	0	0	

CV (%)	34.15	23.11	11.06	0	22.51	22.98	78.25	-	-	-	0	0	0	2.08	62.11	0	0	
Pouteria alnifolia	-	6.57	90.92	16.07	12.5	-	9.08	-	-	-	-	-	-	55.28	35.54	-	-	Koura et al., 2021
Average	-	6.57	90.92	16.07	12.5	-	9.08	-	-	-	-	-	-	55.28	35.54	-	-	Koura et al., 2021 (n=1)
Minimum	-	6.57	90.92	16.07	12.5	-	9.08	-	-	-	-	-	-	55.28	35.54	-	-	
Maximum	-	6.57	90.92	16.07	12.5	-	9.08	-	-	-	-	-	-	55.28	35.54	-	-	
Standard deviation	-	0	0	0	0	-	0	-	-	-	-	-	-	0	0	-	-	
CV (%)	-	0	0	0	0	-	0	-	-	-	-	-	-	0	0	-	-	
Pterocarpus erinaceus	-	4.16	91.49	43.16	15.69	-	8.51	-	-	-	-	-	-	51.49	33.42	-	-	Koura et al., 2021
Pterocarpus erinaceus	-	4.73	85.39	-	18.42	-	14.61	-	-	-	-	-	-	52.86	38.43	0.44	0.83	Isah et al., 2015
Pterocarpus erinaceus	87.58	6.8	90.58	-	15.51	-	9.42	-	-	14.52082304	-	-	-	-	-	-	-	Kouadio et al., 2024
Pterocarpus erinaceus	27.5	-	96.4	45.7	19.7	28.3	-	-	-	-	148	0.6	0.49	54.6	49.5	-	-	Sidiimorou et al., 2016
Average	57.54	5.23	90.97	44.43	17.33	28.3	10.85	-	-	14.52	148	0.6	0.49	52.98	40.45	0.44	0.83	Koura et al., 2021; Isah et al., 2015; Kouadio et al., 2024; Sidiimorou et al., 2016 (n=4)
Minimum	27.5	4.16	85.39	43.16	15.51	28.3	8.51	-	-	14.52	148	0.6	0.49	51.49	33.42	0.44	0.83	
Maximum	87.58	6.8	96.4	45.7	19.7	28.3	14.61	-	-	14.52	148	0.6	0.49	54.6	49.5	0.44	0.83	
Standard deviation	42.48	1.39	4.51	1.8	2.07	0	3.29	-	-	0	0	0	0	1.56	8.23	0	0	
CV (%)	73.83	26.56	4.96	4.04	11.92	0	30.34	-	-	0	0	0	0	2.94	20.34	0	0	
Pterocarpus erinacieus	37.67	-	91.35	-	10.53	-	8.65	-	-	-	-	0.62	-	-	-	-	-	Avornyo et al., 2020
Pterocarpus erinacieus	-	-	85.39	-	18.42	-	-	-	-	-	-	0.53	-	-	-	-	-	Isah et al., 2015
Pterocarpus erinacieus	-	-	91.49	-	15.69	-	8.51	-	-	-	-	0.51	-	-	-	-	-	Koura et al., 2021
Pterocarpus erinacieus	93.35	-	93.7	-	10.55	-	6.3	-	-	-	-	-	-	-	-	-	-	Sanou et al., 2016
Average	65.51	-	90.48	-	13.8	-	7.82	-	-	-	-	0.55	-	-	-	-	-	Avornyo et al., 2020; Isah et al., 2015; Koura et al., 2021; Sanou et al., 2016 (n=4)
Minimum	37.67	-	85.39	-	10.53	-	6.3	-	-	-	-	0.51	-	-	-	-	-	
Maximum	93.35	-	93.7	-	18.42	-	8.65	-	-	-	-	0.62	-	-	-	-	-	
Standard deviation	39.37	-	3.56	-	3.92	-	1.32	-	-	-	-	0.06	-	-	-	-	-	
CV (%)	60.1	-	3.94	-	28.43	-	16.86	-	-	-	-	10.59	-	-	-	-	-	
Pterocarpus santalinoides	-	-	-	-	13.1	-	-	-	-	-	-	-	-	61.7	51.7	-	-	Anyanwu and Etela, 2013
Average	-	-	-	-	13.1	-	-	-	-	-	-	-	-	61.7	51.7	-	-	Anyanwu and Etela, 2013 (n=1)
Minimum	-	-	-	-	13.1	-	-	-	-	-	-	-	-	61.7	51.7	-	-	
Maximum	-	-	-	-	13.1	-	-	-	-	-	-	-	-	61.7	51.7	-	-	
Standard deviation	-	-	-	-	0	-	-	-	-	-	-	-	-	0	0	-	-	
CV (%)	-	-	-	-	0	-	-	-	-	-	-	-	-	0	0	-	-	
Rottbollia exaltata	94.13	-	87.72	-	6.04	-	12.28	-	-	-	-	-	-	-	-	-	-	Sanou et al., 2016
Average	94.13	-	87.72	-	6.04	-	12.28	-	-	-	-	-	-	-	-	-	-	Sanou et al., 2016 (n=1)
Minimum	94.13	-	87.72	-	6.04	-	12.28	-	-	-	-	-	-	-	-	-	-	
Maximum	94.13	-	87.72	-	6.04	-	12.28	-	-	-	-	-	-	-	-	-	-	
Standard deviation	0	-	0	-	0	-	0	-	-	-	-	-	-	-	-	-	-	
CV (%)	0	-	0	-	0	-	0	-	-	-	-	-	-	-	-	-	-	
Senna spectabilis	-	-	-	-	14.7	-	-	-	-	-	-	-	-	54.3	38.7	-	-	Anyanwu and Etela, 2013

Average	-	-	-	-	14.7	-	-	-	-	-	-	-	-	54.3	38.7	-	-	Anyanwu and Etela, 2013 (n=1)
Minimum	-	-	-	-	14.7	-	-	-	-	-	-	-	-	54.3	38.7	-	-	
Maximum	-	-	-	-	14.7	-	-	-	-	-	-	-	-	54.3	38.7	-	-	
Standard deviation	-	-	-	-	0	-	-	-	-	-	-	-	-	0	0	-	-	
CV (%)	-	-	-	-	0	-	-	-	-	-	-	-	-	0	0	-	-	
Spermacoce radiata	94.14	-	88.11	-	5.75	-	11.89	-	-	-	-	-	-	-	-	-	-	Sanou et al., 2016
Average	94.14	-	88.11	-	5.75	-	11.89	-	-	-	-	-	-	-	-	-	-	Sanou et al., 2016 (n=1)
Minimum	94.14	-	88.11	-	5.75	-	11.89	-	-	-	-	-	-	-	-	-	-	
Maximum	94.14	-	88.11	-	5.75	-	11.89	-	-	-	-	-	-	-	-	-	-	
Standard deviation	0	-	0	-	0	-	0	-	-	-	-	-	-	-	-	-	-	
CV (%)	0	-	0	-	0	-	0	-	-	-	-	-	-	-	-	-	-	
Spermacoce stachydea	92.96	-	86.14	-	5.63	-	13.86	-	-	-	-	-	-	-	-	-	-	Sanou et al., 2016
Average	92.96	-	86.14	-	5.63	-	13.86	-	-	-	-	-	-	-	-	-	-	Sanou et al., 2016 (n=1)
Minimum	92.96	-	86.14	-	5.63	-	13.86	-	-	-	-	-	-	-	-	-	-	
Maximum	92.96	-	86.14	-	5.63	-	13.86	-	-	-	-	-	-	-	-	-	-	
Standard deviation	0	-	0	-	0	-	0	-	-	-	-	-	-	-	-	-	-	
CV (%)	0	-	0	-	0	-	0	-	-	-	-	-	-	-	-	-	-	
Spondia mombin (from the Guineo-Congolian zone of Benin)	-	-	89.56	-	15.2	-	10.44	-	-	-	-	-	-	53.36	38.11	-	-	Koura et al., 2021
Average	-	-	89.56	-	15.2	-	10.44	-	-	-	-	-	-	53.36	38.11	-	-	Koura et al., 2021 (n=1)
Minimum	-	-	89.56	-	15.2	-	10.44	-	-	-	-	-	-	53.36	38.11	-	-	
Maximum	-	-	89.56	-	15.2	-	10.44	-	-	-	-	-	-	53.36	38.11	-	-	
Standard deviation	-	-	0	-	0	-	0	-	-	-	-	-	-	0	0	-	-	
CV (%)	-	-	0	-	0	-	0	-	-	-	-	-	-	0	0	-	-	
Spondia mombin (from the Sudano-Guinean zone of Benin)	-	-	94.82	-	11.69	-	5.18	-	-	-	-	-	-	43.2	19.75	-	-	Koura et al., 2021
Average	-	-	94.82	-	11.69	-	5.18	-	-	-	-	-	-	43.2	19.75	-	-	Koura et al., 2021 (n=1)
Minimum	-	-	94.82	-	11.69	-	5.18	-	-	-	-	-	-	43.2	19.75	-	-	
Maximum	-	-	94.82	-	11.69	-	5.18	-	-	-	-	-	-	43.2	19.75	-	-	
Standard deviation	-	-	0	-	0	-	0	-	-	-	-	-	-	0	0	-	-	
CV (%)	-	-	0	-	0	-	0	-	-	-	-	-	-	0	0	-	-	
Spondias mombin	-	4.2	89.56	30.65	15.2	-	10.44	-	-	-	-	-	-	53.36	38.11	-	-	Koura et al., 2021 (GCZ)
Spondias mombin	-	4.91	94.82	40.76	11.69	-	5.18	-	-	-	-	-	-	43.2	19.75	-	-	Koura et al., 2021 (SGZ)
Average	-	4.55	92.19	35.7	13.45	-	7.81	-	-	-	-	-	-	48.28	28.93	-	-	Koura et al., 2021 (GCZ); Koura et al., 2021 (SGZ) (n=2)
Minimum	-	4.2	89.56	30.65	11.69	-	5.18	-	-	-	-	-	-	43.2	19.75	-	-	
Maximum	-	4.91	94.82	40.76	15.2	-	10.44	-	-	-	-	-	-	53.36	38.11	-	-	
Standard deviation	-	0.5	3.72	7.15	2.48	-	3.72	-	-	-	-	-	-	7.18	12.98	-	-	
CV (%)	-	11.02	4.03	20.02	18.46	-	47.62	-	-	-	-	-	-	14.88	44.88	-	-	
Tephrosia bracteolata	29.12	3.32	92.94	-	14.88	18.43	7.06	-	-	-	-	-	-	54.1	32.87	-	-	Ogunbosoye and Odedire, 2022
Tephrosia bracteolata	94.1911	2.4975	-	-	-	6.0727	10.2004	-	-	-	-	-	-	-	-	-	-	Mawussi et al., 2022

Average	61.66	2.91	92.94	-	14.88	12.25	8.63	-	-	-	-	-	-	54.1	32.87	-	-	Ogunbosoye and Odedire, 2022; Mawussi et al., 2022 (n=2)
Minimum	29.12	2.5	92.94	-	14.88	6.07	7.06	-	-	-	-	-	-	54.1	32.87	-	-	
Maximum																		
Standard deviation	46.01	0.58	0	-	0	8.74	2.22	-	-	-	-	-	-	0	0	-	-	
CV (%)	74.63	19.99	0	-	0	71.32	25.73	-	-	-	-	-	-	0	0	-	-	
Terminalia catappa	89.4	4.45	92.4	-	8.73	18.3	7.63	-	-	-	-	-	-	45.3	35.5	-	-	Sasu et al., 2023
Average	89.4	4.45	92.4	-	8.73	18.3	7.63	-	-	-	-	-	-	45.3	35.5	-	-	Sasu et al., 2023 (n=1)
Minimum	89.4	4.45	92.4	-	8.73	18.3	7.63	-	-	-	-	-	-	45.3	35.5	-	-	
Maximum	89.4	4.45	92.4	-	8.73	18.3	7.63	-	-	-	-	-	-	45.3	35.5	-	-	
Standard deviation	0	0	0	-	0	0	0	-	-	-	-	-	-	0	0	-	-	
CV (%)	0	0	0	-	0	0	0	-	-	-	-	-	-	0	0	-	-	
Terminalia superba	-	-	-	-	18.1	-	-	-	-	-	-	-	-	58.8	30.3	-	-	Anyanwu and Etela, 2013
Average	-	-	-	-	18.1	-	-	-	-	-	-	-	-	58.8	30.3	-	-	Anyanwu and Etela, 2013 (n=1)
Minimum	-	-	-	-	18.1	-	-	-	-	-	-	-	-	58.8	30.3	-	-	
Maximum	-	-	-	-	18.1	-	-	-	-	-	-	-	-	58.8	30.3	-	-	
Standard deviation	-	-	-	-	0	-	-	-	-	-	-	-	-	0	0	-	-	
CV (%)	-	-	-	-	0	-	-	-	-	-	-	-	-	0	0	-	-	
Tetrapluera tetraptera	-	-	-	-	13.9	-	-	-	-	-	-	-	-	69.5	55.6	-	-	Anyanwu and Etela, 2013
Average	-	-	-	-	13.9	-	-	-	-	-	-	-	-	69.5	55.6	-	-	Anyanwu and Etela, 2013 (n=1)
Minimum	-	-	-	-	13.9	-	-	-	-	-	-	-	-	69.5	55.6	-	-	
Maximum	-	-	-	-	13.9	-	-	-	-	-	-	-	-	69.5	55.6	-	-	
Standard deviation	-	-	-	-	0	-	-	-	-	-	-	-	-	0	0	-	-	
CV (%)	-	-	-	-	0	-	-	-	-	-	-	-	-	0	0	-	-	
Treculia africana	-	-	-	-	13.9	-	-	-	-	-	-	-	-	62.2	37.5	-	-	Anyanwu and Etela, 2013
Average	-	-	-	-	13.9	-	-	-	-	-	-	-	-	62.2	37.5	-	-	Anyanwu and Etela, 2013 (n=1)
Minimum	-	-	-	-	13.9	-	-	-	-	-	-	-	-	62.2	37.5	-	-	
Maximum	-	-	-	-	13.9	-	-	-	-	-	-	-	-	62.2	37.5	-	-	
Standard deviation	-	-	-	-	0	-	-	-	-	-	-	-	-	0	0	-	-	
CV (%)	-	-	-	-	0	-	-	-	-	-	-	-	-	0	0	-	-	
Vigna unguiculata	94.55	-	92.58	-	12.47	-	7.42	-	-	-	-	0.66	-	-	-	-	-	Sanou et al., 2016
Average	94.55	-	92.58	-	12.47	-	7.42	-	-	-	-	0.66	-	-	-	-	-	Sanou et al., 2016 (n=1)
Minimum	94.55	-	92.58	-	12.47	-	7.42	-	-	-	-	0.66	-	-	-	-	-	
Maximum	94.55	-	92.58	-	12.47	-	7.42	-	-	-	-	0.66	-	-	-	-	-	
Standard deviation	0	-	0	-	0	-	0	-	-	-	-	0	-	-	-	-	-	
CV (%)	0	-	0	-	0	-	0	-	-	-	-	0	-	-	-	-	-	
Xylia xalocarpa	-	-	-	-	13.3	-	-	-	-	-	-	-	-	65	51.9	-	-	Anyanwu and Etela, 2013
Average	-	-	-	-	13.3	-	-	-	-	-	-	-	-	65	51.9	-	-	Anyanwu and Etela, 2013 (n=1)
Minimum	-	-	-	-	13.3	-	-	-	-	-	-	-	-	65	51.9	-	-	
Maximum	-	-	-	-	13.3	-	-	-	-	-	-	-	-	65	51.9	-	-	
Standard deviation	-	-	-	-	0	-	-	-	-	-	-	-	-	0	0	-	-	
CV (%)	-	-	-	-	0	-	-	-	-	-	-	-	-	0	0	-	-	

Zanthoxylum zanthoxyloides	-	3.22	90.77	48.29	11.61	-	9.23	-	-	-	-	-	-	43.19	24.92	-	-	Koura et al., 2021
Average	-	3.22	90.77	48.29	11.61	-	9.23	-	-	-	-	-	-	43.19	24.92	-	-	Koura et al., 2021 (n=1)
Minimum	-	3.22	90.77	48.29	11.61	-	9.23	-	-	-	-	-	-	43.19	24.92	-	-	
Maximum	-	3.22	90.77	48.29	11.61	-	9.23	-	-	-	-	-	-	43.19	24.92	-	-	
Standard deviation	-	0	0	0	0	-	0	-	-	-	-	-	-	0	0	-	-	
CV (%)	-	0	0	0	0	-	0	-	-	-	-	-	-	0	0	-	-	
Zea mays	94.51	-	93.6	-	1.15	-	6.4	-	-	-	-	0.71	-	-	-	-	-	Sanou et al., 2016
Average	94.51	-	93.6	-	1.15	-	6.4	-	-	-	-	0.71	-	-	-	-	-	Sanou et al., 2016 (n=1)
Minimum	94.51	-	93.6	-	1.15	-	6.4	-	-	-	-	0.71	-	-	-	-	-	
Maximum	94.51	-	93.6	-	1.15	-	6.4	-	-	-	-	0.71	-	-	-	-	-	
Standard deviation	0	-	0	-	0	-	0	-	-	-	-	0	-	-	-	-	-	
CV (%)	0	-	0	-	0	-	0	-	-	-	-	0	-	-	-	-	-	
Ziziphus abyssinica	97	2	83	-	14.37	-	14	-	-	-	-	-	-	39.6	16.2	1.1	2	Njidda, 2011
Average	97	2	83	-	14.37	-	14	-	-	-	-	-	-	39.6	16.2	1.1	2	Njidda, 2011 (n=1)
Minimum	97	2	83	-	14.37	-	14	-	-	-	-	-	-	39.6	16.2	1.1	2	
Maximum	97	2	83	-	14.37	-	14	-	-	-	-	-	-	39.6	16.2	1.1	2	
Standard deviation	0	0	0	-	0	-	0	-	-	-	-	-	-	0	0	0	0	
CV (%)	0	0	0	-	0	-	0	-	-	-	-	-	-	0	0	0	0	
Zornia latifolia	89.56	5.28	90.59	58.45	14.9	-	9.41	-	-	11.19	-	-	-	43.97	38.31	-	-	Koura et al., 2022
Average	89.56	5.28	90.59	58.45	14.9	-	9.41	-	-	11.19	-	-	-	43.97	38.31	-	-	Koura et al., 2022 (n=1)
Minimum	89.56	5.28	90.59	58.45	14.9	-	9.41	-	-	11.19	-	-	-	43.97	38.31	-	-	
Maximum	89.56	5.28	90.59	58.45	14.9	-	9.41	-	-	11.19	-	-	-	43.97	38.31	-	-	
Standard deviation	0	0	0	0	0	-	0	-	-	0	-	-	-	0	0	-	-	
CV (%)	0	0	0	0	0	-	0	-	-	0	-	-	-	0	0	-	-	
Samanea saman	73.9	-	92.23	-	24.05	-	7.77	-	-	-	-	-	-	40.62	31.35	-	-	Idan et al., 2023
Average	73.9	-	92.23	-	24.05	-	7.77	-	-	-	-	-	-	40.62	31.35	-	-	Idan et al., 2023 (n=1)
Minimum	73.9	-	92.23	-	24.05	-	7.77	-	-	-	-	-	-	40.62	31.35	-	-	
Maximum	73.9	-	92.23	-	24.05	-	7.77	-	-	-	-	-	-	40.62	31.35	-	-	
Standard deviation	0	-	0	-	0	-	0	-	-	-	-	-	-	0	0	-	-	
CV (%)	0	-	0	-	0	-	0	-	-	-	-	-	-	0	0	-	-	
Sweet potato foliage (TIS-87/0087)	14	-	89.1	-	10.06	-	10.9	-	-	-	-	-	-	43.9	-	-	-	Etela et al., 2008
Average	14	-	89.1	-	10.06	-	10.9	-	-	-	-	-	-	43.9	-	-	-	Etela et al., 2008 (n=1)
Minimum	14	-	89.1	-	10.06	-	10.9	-	-	-	-	-	-	43.9	-	-	-	
Maximum	14	-	89.1	-	10.06	-	10.9	-	-	-	-	-	-	43.9	-	-	-	
Standard deviation	0	-	0	-	0	-	0	-	-	-	-	-	-	0	-	-	-	
CV (%)	0	-	0	-	0	-	0	-	-	-	-	-	-	0	-	-	-	
Sweet potato foliage (TIS-8164)	13	-	91.1	-	9.13	-	8.9	-	-	-	-	-	-	47.1	-	-	-	Etela et al., 2008
Average	13	-	91.1	-	9.13	-	8.9	-	-	-	-	-	-	47.1	-	-	-	Etela et al., 2008 (n=1)
Minimum	13	-	91.1	-	9.13	-	8.9	-	-	-	-	-	-	47.1	-	-	-	
Maximum	13	-	91.1	-	9.13	-	8.9	-	-	-	-	-	-	47.1	-	-	-	
Standard deviation	0	-	0	-	0	-	0	-	-	-	-	-	-	0	-	-	-	

CV (%)	0	-	0	-	0	-	0	-	-	-	-	-	-	0	-	-	-	
Sweet potato foliage (TIS-2532.OP.1.13)	14	-	89.8	-	7.5	-	10.3	-	-	-	-	-	-	50.2	-	-	-	Etela et al., 2008
Average	14	-	89.8	-	7.5	-	10.3	-	-	-	-	-	-	50.2	-	-	-	Etela et al., 2008 (n=1)
Minimum	14	-	89.8	-	7.5	-	10.3	-	-	-	-	-	-	50.2	-	-	-	
Maximum	14	-	89.8	-	7.5	-	10.3	-	-	-	-	-	-	50.2	-	-	-	
Standard deviation	0	-	0	-	0	-	0	-	-	-	-	-	-	0	-	-	-	
CV (%)	0	-	0	-	0	-	0	-	-	-	-	-	-	0	-	-	-	
Faidherbia albida pods	92.57	-	88.55	-	6.1	28.04	4.02	-	-	-	2.14	-	-	40.58	29.92	-	-	Kiema et al., 2019
Average	92.57	-	88.55	-	6.1	28.04	4.02	-	-	-	2.14	-	-	40.58	29.92	-	-	Kiema et al., 2019 (n=1)
Minimum	92.57	-	88.55	-	6.1	28.04	4.02	-	-	-	2.14	-	-	40.58	29.92	-	-	
Maximum	92.57	-	88.55	-	6.1	28.04	4.02	-	-	-	2.14	-	-	40.58	29.92	-	-	
Standard deviation	0	-	0	-	0	0	0	-	-	-	0	-	-	0	0	-	-	
CV (%)	0	-	0	-	0	0	0	-	-	-	0	-	-	0	0	-	-	
Cowpea hay	90.7	-	92.1	61.2	14.37	-	7.9	-	-	8.94	-	-	-	46.9	33.2	-	-	Umutoni et al., 2021
Average	90.7	-	92.1	61.2	14.37	-	7.9	-	-	8.94	-	-	-	46.9	33.2	-	-	Umutoni et al., 2021 (n=1)
Minimum	90.7	-	92.1	61.2	14.37	-	7.9	-	-	8.94	-	-	-	46.9	33.2	-	-	
Maximum	90.7	-	92.1	61.2	14.37	-	7.9	-	-	8.94	-	-	-	46.9	33.2	-	-	
Standard deviation	0	-	0	0	0	-	0	-	-	0	-	-	-	0	0	-	-	
CV (%)	0	-	0	0	0	-	0	-	-	0	-	-	-	0	0	-	-	
Bamboo leaves [air-dried]	89.31	7.51	88.84	-	20.99	25.1	11.16	-	-	-	-	-	-	74.49	49.1	1.09	0.03	Akinmoladun, 2022
Average	89.31	7.51	88.84	-	20.99	25.1	11.16	-	-	-	-	-	-	74.49	49.1	1.09	0.03	Akinmoladun, 2022 (n=1)
Minimum	89.31	7.51	88.84	-	20.99	25.1	11.16	-	-	-	-	-	-	74.49	49.1	1.09	0.03	
Maximum	89.31	7.51	88.84	-	20.99	25.1	11.16	-	-	-	-	-	-	74.49	49.1	1.09	0.03	
Standard deviation	0	0	0	-	0	0	0	-	-	-	-	-	-	0	0	0	0	
CV (%)	0	0	0	-	0	0	0	-	-	-	-	-	-	0	0	0	0	
Bamboo leaves [fermented, 5 d]	89.31	7.43	88.19	-	16.63	24.86	11.81	-	-	-	-	-	-	69.13	43.1	1.11	0.04	Akinmoladun, 2022
Average	89.31	7.43	88.19	-	16.63	24.86	11.81	-	-	-	-	-	-	69.13	43.1	1.11	0.04	Akinmoladun, 2022 (n=1)
Minimum	89.31	7.43	88.19	-	16.63	24.86	11.81	-	-	-	-	-	-	69.13	43.1	1.11	0.04	
Maximum	89.31	7.43	88.19	-	16.63	24.86	11.81	-	-	-	-	-	-	69.13	43.1	1.11	0.04	
Standard deviation	0	0	0	-	0	0	0	-	-	-	-	-	-	0	0	0	0	
CV (%)	0	0	0	-	0	0	0	-	-	-	-	-	-	0	0	0	0	
Bamboo leaves [soaked in water, 24 h]	89.29	7.05	90.02	-	24.67	23.61	9.98	-	-	-	-	-	-	71.77	51.68	1.27	0.04	Akinmoladun, 2022
Average	89.29	7.05	90.02	-	24.67	23.61	9.98	-	-	-	-	-	-	71.77	51.68	1.27	0.04	Akinmoladun, 2022 (n=1)
Minimum	89.29	7.05	90.02	-	24.67	23.61	9.98	-	-	-	-	-	-	71.77	51.68	1.27	0.04	
Maximum	89.29	7.05	90.02	-	24.67	23.61	9.98	-	-	-	-	-	-	71.77	51.68	1.27	0.04	
Standard deviation	0	0	0	-	0	0	0	-	-	-	-	-	-	0	0	0	0	
CV (%)	0	0	0	-	0	0	0	-	-	-	-	-	-	0	0	0	0	
Bamboo leaves [soaked in warm water, 20 min]	89.81	6.95	89.65	-	24.24	25.77	10.35	-	-	-	-	-	-	72.74	46.65	1.18	0.05	Akinmoladun, 2022

Average	89.81	6.95	89.65	-	24.24	25.77	10.35	-	-	-	-	-	-	72.74	46.65	1.18	0.05	Akinmoladun, 2022 (n=1)
Minimum	89.81	6.95	89.65	-	24.24	25.77	10.35	-	-	-	-	-	-	72.74	46.65	1.18	0.05	
Maximum	89.81	6.95	89.65	-	24.24	25.77	10.35	-	-	-	-	-	-	72.74	46.65	1.18	0.05	
Standard deviation	0	0	0	-	0	0	0	-	-	-	-	-	-	0	0	0	0	
CV (%)	0	0	0	-	0	0	0	-	-	-	-	-	-	0	0	0	0	
Bamboo leaves [fresh]	61.52	4.62	93.1	-	12.27	33.21	6.9	-	-	-	-	-	-	40.59	26.83	1.29	0.02	Akinmoladun, 2022
Average	61.52	4.62	93.1	-	12.27	33.21	6.9	-	-	-	-	-	-	40.59	26.83	1.29	0.02	Akinmoladun, 2022 (n=1)
Minimum	61.52	4.62	93.1	-	12.27	33.21	6.9	-	-	-	-	-	-	40.59	26.83	1.29	0.02	
Maximum	61.52	4.62	93.1	-	12.27	33.21	6.9	-	-	-	-	-	-	40.59	26.83	1.29	0.02	
Standard deviation	0	0	0	-	0	0	0	-	-	-	-	-	-	0	0	0	0	
CV (%)	0	0	0	-	0	0	0	-	-	-	-	-	-	0	0	0	0	
Oil palm leaves	89.8	3.9	87.47	-	9.92	22.89	12.53	-	-	11.32	-	-	-	-	-	-	-	Essen et al., 2025
Average	89.8	3.9	87.47	-	9.92	22.89	12.53	-	-	11.32	-	-	-	-	-	-	-	Essen et al., 2025 (n=1)
Minimum	89.8	3.9	87.47	-	9.92	22.89	12.53	-	-	11.32	-	-	-	-	-	-	-	
Maximum	89.8	3.9	87.47	-	9.92	22.89	12.53	-	-	11.32	-	-	-	-	-	-	-	
Standard deviation	0	0	0	-	0	0	0	-	-	0	-	-	-	-	-	-	-	
CV (%)	0	0	0	-	0	0	0	-	-	0	-	-	-	-	-	-	-	
Andropogon macrophyllus	90.19	4.4	91.47	-	9.74	-	8.53	-	-	14.75713536	-	-	-	-	-	-	-	Kouadio et al., 2024
Average	90.19	4.4	91.47	-	9.74	-	8.53	-	-	14.76	-	-	-	-	-	-	-	Kouadio et al., 2024 (n=1)
Minimum	90.19	4.4	91.47	-	9.74	-	8.53	-	-	14.76	-	-	-	-	-	-	-	
Maximum	90.19	4.4	91.47	-	9.74	-	8.53	-	-	14.76	-	-	-	-	-	-	-	
Standard deviation	0	0	0	-	0	-	0	-	-	0	-	-	-	-	-	-	-	
CV (%)	0	0	0	-	0	-	0	-	-	0	-	-	-	-	-	-	-	
Rottboellia cochinchinensis	86.61	5.95	91.68	-	14.56	-	8.32	-	-	14.40145352	-	-	-	-	-	-	-	Kouadio et al., 2024
Average	86.61	5.95	91.68	-	14.56	-	8.32	-	-	14.4	-	-	-	-	-	-	-	Kouadio et al., 2024 (n=1)
Minimum	86.61	5.95	91.68	-	14.56	-	8.32	-	-	14.4	-	-	-	-	-	-	-	
Maximum	86.61	5.95	91.68	-	14.56	-	8.32	-	-	14.4	-	-	-	-	-	-	-	
Standard deviation	0	0	0	-	0	-	0	-	-	0	-	-	-	-	-	-	-	
CV (%)	0	0	0	-	0	-	0	-	-	0	-	-	-	-	-	-	-	
Panicum laxum	77.02	9.8	90.41	-	8.07	-	9.59	-	-	13.28185696	-	-	-	-	-	-	-	Kouadio et al., 2024
Average	77.02	9.8	90.41	-	8.07	-	9.59	-	-	13.28	-	-	-	-	-	-	-	Kouadio et al., 2024 (n=1)
Minimum	77.02	9.8	90.41	-	8.07	-	9.59	-	-	13.28	-	-	-	-	-	-	-	
Maximum	77.02	9.8	90.41	-	8.07	-	9.59	-	-	13.28	-	-	-	-	-	-	-	
Standard deviation	0	0	0	-	0	-	0	-	-	0	-	-	-	-	-	-	-	
CV (%)	0	0	0	-	0	-	0	-	-	0	-	-	-	-	-	-	-	
Centrosema pubescens	87.53	6.4	90.53	-	18.29	-	9.47	-	-	14.40337816	-	-	-	-	-	-	-	Kouadio et al., 2024
Average	87.53	6.4	90.53	-	18.29	-	9.47	-	-	14.4	-	-	-	-	-	-	-	Kouadio et al., 2024 (n=1)
Minimum	87.53	6.4	90.53	-	18.29	-	9.47	-	-	14.4	-	-	-	-	-	-	-	
Maximum	87.53	6.4	90.53	-	18.29	-	9.47	-	-	14.4	-	-	-	-	-	-	-	
Standard deviation	0	0	0	-	0	-	0	-	-	0	-	-	-	-	-	-	-	

CV (%)	0	0	0	-	0	-	0	-	-	0	-	-	-	-	-	-	-	
Eriosema glomeratum	83.93	9.39	91.85	-	16.88	-	8.15	-	-	14.55734936	-	-	-	-	-	-	-	Kouadio et al., 2024
Average	83.93	9.39	91.85	-	16.88	-	8.15	-	-	14.56	-	-	-	-	-	-	-	Kouadio et al., 2024 (n=1)
Minimum	83.93	9.39	91.85	-	16.88	-	8.15	-	-	14.56	-	-	-	-	-	-	-	
Maximum	83.93	9.39	91.85	-	16.88	-	8.15	-	-	14.56	-	-	-	-	-	-	-	
Standard deviation	0	0	0	-	0	-	0	-	-	0	-	-	-	-	-	-	-	
CV (%)	0	0	0	-	0	-	0	-	-	0	-	-	-	-	-	-	-	
Psophocarpus palustris	84.08	3.79	90.81	-	5.52	-	9.19	-	-	13.52402688	-	-	-	-	-	-	-	Kouadio et al., 2024
Average	84.08	3.79	90.81	-	5.52	-	9.19	-	-	13.52	-	-	-	-	-	-	-	Kouadio et al., 2024 (n=1)
Minimum	84.08	3.79	90.81	-	5.52	-	9.19	-	-	13.52	-	-	-	-	-	-	-	
Maximum	84.08	3.79	90.81	-	5.52	-	9.19	-	-	13.52	-	-	-	-	-	-	-	
Standard deviation	0	0	0	-	0	-	0	-	-	0	-	-	-	-	-	-	-	
CV (%)	0	0	0	-	0	-	0	-	-	0	-	-	-	-	-	-	-	
Desmodium velutinum	86.69	10.4	91.83	-	19.01	-	8.17	-	-	15.18699952	-	-	-	-	-	-	-	Kouadio et al., 2024
Average	86.69	10.4	91.83	-	19.01	-	8.17	-	-	15.19	-	-	-	-	-	-	-	Kouadio et al., 2024 (n=1)
Minimum	86.69	10.4	91.83	-	19.01	-	8.17	-	-	15.19	-	-	-	-	-	-	-	
Maximum	86.69	10.4	91.83	-	19.01	-	8.17	-	-	15.19	-	-	-	-	-	-	-	
Standard deviation	0	0	0	-	0	-	0	-	-	0	-	-	-	-	-	-	-	
CV (%)	0	0	0	-	0	-	0	-	-	0	-	-	-	-	-	-	-	
Antiaris toxicaria	87.26	11.4	91.01	-	14.38	-	8.99	-	-	15.36896168	-	-	-	-	-	-	-	Kouadio et al., 2024
Average	87.26	11.4	91.01	-	14.38	-	8.99	-	-	15.37	-	-	-	-	-	-	-	Kouadio et al., 2024 (n=1)
Minimum	87.26	11.4	91.01	-	14.38	-	8.99	-	-	15.37	-	-	-	-	-	-	-	
Maximum	87.26	11.4	91.01	-	14.38	-	8.99	-	-	15.37	-	-	-	-	-	-	-	
Standard deviation	0	0	0	-	0	-	0	-	-	0	-	-	-	-	-	-	-	
CV (%)	0	0	0	-	0	-	0	-	-	0	-	-	-	-	-	-	-	
Ficus exasperata	86.92	4.2	90.79	-	12.68	-	9.21	-	-	14.00878512	-	-	-	-	-	-	-	Kouadio et al., 2024
Average	86.92	4.2	90.79	-	12.68	-	9.21	-	-	14.01	-	-	-	-	-	-	-	Kouadio et al., 2024 (n=1)
Minimum	86.92	4.2	90.79	-	12.68	-	9.21	-	-	14.01	-	-	-	-	-	-	-	
Maximum	86.92	4.2	90.79	-	12.68	-	9.21	-	-	14.01	-	-	-	-	-	-	-	
Standard deviation	0	0	0	-	0	-	0	-	-	0	-	-	-	-	-	-	-	
CV (%)	0	0	0	-	0	-	0	-	-	0	-	-	-	-	-	-	-	
Margaritaria discoidea	75	9.6	90.59	-	23.46	-	9.41	-	-	12.76868936	-	-	-	-	-	-	-	Kouadio et al., 2024
Average	75	9.6	90.59	-	23.46	-	9.41	-	-	12.77	-	-	-	-	-	-	-	Kouadio et al., 2024 (n=1)
Minimum	75	9.6	90.59	-	23.46	-	9.41	-	-	12.77	-	-	-	-	-	-	-	
Maximum	75	9.6	90.59	-	23.46	-	9.41	-	-	12.77	-	-	-	-	-	-	-	
Standard deviation	0	0	0	-	0	-	0	-	-	0	-	-	-	-	-	-	-	
CV (%)	0	0	0	-	0	-	0	-	-	0	-	-	-	-	-	-	-	

Albizia zygia	89.28	5.06	90.73	-	18.69	-	9.27	-	-	14.49534<br>248	-	-	-	-	-	-	-	Kouadio et al., 2024
Average	89.28	5.06	90.73	-	18.69	-	9.27	-	-	14.5	-	-	-	-	-	-	-	Kouadio et al., 2024 (n=1)
Minimum	89.28	5.06	90.73	-	18.69	-	9.27	-	-	14.5	-	-	-	-	-	-	-	
Maximum	89.28	5.06	90.73	-	18.69	-	9.27	-	-	14.5	-	-	-	-	-	-	-	
Standard deviation	0	0	0	-	0	-	0	-	-	0	-	-	-	-	-	-	-	
CV (%)	0	0	0	-	0	-	0	-	-	0	-	-	-	-	-	-	-	
Acacia sieberiana	45.7	-	96.2	46.4	18.2	36.5	-	-	-	-	134	0.61	0.51	67.1	65.7	-	-	Sidiimorou et al., 2016
Average	45.7	-	96.2	46.4	18.2	36.5	-	-	-	-	134	0.61	0.51	67.1	65.7	-	-	Sidiimorou et al., 2016 (n=1)
Minimum	45.7	-	96.2	46.4	18.2	36.5	-	-	-	-	134	0.61	0.51	67.1	65.7	-	-	
Maximum	45.7	-	96.2	46.4	18.2	36.5	-	-	-	-	134	0.61	0.51	67.1	65.7	-	-	
Standard deviation	0	-	0	0	0	0	-	-	-	-	0	0	0	0	0	-	-	
CV (%)	0	-	0	0	0	0	-	-	-	-	0	0	0	0	0	-	-	
Bombax costatum	60	-	95.1	44.8	12.2	19.6	-	-	-	-	78	0.6	0.5	64.5	49.9	-	-	Sidiimorou et al., 2016
Average	60	-	95.1	44.8	12.2	19.6	-	-	-	-	78	0.6	0.5	64.5	49.9	-	-	Sidiimorou et al., 2016 (n=1)
Minimum	60	-	95.1	44.8	12.2	19.6	-	-	-	-	78	0.6	0.5	64.5	49.9	-	-	
Maximum	60	-	95.1	44.8	12.2	19.6	-	-	-	-	78	0.6	0.5	64.5	49.9	-	-	
Standard deviation	0	-	0	0	0	0	-	-	-	-	0	0	0	0	0	-	-	
CV (%)	0	-	0	0	0	0	-	-	-	-	0	0	0	0	0	-	-	
Dichrostachys cineria	57.7	-	95.6	43.9	13.7	19.6	-	-	-	-	92	0.58	0.48	57.9	54.8	-	-	Sidiimorou et al., 2016
Average	57.7	-	95.6	43.9	13.7	19.6	-	-	-	-	92	0.58	0.48	57.9	54.8	-	-	Sidiimorou et al., 2016 (n=1)
Minimum	57.7	-	95.6	43.9	13.7	19.6	-	-	-	-	92	0.58	0.48	57.9	54.8	-	-	
Maximum	57.7	-	95.6	43.9	13.7	19.6	-	-	-	-	92	0.58	0.48	57.9	54.8	-	-	
Standard deviation	0	-	0	0	0	0	-	-	-	-	0	0	0	0	0	-	-	
CV (%)	0	-	0	0	0	0	-	-	-	-	0	0	0	0	0	-	-	
Fluggea virosa	49.2	-	96.4	50	15.3	11	-	-	-	-	107	0.66	0.56	85	66.6	-	-	Sidiimorou et al., 2016
Average	49.2	-	96.4	50	15.3	11	-	-	-	-	107	0.66	0.56	85	66.6	-	-	Sidiimorou et al., 2016 (n=1)
Minimum	49.2	-	96.4	50	15.3	11	-	-	-	-	107	0.66	0.56	85	66.6	-	-	
Maximum	49.2	-	96.4	50	15.3	11	-	-	-	-	107	0.66	0.56	85	66.6	-	-	
Standard deviation	0	-	0	0	0	0	-	-	-	-	0	0	0	0	0	-	-	
CV (%)	0	-	0	0	0	0	-	-	-	-	0	0	0	0	0	-	-	
Gardenia erubescens	48.6	-	95.3	48.6	12.2	23.8	-	-	-	-	78	0.66	0.56	68.6	58.2	-	-	Sidiimorou et al., 2016
Average	48.6	-	95.3	48.6	12.2	23.8	-	-	-	-	78	0.66	0.56	68.6	58.2	-	-	Sidiimorou et al., 2016 (n=1)
Minimum	48.6	-	95.3	48.6	12.2	23.8	-	-	-	-	78	0.66	0.56	68.6	58.2	-	-	
Maximum	48.6	-	95.3	48.6	12.2	23.8	-	-	-	-	78	0.66	0.56	68.6	58.2	-	-	
Standard deviation	0	-	0	0	0	0	-	-	-	-	0	0	0	0	0	-	-	
CV (%)	0	-	0	0	0	0	-	-	-	-	0	0	0	0	0	-	-	
Hymenocardia acida	60.7	-	94.1	45.2	10.6	17.2	-	-	-	-	63	0.62	0.52	42.6	40.6	-	-	Sidiimorou et al., 2016
Average	60.7	-	94.1	45.2	10.6	17.2	-	-	-	-	63	0.62	0.52	42.6	40.6	-	-	Sidiimorou et al., 2016 (n=1)
Minimum	60.7	-	94.1	45.2	10.6	17.2	-	-	-	-	63	0.62	0.52	42.6	40.6	-	-	
Maximum	60.7	-	94.1	45.2	10.6	17.2	-	-	-	-	63	0.62	0.52	42.6	40.6	-	-	
Standard deviation	0	-	0	0	0	0	-	-	-	-	0	0	0	0	0	-	-	
CV (%)	0	-	0	0	0	0	-	-	-	-	0	0	0	0	0	-	-	

Khaya senegalensis	42.3	-	94.3	38.2	10.3	26.2	-	-	-	-	60	0.52	0.41	50.5	49.1	-	-	Sidiimorou et al., 2016
Average	42.3	-	94.3	38.2	10.3	26.2	-	-	-	-	60	0.52	0.41	50.5	49.1	-	-	Sidiimorou et al., 2016 (n=1)
Minimum	42.3	-	94.3	38.2	10.3	26.2	-	-	-	-	60	0.52	0.41	50.5	49.1	-	-	
Maximum	42.3	-	94.3	38.2	10.3	26.2	-	-	-	-	60	0.52	0.41	50.5	49.1	-	-	
Standard deviation	0	-	0	0	0	0	-	-	-	-	0	0	0	0	0	-	-	
CV (%)	0	-	0	0	0	0	-	-	-	-	0	0	0	0	0	-	-	
Lonchocarpus laxifolus	45.7	-	92.8	46.9	18.6	22.5	-	-	-	-	138	0.66	0.56	58.6	54.3	-	-	Sidiimorou et al., 2016
Average	45.7	-	92.8	46.9	18.6	22.5	-	-	-	-	138	0.66	0.56	58.6	54.3	-	-	Sidiimorou et al., 2016 (n=1)
Minimum	45.7	-	92.8	46.9	18.6	22.5	-	-	-	-	138	0.66	0.56	58.6	54.3	-	-	
Maximum	45.7	-	92.8	46.9	18.6	22.5	-	-	-	-	138	0.66	0.56	58.6	54.3	-	-	
Standard deviation	0	-	0	0	0	0	-	-	-	-	0	0	0	0	0	-	-	
CV (%)	0	-	0	0	0	0	-	-	-	-	0	0	0	0	0	-	-	
Maranthes polyandra	47.3	-	95	36	9.8	19.5	-	-	-	-	56	0.48	0.37	83.6	72.8	-	-	Sidiimorou et al., 2016
Average	47.3	-	95	36	9.8	19.5	-	-	-	-	56	0.48	0.37	83.6	72.8	-	-	Sidiimorou et al., 2016 (n=1)
Minimum	47.3	-	95	36	9.8	19.5	-	-	-	-	56	0.48	0.37	83.6	72.8	-	-	
Maximum	47.3	-	95	36	9.8	19.5	-	-	-	-	56	0.48	0.37	83.6	72.8	-	-	
Standard deviation	0	-	0	0	0	0	-	-	-	-	0	0	0	0	0	-	-	
CV (%)	0	-	0	0	0	0	-	-	-	-	0	0	0	0	0	-	-	
Monotes kerstingii	54.3	-	94.7	38	5.8	21.6	-	-	-	-	19	0.51	0.4	65.2	58	-	-	Sidiimorou et al., 2016
Average	54.3	-	94.7	38	5.8	21.6	-	-	-	-	19	0.51	0.4	65.2	58	-	-	Sidiimorou et al., 2016 (n=1)
Minimum	54.3	-	94.7	38	5.8	21.6	-	-	-	-	19	0.51	0.4	65.2	58	-	-	
Maximum	54.3	-	94.7	38	5.8	21.6	-	-	-	-	19	0.51	0.4	65.2	58	-	-	
Standard deviation	0	-	0	0	0	0	-	-	-	-	0	0	0	0	0	-	-	
CV (%)	0	-	0	0	0	0	-	-	-	-	0	0	0	0	0	-	-	
Nauclea latifolia	55.1	-	95.8	39.9	14.9	33.7	-	-	-	-	103	0.53	0.41	63.2	61.9	-	-	Sidiimorou et al., 2016
Average	55.1	-	95.8	39.9	14.9	33.7	-	-	-	-	103	0.53	0.41	63.2	61.9	-	-	Sidiimorou et al., 2016 (n=1)
Minimum	55.1	-	95.8	39.9	14.9	33.7	-	-	-	-	103	0.53	0.41	63.2	61.9	-	-	
Maximum	55.1	-	95.8	39.9	14.9	33.7	-	-	-	-	103	0.53	0.41	63.2	61.9	-	-	
Standard deviation	0	-	0	0	0	0	-	-	-	-	0	0	0	0	0	-	-	
CV (%)	0	-	0	0	0	0	-	-	-	-	0	0	0	0	0	-	-	
Phyllanthus muellerianus	54	-	96.3	54.8	10	21.9	-	-	-	-	58	0.73	0.65	50.2	49	-	-	Sidiimorou et al., 2016
Average	54	-	96.3	54.8	10	21.9	-	-	-	-	58	0.73	0.65	50.2	49	-	-	Sidiimorou et al., 2016 (n=1)
Minimum	54	-	96.3	54.8	10	21.9	-	-	-	-	58	0.73	0.65	50.2	49	-	-	
Maximum	54	-	96.3	54.8	10	21.9	-	-	-	-	58	0.73	0.65	50.2	49	-	-	
Standard deviation	0	-	0	0	0	0	-	-	-	-	0	0	0	0	0	-	-	
CV (%)	0	-	0	0	0	0	-	-	-	-	0	0	0	0	0	-	-	
Prosopis africana	53.1	-	95.4	48.8	17.4	16.5	-	-	-	-	126	0.66	0.56	38.3	30.8	-	-	Sidiimorou et al., 2016
Average	53.1	-	95.4	48.8	17.4	16.5	-	-	-	-	126	0.66	0.56	38.3	30.8	-	-	Sidiimorou et al., 2016 (n=1)
Minimum	53.1	-	95.4	48.8	17.4	16.5	-	-	-	-	126	0.66	0.56	38.3	30.8	-	-	
Maximum	53.1	-	95.4	48.8	17.4	16.5	-	-	-	-	126	0.66	0.56	38.3	30.8	-	-	
Standard deviation	0	-	0	0	0	0	-	-	-	-	0	0	0	0	0	-	-	
CV (%)	0	-	0	0	0	0	-	-	-	-	0	0	0	0	0	-	-	

Sarcocephalus latifolius	65	-	96	39.4	11	22.5	-	-	-	-	67	0.52	0.4	67.7	67	-	-	Sidiimorou et al., 2016
Average	65	-	96	39.4	11	22.5	-	-	-	-	67	0.52	0.4	67.7	67	-	-	Sidiimorou et al., 2016 (n=1)
Minimum	65	-	96	39.4	11	22.5	-	-	-	-	67	0.52	0.4	67.7	67	-	-	
Maximum	65	-	96	39.4	11	22.5	-	-	-	-	67	0.52	0.4	67.7	67	-	-	
Standard deviation	0	-	0	0	0	0	-	-	-	-	0	0	0	0	0	-	-	
CV (%)	0	-	0	0	0	0	-	-	-	-	0	0	0	0	0	-	-	
Securidaca longipediculata	37.3	-	93.3	49.1	10.4	23.1	-	-	-	-	61	0.69	0.59	56.1	39.9	-	-	Sidiimorou et al., 2016
Average	37.3	-	93.3	49.1	10.4	23.1	-	-	-	-	61	0.69	0.59	56.1	39.9	-	-	Sidiimorou et al., 2016 (n=1)
Minimum	37.3	-	93.3	49.1	10.4	23.1	-	-	-	-	61	0.69	0.59	56.1	39.9	-	-	
Maximum	37.3	-	93.3	49.1	10.4	23.1	-	-	-	-	61	0.69	0.59	56.1	39.9	-	-	
Standard deviation	0	-	0	0	0	0	-	-	-	-	0	0	0	0	0	-	-	
CV (%)	0	-	0	0	0	0	-	-	-	-	0	0	0	0	0	-	-	
Stereospermum kunthianum	45.9	-	95.9	35.1	11.6	27.1	-	-	-	-	73	0.46	0.34	58.4	54.1	-	-	Sidiimorou et al., 2016
Average	45.9	-	95.9	35.1	11.6	27.1	-	-	-	-	73	0.46	0.34	58.4	54.1	-	-	Sidiimorou et al., 2016 (n=1)
Minimum	45.9	-	95.9	35.1	11.6	27.1	-	-	-	-	73	0.46	0.34	58.4	54.1	-	-	
Maximum	45.9	-	95.9	35.1	11.6	27.1	-	-	-	-	73	0.46	0.34	58.4	54.1	-	-	
Standard deviation	0	-	0	0	0	0	-	-	-	-	0	0	0	0	0	-	-	
CV (%)	0	-	0	0	0	0	-	-	-	-	0	0	0	0	0	-	-	
Strychnos spinosa	43.1	-	95.4	47.5	11.3	16.2	-	-	-	-	70	0.64	0.54	28.7	20.6	-	-	Sidiimorou et al., 2016
Average	43.1	-	95.4	47.5	11.3	16.2	-	-	-	-	70	0.64	0.54	28.7	20.6	-	-	Sidiimorou et al., 2016 (n=1)
Minimum	43.1	-	95.4	47.5	11.3	16.2	-	-	-	-	70	0.64	0.54	28.7	20.6	-	-	
Maximum	43.1	-	95.4	47.5	11.3	16.2	-	-	-	-	70	0.64	0.54	28.7	20.6	-	-	
Standard deviation	0	-	0	0	0	0	-	-	-	-	0	0	0	0	0	-	-	
CV (%)	0	-	0	0	0	0	-	-	-	-	0	0	0	0	0	-	-	
Swartzia madagascariensis	47.2	-	92.6	40.2	16.3	22.1	-	-	-	-	116	0.57	0.46	82.4	51	-	-	Sidiimorou et al., 2016
Average	47.2	-	92.6	40.2	16.3	22.1	-	-	-	-	116	0.57	0.46	82.4	51	-	-	Sidiimorou et al., 2016 (n=1)
Minimum	47.2	-	92.6	40.2	16.3	22.1	-	-	-	-	116	0.57	0.46	82.4	51	-	-	
Maximum	47.2	-	92.6	40.2	16.3	22.1	-	-	-	-	116	0.57	0.46	82.4	51	-	-	
Standard deviation	0	-	0	0	0	0	-	-	-	-	0	0	0	0	0	-	-	
CV (%)	0	-	0	0	0	0	-	-	-	-	0	0	0	0	0	-	-	
Vitellaria paradoxa	40.2	-	96.8	38.8	11.1	26.9	-	-	-	-	68	0.5	0.38	56.5	55.6	-	-	Sidiimorou et al., 2016
Average	40.2	-	96.8	38.8	11.1	26.9	-	-	-	-	68	0.5	0.38	56.5	55.6	-	-	Sidiimorou et al., 2016 (n=1)
Minimum	40.2	-	96.8	38.8	11.1	26.9	-	-	-	-	68	0.5	0.38	56.5	55.6	-	-	
Maximum	40.2	-	96.8	38.8	11.1	26.9	-	-	-	-	68	0.5	0.38	56.5	55.6	-	-	
Standard deviation	0	-	0	0	0	0	-	-	-	-	0	0	0	0	0	-	-	
CV (%)	0	-	0	0	0	0	-	-	-	-	0	0	0	0	0	-	-	
Xeroderris stuhlmannii	44.8	-	97	47.7	17.3	40	-	-	-	-	126	0.62	0.52	77.1	73.4	-	-	Sidiimorou et al., 2016
Average	44.8	-	97	47.7	17.3	40	-	-	-	-	126	0.62	0.52	77.1	73.4	-	-	Sidiimorou et al., 2016 (n=1)
Minimum	44.8	-	97	47.7	17.3	40	-	-	-	-	126	0.62	0.52	77.1	73.4	-	-	
Maximum	44.8	-	97	47.7	17.3	40	-	-	-	-	126	0.62	0.52	77.1	73.4	-	-	
Standard deviation	0	-	0	0	0	0	-	-	-	-	0	0	0	0	0	-	-	

CV (%)	0	-	0	0	0	0	-	-	-	-	0	0	0	0	0	-	-	
Acacia melifera	94.4199	6.5846	-	-	-	22.0301	8.8747	-	-	-	-	-	-	-	-	-	-	Mawussi et al., 2022
Average	94.42	6.58	-	-	-	22.03	8.87	-	-	-	-	-	-	-	-	-	-	Mawussi et al., 2022 (n=1)
Minimum	94.42	6.58	-	-	-	22.03	8.87	-	-	-	-	-	-	-	-	-	-	
Maximum	94.42	6.58	-	-	-	22.03	8.87	-	-	-	-	-	-	-	-	-	-	
Standard deviation	0	0	-	-	-	0	0	-	-	-	-	-	-	-	-	-	-	
CV (%)	0	0	-	-	-	0	0	-	-	-	-	-	-	-	-	-	-	
Adansonia digitata	94.7411	2.2246	-	-	-	8.3298	10.5097	-	-	-	-	-	-	-	-	-	-	Mawussi et al., 2022
Average	94.74	2.22	-	-	-	8.33	10.51	-	-	-	-	-	-	-	-	-	-	Mawussi et al., 2022 (n=1)
Minimum	94.74	2.22	-	-	-	8.33	10.51	-	-	-	-	-	-	-	-	-	-	
Maximum	94.74	2.22	-	-	-	8.33	10.51	-	-	-	-	-	-	-	-	-	-	
Standard deviation	0	0	-	-	-	0	0	-	-	-	-	-	-	-	-	-	-	
CV (%)	0	0	-	-	-	0	0	-	-	-	-	-	-	-	-	-	-	
Albizzia spp	94.6201	1.5352	-	-	-	20.9633	5.6227	-	-	-	-	-	-	-	-	-	-	Mawussi et al., 2022
Average	94.62	1.54	-	-	-	20.96	5.62	-	-	-	-	-	-	-	-	-	-	Mawussi et al., 2022 (n=1)
Minimum	94.62	1.54	-	-	-	20.96	5.62	-	-	-	-	-	-	-	-	-	-	
Maximum	94.62	1.54	-	-	-	20.96	5.62	-	-	-	-	-	-	-	-	-	-	
Standard deviation	0	0	-	-	-	0	0	-	-	-	-	-	-	-	-	-	-	
CV (%)	0	0	-	-	-	0	0	-	-	-	-	-	-	-	-	-	-	
Andropogon spp	93.8367	2.2493	-	-	-	17.1871	13.8819	-	-	-	-	-	-	-	-	-	-	Mawussi et al., 2022
Average	93.84	2.25	-	-	-	17.19	13.88	-	-	-	-	-	-	-	-	-	-	Mawussi et al., 2022 (n=1)
Minimum	93.84	2.25	-	-	-	17.19	13.88	-	-	-	-	-	-	-	-	-	-	
Maximum	93.84	2.25	-	-	-	17.19	13.88	-	-	-	-	-	-	-	-	-	-	
Standard deviation	0	0	-	-	-	0	0	-	-	-	-	-	-	-	-	-	-	
CV (%)	0	0	-	-	-	0	0	-	-	-	-	-	-	-	-	-	-	
Andropogon tectorum	93.6789	5.82	-	-	-	24.8944	5.6052	-	-	-	-	-	-	-	-	-	-	Mawussi et al., 2022
Average	93.68	5.82	-	-	-	24.89	5.61	-	-	-	-	-	-	-	-	-	-	Mawussi et al., 2022 (n=1)
Minimum	93.68	5.82	-	-	-	24.89	5.61	-	-	-	-	-	-	-	-	-	-	
Maximum	93.68	5.82	-	-	-	24.89	5.61	-	-	-	-	-	-	-	-	-	-	
Standard deviation	0	0	-	-	-	0	0	-	-	-	-	-	-	-	-	-	-	
CV (%)	0	0	-	-	-	0	0	-	-	-	-	-	-	-	-	-	-	
Bridelia ferrugineae	94.3132	3.5599	-	-	-	21.4954	7.0186	-	-	-	-	-	-	-	-	-	-	Mawussi et al., 2022
Average	94.31	3.56	-	-	-	21.5	7.02	-	-	-	-	-	-	-	-	-	-	Mawussi et al., 2022 (n=1)
Minimum	94.31	3.56	-	-	-	21.5	7.02	-	-	-	-	-	-	-	-	-	-	
Maximum	94.31	3.56	-	-	-	21.5	7.02	-	-	-	-	-	-	-	-	-	-	
Standard deviation	0	0	-	-	-	0	0	-	-	-	-	-	-	-	-	-	-	
CV (%)	0	0	-	-	-	0	0	-	-	-	-	-	-	-	-	-	-	
Cajanus cajan	94.1177	8.11	-	-	-	14.2362	7.3456	-	-	-	-	-	-	-	-	-	-	Mawussi et al., 2022
Average	94.12	8.11	-	-	-	14.24	7.35	-	-	-	-	-	-	-	-	-	-	Mawussi et al., 2022 (n=1)
Minimum	94.12	8.11	-	-	-	14.24	7.35	-	-	-	-	-	-	-	-	-	-	
Maximum	94.12	8.11	-	-	-	14.24	7.35	-	-	-	-	-	-	-	-	-	-	
Standard deviation	0	0	-	-	-	0	0	-	-	-	-	-	-	-	-	-	-	
CV (%)	0	0	-	-	-	0	0	-	-	-	-	-	-	-	-	-	-	

Cassia sieberiana (Leaves)	-	-	-	50.1	13.3	-	4.3	-	-	7.15	-	-	-	66.5	55.4	-	-	Amole et al., 2022
Average	-	-	-	50.1	13.3	-	4.3	-	-	7.15	-	-	-	66.5	55.4	-	-	Amole et al., 2022 (n=1)
Minimum	-	-	-	50.1	13.3	-	4.3	-	-	7.15	-	-	-	66.5	55.4	-	-	
Maximum	-	-	-	50.1	13.3	-	4.3	-	-	7.15	-	-	-	66.5	55.4	-	-	
Standard deviation	-	-	-	0	0	-	0	-	-	0	-	-	-	0	0	-	-	
CV (%)	-	-	-	0	0	-	0	-	-	0	-	-	-	0	0	-	-	
Cassia sieberiana (Pods)	-	-	-	61	8.1	-	4.8	-	-	9.08	-	-	-	32.5	31.6	-	-	Amole et al., 2022
Average	-	-	-	61	8.1	-	4.8	-	-	9.08	-	-	-	32.5	31.6	-	-	Amole et al., 2022 (n=1)
Minimum	-	-	-	61	8.1	-	4.8	-	-	9.08	-	-	-	32.5	31.6	-	-	
Maximum	-	-	-	61	8.1	-	4.8	-	-	9.08	-	-	-	32.5	31.6	-	-	
Standard deviation	-	-	-	0	0	-	0	-	-	0	-	-	-	0	0	-	-	
CV (%)	-	-	-	0	0	-	0	-	-	0	-	-	-	0	0	-	-	
Cordia africana	95.1152	2.8523	-	-	-	8.3305	8.5592	-	-	-	-	-	-	-	-	-	-	Mawussi et al., 2022
Average	95.12	2.85	-	-	-	8.33	8.56	-	-	-	-	-	-	-	-	-	-	Mawussi et al., 2022 (n=1)
Minimum	95.12	2.85	-	-	-	8.33	8.56	-	-	-	-	-	-	-	-	-	-	
Maximum	95.12	2.85	-	-	-	8.33	8.56	-	-	-	-	-	-	-	-	-	-	
Standard deviation	0	0	-	-	-	0	0	-	-	-	-	-	-	-	-	-	-	
CV (%)	0	0	-	-	-	0	0	-	-	-	-	-	-	-	-	-	-	
Cordia mixa L (Leaves)	-	-	-	42.3	15.1	-	14.1	-	-	5.54	-	-	-	59.5	67.2	-	-	Amole et al., 2022
Average	-	-	-	42.3	15.1	-	14.1	-	-	5.54	-	-	-	59.5	67.2	-	-	Amole et al., 2022 (n=1)
Minimum	-	-	-	42.3	15.1	-	14.1	-	-	5.54	-	-	-	59.5	67.2	-	-	
Maximum	-	-	-	42.3	15.1	-	14.1	-	-	5.54	-	-	-	59.5	67.2	-	-	
Standard deviation	-	-	-	0	0	-	0	-	-	0	-	-	-	0	0	-	-	
CV (%)	-	-	-	0	0	-	0	-	-	0	-	-	-	0	0	-	-	
Crotalaria pallida	94.1345	3.4863	-	-	-	8.1229	12.9276	-	-	-	-	-	-	-	-	-	-	Mawussi et al., 2022
Average	94.13	3.49	-	-	-	8.12	12.93	-	-	-	-	-	-	-	-	-	-	Mawussi et al., 2022 (n=1)
Minimum	94.13	3.49	-	-	-	8.12	12.93	-	-	-	-	-	-	-	-	-	-	
Maximum	94.13	3.49	-	-	-	8.12	12.93	-	-	-	-	-	-	-	-	-	-	
Standard deviation	0	0	-	-	-	0	0	-	-	-	-	-	-	-	-	-	-	
CV (%)	0	0	-	-	-	0	0	-	-	-	-	-	-	-	-	-	-	
Desmodium tortuosum	93.2905	2.5874	-	-	-	10.6784	12.8166	-	-	-	-	-	-	-	-	-	-	Mawussi et al., 2022
Average	93.29	2.59	-	-	-	10.68	12.82	-	-	-	-	-	-	-	-	-	-	Mawussi et al., 2022 (n=1)
Minimum	93.29	2.59	-	-	-	10.68	12.82	-	-	-	-	-	-	-	-	-	-	
Maximum	93.29	2.59	-	-	-	10.68	12.82	-	-	-	-	-	-	-	-	-	-	
Standard deviation	0	0	-	-	-	0	0	-	-	-	-	-	-	-	-	-	-	
CV (%)	0	0	-	-	-	0	0	-	-	-	-	-	-	-	-	-	-	
Dialium guineense [rural]	89.35	1.85	90.58	-	9.04	8.66	9.42	-	-	-	-	-	-	-	-	-	-	Okoli et al., 2024
Average	89.35	1.85	90.58	-	9.04	8.66	9.42	-	-	-	-	-	-	-	-	-	-	Okoli et al., 2024 (n=1)
Minimum	89.35	1.85	90.58	-	9.04	8.66	9.42	-	-	-	-	-	-	-	-	-	-	
Maximum	89.35	1.85	90.58	-	9.04	8.66	9.42	-	-	-	-	-	-	-	-	-	-	

Standard deviation	0	0	0	-	0	0	0	-	-	-	-	-	-	-	-	-	-	
CV (%)	0	0	0	-	0	0	0	-	-	-	-	-	-	-	-	-	-	
Dialium guineense [peri-urban]	89.04	2.18	89.98	-	9.61	17.69	10.02	-	-	-	-	-	-	-	-	-	-	Okoli et al., 2024
Average	89.04	2.18	89.98	-	9.61	17.69	10.02	-	-	-	-	-	-	-	-	-	-	Okoli et al., 2024 (n=1)
Minimum	89.04	2.18	89.98	-	9.61	17.69	10.02	-	-	-	-	-	-	-	-	-	-	
Maximum	89.04	2.18	89.98	-	9.61	17.69	10.02	-	-	-	-	-	-	-	-	-	-	
Standard deviation	0	0	0	-	0	0	0	-	-	-	-	-	-	-	-	-	-	
CV (%)	0	0	0	-	0	0	0	-	-	-	-	-	-	-	-	-	-	
Dichapetalum madagascariense	93.5373	2.3796	-	-	-	10.129	8.8379	-	-	-	-	-	-	-	-	-	-	Mawussi et al., 2022
Average	93.54	2.38	-	-	-	10.13	8.84	-	-	-	-	-	-	-	-	-	-	Mawussi et al., 2022 (n=1)
Minimum	93.54	2.38	-	-	-	10.13	8.84	-	-	-	-	-	-	-	-	-	-	
Maximum	93.54	2.38	-	-	-	10.13	8.84	-	-	-	-	-	-	-	-	-	-	
Standard deviation	0	0	-	-	-	0	0	-	-	-	-	-	-	-	-	-	-	
CV (%)	0	0	-	-	-	0	0	-	-	-	-	-	-	-	-	-	-	
Digitaria alata	99	1	-	-	6.41	9.5	10.5	-	-	-	-	-	-	-	-	0.65	3.77	Muftau and Musa, 2020
Average	99	1	-	-	6.41	9.5	10.5	-	-	-	-	-	-	-	-	0.65	3.77	Muftau and Musa, 2020 (n=1)
Minimum	99	1	-	-	6.41	9.5	10.5	-	-	-	-	-	-	-	-	0.65	3.77	
Maximum	99	1	-	-	6.41	9.5	10.5	-	-	-	-	-	-	-	-	0.65	3.77	
Standard deviation	0	0	-	-	0	0	0	-	-	-	-	-	-	-	-	0	0	
CV (%)	0	0	-	-	0	0	0	-	-	-	-	-	-	-	-	0	0	
Elaeis guineensis [rural]	85.29	2.18	88.36	-	5.96	19.84	11.64	-	-	-	-	-	-	-	-	-	-	Okoli et al., 2024
Average	85.29	2.18	88.36	-	5.96	19.84	11.64	-	-	-	-	-	-	-	-	-	-	Okoli et al., 2024 (n=1)
Minimum	85.29	2.18	88.36	-	5.96	19.84	11.64	-	-	-	-	-	-	-	-	-	-	
Maximum	85.29	2.18	88.36	-	5.96	19.84	11.64	-	-	-	-	-	-	-	-	-	-	
Standard deviation	0	0	0	-	0	0	0	-	-	-	-	-	-	-	-	-	-	
CV (%)	0	0	0	-	0	0	0	-	-	-	-	-	-	-	-	-	-	
Elaeis guineensis [peri-urban]	86.35	2.25	90.39	-	9.12	19.71	9.61	-	-	-	-	-	-	-	-	-	-	Okoli et al., 2024
Average	86.35	2.25	90.39	-	9.12	19.71	9.61	-	-	-	-	-	-	-	-	-	-	Okoli et al., 2024 (n=1)
Minimum	86.35	2.25	90.39	-	9.12	19.71	9.61	-	-	-	-	-	-	-	-	-	-	
Maximum	86.35	2.25	90.39	-	9.12	19.71	9.61	-	-	-	-	-	-	-	-	-	-	
Standard deviation	0	0	0	-	0	0	0	-	-	-	-	-	-	-	-	-	-	
CV (%)	0	0	0	-	0	0	0	-	-	-	-	-	-	-	-	-	-	
Euphorbia convolvuloides	98.33	3.5	-	-	11.93	9.5	5.5	-	-	-	-	-	-	-	-	0.85	4.05	Muftau and Musa, 2020
Average	98.33	3.5	-	-	11.93	9.5	5.5	-	-	-	-	-	-	-	-	0.85	4.05	Muftau and Musa, 2020 (n=1)
Minimum	98.33	3.5	-	-	11.93	9.5	5.5	-	-	-	-	-	-	-	-	0.85	4.05	
Maximum	98.33	3.5	-	-	11.93	9.5	5.5	-	-	-	-	-	-	-	-	0.85	4.05	
Standard deviation	0	0	-	-	0	0	0	-	-	-	-	-	-	-	-	0	0	
CV (%)	0	0	-	-	0	0	0	-	-	-	-	-	-	-	-	0	0	
Fagara xathozilodis	94.1453	0.898	-	-	-	10.1946	9.6578	-	-	-	-	-	-	-	-	-	-	Mawussi et al., 2022

Average	94.15	0.9	-	-	-	10.19	9.66	-	-	-	-	-	-	-	-	-	-	Mawussi et al., 2022 (n=1)
Minimum	94.15	0.9	-	-	-	10.19	9.66	-	-	-	-	-	-	-	-	-	-	
Maximum	94.15	0.9	-	-	-	10.19	9.66	-	-	-	-	-	-	-	-	-	-	
Standard deviation	0	0	-	-	-	0	0	-	-	-	-	-	-	-	-	-	-	
CV (%)	0	0	-	-	-	0	0	-	-	-	-	-	-	-	-	-	-	
Imperata cylindrica	93.9399	0.8476	-	-	-	8.7011	30.3293	-	-	-	-	-	-	-	-	-	-	Mawussi et al., 2022
Imperata cylindrica	-	-	-	-	2.88	4.5	-	-	-	-	-	-	-	-	-	0.049	0.062	Amegnaglo et al., 2018
Average	93.94	0.85	-	-	2.88	6.6	30.33	-	-	-	-	-	-	-	-	0.05	0.06	Mawussi et al., 2022; Amegnaglo et al., 2018 (n=2)
Minimum	93.94	0.85	-	-	2.88	4.5	30.33	-	-	-	-	-	-	-	-	0.05	0.06	
Maximum	93.94	0.85	-	-	2.88	8.7	30.33	-	-	-	-	-	-	-	-	0.05	0.06	
Standard deviation	0	0	-	-	0	2.97	0	-	-	-	-	-	-	-	-	0	0	
CV (%)	0	0	-	-	0	45.01	0	-	-	-	-	-	-	-	-	0	0	
Indigofera dendroides	94.0241	2.6742	-	-	-	8.4478	15.6818	-	-	-	-	-	-	-	-	-	-	Mawussi et al., 2022
Average	94.02	2.67	-	-	-	8.45	15.68	-	-	-	-	-	-	-	-	-	-	Mawussi et al., 2022 (n=1)
Minimum	94.02	2.67	-	-	-	8.45	15.68	-	-	-	-	-	-	-	-	-	-	
Maximum	94.02	2.67	-	-	-	8.45	15.68	-	-	-	-	-	-	-	-	-	-	
Standard deviation	0	0	-	-	-	0	0	-	-	-	-	-	-	-	-	-	-	
CV (%)	0	0	-	-	-	0	0	-	-	-	-	-	-	-	-	-	-	
Indigofera spp	94.2443	3.5349	-	-	-	17.2243	9.4317	-	-	-	-	-	-	-	-	-	-	Mawussi et al., 2022
Average	94.24	3.53	-	-	-	17.22	9.43	-	-	-	-	-	-	-	-	-	-	Mawussi et al., 2022 (n=1)
Minimum	94.24	3.53	-	-	-	17.22	9.43	-	-	-	-	-	-	-	-	-	-	
Maximum	94.24	3.53	-	-	-	17.22	9.43	-	-	-	-	-	-	-	-	-	-	
Standard deviation	0	0	-	-	-	0	0	-	-	-	-	-	-	-	-	-	-	
CV (%)	0	0	-	-	-	0	0	-	-	-	-	-	-	-	-	-	-	
Ipomoea kotschyana	94.5	2	-	-	4.55	20.5	11.5	-	-	-	-	-	-	-	-	0.6	4.78	Muftau and Musa, 2020
Average	94.5	2	-	-	4.55	20.5	11.5	-	-	-	-	-	-	-	-	0.6	4.78	Muftau and Musa, 2020 (n=1)
Minimum	94.5	2	-	-	4.55	20.5	11.5	-	-	-	-	-	-	-	-	0.6	4.78	
Maximum	94.5	2	-	-	4.55	20.5	11.5	-	-	-	-	-	-	-	-	0.6	4.78	
Standard deviation	0	0	-	-	0	0	0	-	-	-	-	-	-	-	-	0	0	
CV (%)	0	0	-	-	0	0	0	-	-	-	-	-	-	-	-	0	0	
Malotus oppositifolius	94.7688	4.5626	-	-	-	10.6999	9.4666	-	-	-	-	-	-	-	-	-	-	Mawussi et al., 2022
Average	94.77	4.56	-	-	-	10.7	9.47	-	-	-	-	-	-	-	-	-	-	Mawussi et al., 2022 (n=1)
Minimum	94.77	4.56	-	-	-	10.7	9.47	-	-	-	-	-	-	-	-	-	-	
Maximum	94.77	4.56	-	-	-	10.7	9.47	-	-	-	-	-	-	-	-	-	-	
Standard deviation	0	0	-	-	-	0	0	-	-	-	-	-	-	-	-	-	-	
CV (%)	0	0	-	-	-	0	0	-	-	-	-	-	-	-	-	-	-	
Melanthera scandens	93.219	3.0852	-	-	-	9.2069	14.8654	-	-	-	-	-	-	-	-	-	-	Mawussi et al., 2022
Average	93.22	3.09	-	-	-	9.21	14.87	-	-	-	-	-	-	-	-	-	-	Mawussi et al., 2022 (n=1)
Minimum	93.22	3.09	-	-	-	9.21	14.87	-	-	-	-	-	-	-	-	-	-	
Maximum	93.22	3.09	-	-	-	9.21	14.87	-	-	-	-	-	-	-	-	-	-	
Standard deviation	0	0	-	-	-	0	0	-	-	-	-	-	-	-	-	-	-	
CV (%)	0	0	-	-	-	0	0	-	-	-	-	-	-	-	-	-	-	

Mitracarpus hiristus	95.5	1.5	-	-	6.21	15.5	8.5	-	-	-	-	-	-	-	-	0.55	4.14	Muftau and Musa, 2020
Average	95.5	1.5	-	-	6.21	15.5	8.5	-	-	-	-	-	-	-	-	0.55	4.14	Muftau and Musa, 2020 (n=1)
Minimum	95.5	1.5	-	-	6.21	15.5	8.5	-	-	-	-	-	-	-	-	0.55	4.14	
Maximum	95.5	1.5	-	-	6.21	15.5	8.5	-	-	-	-	-	-	-	-	0.55	4.14	
Standard deviation	0	0	-	-	0	0	0	-	-	-	-	-	-	-	-	0	0	
CV (%)	0	0	-	-	0	0	0	-	-	-	-	-	-	-	-	0	0	
Monechma ciliatum	94.5	2	-	-	5.95	15.5	10.5	-	-	-	-	-	-	-	-	0.75	5.18	Muftau and Musa, 2020
Average	94.5	2	-	-	5.95	15.5	10.5	-	-	-	-	-	-	-	-	0.75	5.18	Muftau and Musa, 2020 (n=1)
Minimum	94.5	2	-	-	5.95	15.5	10.5	-	-	-	-	-	-	-	-	0.75	5.18	
Maximum	94.5	2	-	-	5.95	15.5	10.5	-	-	-	-	-	-	-	-	0.75	5.18	
Standard deviation	0	0	-	-	0	0	0	-	-	-	-	-	-	-	-	0	0	
CV (%)	0	0	-	-	0	0	0	-	-	-	-	-	-	-	-	0	0	
Mucuna pruriens	93.2777	3.33	-	-	-	15.298	9.2439	-	-	-	-	-	-	-	-	-	-	Mawussi et al., 2022
Average	93.28	3.33	-	-	-	15.3	9.24	-	-	-	-	-	-	-	-	-	-	Mawussi et al., 2022 (n=1)
Minimum	93.28	3.33	-	-	-	15.3	9.24	-	-	-	-	-	-	-	-	-	-	
Maximum	93.28	3.33	-	-	-	15.3	9.24	-	-	-	-	-	-	-	-	-	-	
Standard deviation	0	0	-	-	-	0	0	-	-	-	-	-	-	-	-	-	-	
CV (%)	0	0	-	-	-	0	0	-	-	-	-	-	-	-	-	-	-	
Musa acuminata	93.7081	3.3041	-	-	-	24.6327	11.7349	-	-	-	-	-	-	-	-	-	-	Mawussi et al., 2022
Average	93.71	3.3	-	-	-	24.63	11.73	-	-	-	-	-	-	-	-	-	-	Mawussi et al., 2022 (n=1)
Minimum	93.71	3.3	-	-	-	24.63	11.73	-	-	-	-	-	-	-	-	-	-	
Maximum	93.71	3.3	-	-	-	24.63	11.73	-	-	-	-	-	-	-	-	-	-	
Standard deviation	0	0	-	-	-	0	0	-	-	-	-	-	-	-	-	-	-	
CV (%)	0	0	-	-	-	0	0	-	-	-	-	-	-	-	-	-	-	
Pannicum maximum	93.4858	1.7524	-	-	-	8.5621	10.6267	-	-	-	-	-	-	-	-	-	-	Mawussi et al., 2022
Average	93.49	1.75	-	-	-	8.56	10.63	-	-	-	-	-	-	-	-	-	-	Mawussi et al., 2022 (n=1)
Minimum	93.49	1.75	-	-	-	8.56	10.63	-	-	-	-	-	-	-	-	-	-	
Maximum	93.49	1.75	-	-	-	8.56	10.63	-	-	-	-	-	-	-	-	-	-	
Standard deviation	0	0	-	-	-	0	0	-	-	-	-	-	-	-	-	-	-	
CV (%)	0	0	-	-	-	0	0	-	-	-	-	-	-	-	-	-	-	
Paullinia pinnata	94.8842	2.142	-	-	-	25.3648	5.0278	-	-	-	-	-	-	-	-	-	-	Mawussi et al., 2022
Average	94.88	2.14	-	-	-	25.36	5.03	-	-	-	-	-	-	-	-	-	-	Mawussi et al., 2022 (n=1)
Minimum	94.88	2.14	-	-	-	25.36	5.03	-	-	-	-	-	-	-	-	-	-	
Maximum	94.88	2.14	-	-	-	25.36	5.03	-	-	-	-	-	-	-	-	-	-	
Standard deviation	0	0	-	-	-	0	0	-	-	-	-	-	-	-	-	-	-	
CV (%)	0	0	-	-	-	0	0	-	-	-	-	-	-	-	-	-	-	
Pennisetum purpureum [rural]	85.81	1.98	90.79	-	9.11	22.28	9.21	-	-	-	-	-	-	-	-	-	-	Okoli et al., 2024
Average	85.81	1.98	90.79	-	9.11	22.28	9.21	-	-	-	-	-	-	-	-	-	-	Okoli et al., 2024 (n=1)
Minimum	85.81	1.98	90.79	-	9.11	22.28	9.21	-	-	-	-	-	-	-	-	-	-	
Maximum	85.81	1.98	90.79	-	9.11	22.28	9.21	-	-	-	-	-	-	-	-	-	-	
Standard deviation	0	0	0	-	0	0	0	-	-	-	-	-	-	-	-	-	-	
CV (%)	0	0	0	-	0	0	0	-	-	-	-	-	-	-	-	-	-	

Persea americana [rural]	86.78	2.01	89.84	-	8.74	20.74	10.16	-	-	-	-	-	-	-	-	-	-	Okoli et al., 2024
Average	86.78	2.01	89.84	-	8.74	20.74	10.16	-	-	-	-	-	-	-	-	-	-	Okoli et al., 2024 (n=1)
Minimum	86.78	2.01	89.84	-	8.74	20.74	10.16	-	-	-	-	-	-	-	-	-	-	
Maximum	86.78	2.01	89.84	-	8.74	20.74	10.16	-	-	-	-	-	-	-	-	-	-	
Standard deviation	0	0	0	-	0	0	0	-	-	-	-	-	-	-	-	-	-	
CV (%)	0	0	0	-	0	0	0	-	-	-	-	-	-	-	-	-	-	
Persea americana [peri-urban]	86.86	3.85	91.67	-	10.86	10.15	8.33	-	-	-	-	-	-	-	-	-	-	Okoli et al., 2024
Average	86.86	3.85	91.67	-	10.86	10.15	8.33	-	-	-	-	-	-	-	-	-	-	Okoli et al., 2024 (n=1)
Minimum	86.86	3.85	91.67	-	10.86	10.15	8.33	-	-	-	-	-	-	-	-	-	-	
Maximum	86.86	3.85	91.67	-	10.86	10.15	8.33	-	-	-	-	-	-	-	-	-	-	
Standard deviation	0	0	0	-	0	0	0	-	-	-	-	-	-	-	-	-	-	
CV (%)	0	0	0	-	0	0	0	-	-	-	-	-	-	-	-	-	-	
Philiostigma tonenji (Pods)	-	-	-	46.9	7.7	-	11.6	-	-	6.65	-	-	-	55.5	23.9	-	-	Amole et al., 2022
Average	-	-	-	46.9	7.7	-	11.6	-	-	6.65	-	-	-	55.5	23.9	-	-	Amole et al., 2022 (n=1)
Minimum	-	-	-	46.9	7.7	-	11.6	-	-	6.65	-	-	-	55.5	23.9	-	-	
Maximum	-	-	-	46.9	7.7	-	11.6	-	-	6.65	-	-	-	55.5	23.9	-	-	
Standard deviation	-	-	-	0	0	-	0	-	-	0	-	-	-	0	0	-	-	
CV (%)	-	-	-	0	0	-	0	-	-	0	-	-	-	0	0	-	-	
Pithecellobium dulce	93.5351	4.0747	-	-	-	15.7465	8.2122	-	-	-	-	-	-	-	-	-	-	Mawussi et al., 2022
Average	93.54	4.07	-	-	-	15.75	8.21	-	-	-	-	-	-	-	-	-	-	Mawussi et al., 2022 (n=1)
Minimum	93.54	4.07	-	-	-	15.75	8.21	-	-	-	-	-	-	-	-	-	-	
Maximum	93.54	4.07	-	-	-	15.75	8.21	-	-	-	-	-	-	-	-	-	-	
Standard deviation	0	0	-	-	-	0	0	-	-	-	-	-	-	-	-	-	-	
CV (%)	0	0	-	-	-	0	0	-	-	-	-	-	-	-	-	-	-	
Pterocarpus santalinoides [peri-urban]	88.64	6.11	91.44	-	14.06	9.95	8.56	-	-	-	-	-	-	-	-	-	-	Okoli et al., 2024
Average	88.64	6.11	91.44	-	14.06	9.95	8.56	-	-	-	-	-	-	-	-	-	-	Okoli et al., 2024 (n=1)
Minimum	88.64	6.11	91.44	-	14.06	9.95	8.56	-	-	-	-	-	-	-	-	-	-	
Maximum	88.64	6.11	91.44	-	14.06	9.95	8.56	-	-	-	-	-	-	-	-	-	-	
Standard deviation	0	0	0	-	0	0	0	-	-	-	-	-	-	-	-	-	-	
CV (%)	0	0	0	-	0	0	0	-	-	-	-	-	-	-	-	-	-	
Sorghum spp	93.0148	3.41	-	-	-	16.4231	8.4117	-	-	-	-	-	-	-	-	-	-	Mawussi et al., 2022
Average	93.01	3.41	-	-	-	16.42	8.41	-	-	-	-	-	-	-	-	-	-	Mawussi et al., 2022 (n=1)
Minimum	93.01	3.41	-	-	-	16.42	8.41	-	-	-	-	-	-	-	-	-	-	
Maximum	93.01	3.41	-	-	-	16.42	8.41	-	-	-	-	-	-	-	-	-	-	
Standard deviation	0	0	-	-	-	0	0	-	-	-	-	-	-	-	-	-	-	
CV (%)	0	0	-	-	-	0	0	-	-	-	-	-	-	-	-	-	-	
Spondias monbin	90.5699	1.9375	-	-	-	18.3007	9.2041	-	-	-	-	-	-	-	-	-	-	Mawussi et al., 2022
Average	90.57	1.94	-	-	-	18.3	9.2	-	-	-	-	-	-	-	-	-	-	Mawussi et al., 2022 (n=1)
Minimum	90.57	1.94	-	-	-	18.3	9.2	-	-	-	-	-	-	-	-	-	-	

Maximum	90.57	1.94	-	-	-	18.3	9.2	-	-	-	-	-	-	-	-	-	-	
Standard deviation	0	0	-	-	-	0	0	-	-	-	-	-	-	-	-	-	-	
CV (%)	0	0	-	-	-	0	0	-	-	-	-	-	-	-	-	-	-	
Sporobolus pyramidalis	94.3005	1.4752	-	-	-	19.6329	13.4695	-	-	-	-	-	-	-	-	-	-	Mawussi et al., 2022
Sporobolus pyramidalis	-	-	-	-	1.94	5.8	-	-	-	-	-	-	-	-	-	0.037	0.036	Amegnaglo et al., 2018
Average	94.3	1.48	-	-	1.94	12.72	13.47	-	-	-	-	-	-	-	-	0.04	0.04	Mawussi et al., 2022; Amegnaglo et al., 2018 (n=2)
Minimum	94.3	1.48	-	-	1.94	5.8	13.47	-	-	-	-	-	-	-	-	0.04	0.04	
Maximum	94.3	1.48	-	-	1.94	19.63	13.47	-	-	-	-	-	-	-	-	0.04	0.04	
Standard deviation	0	0	-	-	0	9.78	0	-	-	-	-	-	-	-	-	0	0	
CV (%)	0	0	-	-	0	76.92	0	-	-	-	-	-	-	-	-	0	0	
Stylochaeton lancifolius	97.7622	0.8771	-	-	-	15.5881	7.4988	-	-	-	-	-	-	-	-	-	-	Mawussi et al., 2022
Average	97.76	0.88	-	-	-	15.59	7.5	-	-	-	-	-	-	-	-	-	-	Mawussi et al., 2022 (n=1)
Minimum	97.76	0.88	-	-	-	15.59	7.5	-	-	-	-	-	-	-	-	-	-	
Maximum	97.76	0.88	-	-	-	15.59	7.5	-	-	-	-	-	-	-	-	-	-	
Standard deviation	0	0	-	-	-	0	0	-	-	-	-	-	-	-	-	-	-	
CV (%)	0	0	-	-	-	0	0	-	-	-	-	-	-	-	-	-	-	
Tephrosia villosa	93.4301	3.2422	-	-	-	8.3076	7.8538	-	-	-	-	-	-	-	-	-	-	Mawussi et al., 2022
Average	93.43	3.24	-	-	-	8.31	7.85	-	-	-	-	-	-	-	-	-	-	Mawussi et al., 2022 (n=1)
Minimum	93.43	3.24	-	-	-	8.31	7.85	-	-	-	-	-	-	-	-	-	-	
Maximum	93.43	3.24	-	-	-	8.31	7.85	-	-	-	-	-	-	-	-	-	-	
Standard deviation	0	0	-	-	-	0	0	-	-	-	-	-	-	-	-	-	-	
CV (%)	0	0	-	-	-	0	0	-	-	-	-	-	-	-	-	-	-	
Ruzi grass [4 WAP, first harvest, Northern]	-	-	-	65.3	18.6	-	-	-	-	8.3	-	-	-	51.2	-	-	-	Amole et al., 2022
Average	-	-	-	65.3	18.6	-	-	-	-	8.3	-	-	-	51.2	-	-	-	Amole et al., 2022 (n=1)
Minimum	-	-	-	65.3	18.6	-	-	-	-	8.3	-	-	-	51.2	-	-	-	
Maximum	-	-	-	65.3	18.6	-	-	-	-	8.3	-	-	-	51.2	-	-	-	
Standard deviation	-	-	-	0	0	-	-	-	-	0	-	-	-	0	-	-	-	
CV (%)	-	-	-	0	0	-	-	-	-	0	-	-	-	0	-	-	-	
Ruzi grass [4 WAP, first harvest, Upper East]	-	-	-	59.7	19.6	-	-	-	-	4.3	-	-	-	54.9	-	-	-	Amole et al., 2022
Average	-	-	-	59.7	19.6	-	-	-	-	4.3	-	-	-	54.9	-	-	-	Amole et al., 2022 (n=1)
Minimum	-	-	-	59.7	19.6	-	-	-	-	4.3	-	-	-	54.9	-	-	-	
Maximum	-	-	-	59.7	19.6	-	-	-	-	4.3	-	-	-	54.9	-	-	-	
Standard deviation	-	-	-	0	0	-	-	-	-	0	-	-	-	0	-	-	-	
CV (%)	-	-	-	0	0	-	-	-	-	0	-	-	-	0	-	-	-	
Ruzi grass [4 WAP, first regrowth, Northern]	-	-	-	61.6	15.8	-	-	-	-	7	-	-	-	55.8	-	-	-	Amole et al., 2022
Average	-	-	-	61.6	15.8	-	-	-	-	7	-	-	-	55.8	-	-	-	Amole et al., 2022 (n=1)
Minimum	-	-	-	61.6	15.8	-	-	-	-	7	-	-	-	55.8	-	-	-	
Maximum	-	-	-	61.6	15.8	-	-	-	-	7	-	-	-	55.8	-	-	-	
Standard deviation	-	-	-	0	0	-	-	-	-	0	-	-	-	0	-	-	-	
CV (%)	-	-	-	0	0	-	-	-	-	0	-	-	-	0	-	-	-	

Ruzi grass [4 WAP, first regrowth, Upper East]	-	-	-	55.1	11.1	-	-	-	-	6.5	-	-	-	56.4	-	-	-	Amole et al., 2022
Average	-	-	-	55.1	11.1	-	-	-	-	6.5	-	-	-	56.4	-	-	-	Amole et al., 2022 (n=1)
Minimum	-	-	-	55.1	11.1	-	-	-	-	6.5	-	-	-	56.4	-	-	-	
Maximum	-	-	-	55.1	11.1	-	-	-	-	6.5	-	-	-	56.4	-	-	-	
Standard deviation	-	-	-	0	0	-	-	-	-	0	-	-	-	0	-	-	-	
CV (%)	-	-	-	0	0	-	-	-	-	0	-	-	-	0	-	-	-	
Ruzi grass [4 WAP, second regrowth, Northern]	-	-	-	59	14.2	-	-	-	-	7.9	-	-	-	57.5	-	-	-	Amole et al., 2022
Average	-	-	-	59	14.2	-	-	-	-	7.9	-	-	-	57.5	-	-	-	Amole et al., 2022 (n=1)
Minimum	-	-	-	59	14.2	-	-	-	-	7.9	-	-	-	57.5	-	-	-	
Maximum	-	-	-	59	14.2	-	-	-	-	7.9	-	-	-	57.5	-	-	-	
Standard deviation	-	-	-	0	0	-	-	-	-	0	-	-	-	0	-	-	-	
CV (%)	-	-	-	0	0	-	-	-	-	0	-	-	-	0	-	-	-	
Ruzi grass [4 WAP, second regrowth, Upper East]	-	-	-	54.1	10.7	-	-	-	-	7.2	-	-	-	55.5	-	-	-	Amole et al., 2022
Average	-	-	-	54.1	10.7	-	-	-	-	7.2	-	-	-	55.5	-	-	-	Amole et al., 2022 (n=1)
Minimum	-	-	-	54.1	10.7	-	-	-	-	7.2	-	-	-	55.5	-	-	-	
Maximum	-	-	-	54.1	10.7	-	-	-	-	7.2	-	-	-	55.5	-	-	-	
Standard deviation	-	-	-	0	0	-	-	-	-	0	-	-	-	0	-	-	-	
CV (%)	-	-	-	0	0	-	-	-	-	0	-	-	-	0	-	-	-	
Ruzi grass [8 WAP, first harvest, Northern]	-	-	-	46.9	8.9	-	-	-	-	7.5	-	-	-	58.2	-	-	-	Amole et al., 2022
Average	-	-	-	46.9	8.9	-	-	-	-	7.5	-	-	-	58.2	-	-	-	Amole et al., 2022 (n=1)
Minimum	-	-	-	46.9	8.9	-	-	-	-	7.5	-	-	-	58.2	-	-	-	
Maximum	-	-	-	46.9	8.9	-	-	-	-	7.5	-	-	-	58.2	-	-	-	
Standard deviation	-	-	-	0	0	-	-	-	-	0	-	-	-	0	-	-	-	
CV (%)	-	-	-	0	0	-	-	-	-	0	-	-	-	0	-	-	-	
Ruzi grass [8 WAP, first harvest, Upper East]	-	-	-	47.9	7.8	-	-	-	-	6.5	-	-	-	61.7	-	-	-	Amole et al., 2022
Average	-	-	-	47.9	7.8	-	-	-	-	6.5	-	-	-	61.7	-	-	-	Amole et al., 2022 (n=1)
Minimum	-	-	-	47.9	7.8	-	-	-	-	6.5	-	-	-	61.7	-	-	-	
Maximum	-	-	-	47.9	7.8	-	-	-	-	6.5	-	-	-	61.7	-	-	-	
Standard deviation	-	-	-	0	0	-	-	-	-	0	-	-	-	0	-	-	-	
CV (%)	-	-	-	0	0	-	-	-	-	0	-	-	-	0	-	-	-	
Ruzi grass [8 WAP, first regrowth, Northern]	-	-	-	47.1	9	-	-	-	-	7.4	-	-	-	58.8	-	-	-	Amole et al., 2022
Average	-	-	-	47.1	9	-	-	-	-	7.4	-	-	-	58.8	-	-	-	Amole et al., 2022 (n=1)
Minimum	-	-	-	47.1	9	-	-	-	-	7.4	-	-	-	58.8	-	-	-	
Maximum	-	-	-	47.1	9	-	-	-	-	7.4	-	-	-	58.8	-	-	-	
Standard deviation	-	-	-	0	0	-	-	-	-	0	-	-	-	0	-	-	-	
CV (%)	-	-	-	0	0	-	-	-	-	0	-	-	-	0	-	-	-	

Ruzi grass [8 WAP, first regrowth, Upper East]	-	-	-	46.8	7.5	-	-	-	-	6.4	-	-	-	60.7	-	-	-	Amole et al., 2022
Average	-	-	-	46.8	7.5	-	-	-	-	6.4	-	-	-	60.7	-	-	-	Amole et al., 2022 (n=1)
Minimum	-	-	-	46.8	7.5	-	-	-	-	6.4	-	-	-	60.7	-	-	-	
Maximum	-	-	-	46.8	7.5	-	-	-	-	6.4	-	-	-	60.7	-	-	-	
Standard deviation	-	-	-	0	0	-	-	-	-	0	-	-	-	0	-	-	-	
CV (%)	-	-	-	0	0	-	-	-	-	0	-	-	-	0	-	-	-	
Ruzi grass [12 WAP, first harvest, Northern]	-	-	-	41.1	8.3	-	-	-	-	7.1	-	-	-	64.2	-	-	-	Amole et al., 2022
Average	-	-	-	41.1	8.3	-	-	-	-	7.1	-	-	-	64.2	-	-	-	Amole et al., 2022 (n=1)
Minimum	-	-	-	41.1	8.3	-	-	-	-	7.1	-	-	-	64.2	-	-	-	
Maximum	-	-	-	41.1	8.3	-	-	-	-	7.1	-	-	-	64.2	-	-	-	
Standard deviation	-	-	-	0	0	-	-	-	-	0	-	-	-	0	-	-	-	
CV (%)	-	-	-	0	0	-	-	-	-	0	-	-	-	0	-	-	-	
Ruzi grass [12 WAP, first harvest, Upper East]	-	-	-	43.6	6.7	-	-	-	-	6.2	-	-	-	65.7	-	-	-	Amole et al., 2022
Average	-	-	-	43.6	6.7	-	-	-	-	6.2	-	-	-	65.7	-	-	-	Amole et al., 2022 (n=1)
Minimum	-	-	-	43.6	6.7	-	-	-	-	6.2	-	-	-	65.7	-	-	-	
Maximum	-	-	-	43.6	6.7	-	-	-	-	6.2	-	-	-	65.7	-	-	-	
Standard deviation	-	-	-	0	0	-	-	-	-	0	-	-	-	0	-	-	-	
CV (%)	-	-	-	0	0	-	-	-	-	0	-	-	-	0	-	-	-	
Ruzi grass [16 WAP, first harvest, Northern]	-	-	-	32.7	6.7	-	-	-	-	6.7	-	-	-	67.8	-	-	-	Amole et al., 2022
Average	-	-	-	32.7	6.7	-	-	-	-	6.7	-	-	-	67.8	-	-	-	Amole et al., 2022 (n=1)
Minimum	-	-	-	32.7	6.7	-	-	-	-	6.7	-	-	-	67.8	-	-	-	
Maximum	-	-	-	32.7	6.7	-	-	-	-	6.7	-	-	-	67.8	-	-	-	
Standard deviation	-	-	-	0	0	-	-	-	-	0	-	-	-	0	-	-	-	
CV (%)	-	-	-	0	0	-	-	-	-	0	-	-	-	0	-	-	-	
Ruzi grass [16 WAP, first harvest, Upper East]	-	-	-	35.5	5.3	-	-	-	-	5.6	-	-	-	67	-	-	-	Amole et al., 2022
Average	-	-	-	35.5	5.3	-	-	-	-	5.6	-	-	-	67	-	-	-	Amole et al., 2022 (n=1)
Minimum	-	-	-	35.5	5.3	-	-	-	-	5.6	-	-	-	67	-	-	-	
Maximum	-	-	-	35.5	5.3	-	-	-	-	5.6	-	-	-	67	-	-	-	
Standard deviation	-	-	-	0	0	-	-	-	-	0	-	-	-	0	-	-	-	
CV (%)	-	-	-	0	0	-	-	-	-	0	-	-	-	0	-	-	-	
Forage sorghum [4 WAP, first harvest, Northern]	-	-	-	62.9	19.7	-	-	-	-	7.4	-	-	-	56.6	-	-	-	Amole et al., 2022
Average	-	-	-	62.9	19.7	-	-	-	-	7.4	-	-	-	56.6	-	-	-	Amole et al., 2022 (n=1)
Minimum	-	-	-	62.9	19.7	-	-	-	-	7.4	-	-	-	56.6	-	-	-	
Maximum	-	-	-	62.9	19.7	-	-	-	-	7.4	-	-	-	56.6	-	-	-	
Standard deviation	-	-	-	0	0	-	-	-	-	0	-	-	-	0	-	-	-	

CV (%)	-	-	-	0	0	-	-	-	-	0	-	-	-	0	-	-	-	
Forage sorghum [4 WAP, first harvest, Upper East]	-	-	-	57.2	19.3	-	-	-	-	7	-	-	-	60.1	-	-	-	Amole et al., 2022
Average	-	-	-	57.2	19.3	-	-	-	-	7	-	-	-	60.1	-	-	-	Amole et al., 2022 (n=1)
Minimum	-	-	-	57.2	19.3	-	-	-	-	7	-	-	-	60.1	-	-	-	
Maximum	-	-	-	57.2	19.3	-	-	-	-	7	-	-	-	60.1	-	-	-	
Standard deviation	-	-	-	0	0	-	-	-	-	0	-	-	-	0	-	-	-	
CV (%)	-	-	-	0	0	-	-	-	-	0	-	-	-	0	-	-	-	
Forage sorghum [4 WAP, first regrowth, Northern]	-	-	-	60.7	10.4	-	-	-	-	7.8	-	-	-	59.4	-	-	-	Amole et al., 2022
Average	-	-	-	60.7	10.4	-	-	-	-	7.8	-	-	-	59.4	-	-	-	Amole et al., 2022 (n=1)
Minimum	-	-	-	60.7	10.4	-	-	-	-	7.8	-	-	-	59.4	-	-	-	
Maximum	-	-	-	60.7	10.4	-	-	-	-	7.8	-	-	-	59.4	-	-	-	
Standard deviation	-	-	-	0	0	-	-	-	-	0	-	-	-	0	-	-	-	
CV (%)	-	-	-	0	0	-	-	-	-	0	-	-	-	0	-	-	-	
Forage sorghum [4 WAP, first regrowth, Upper East]	-	-	-	53.1	8.7	-	-	-	-	6.7	-	-	-	59	-	-	-	Amole et al., 2022
Average	-	-	-	53.1	8.7	-	-	-	-	6.7	-	-	-	59	-	-	-	Amole et al., 2022 (n=1)
Minimum	-	-	-	53.1	8.7	-	-	-	-	6.7	-	-	-	59	-	-	-	
Maximum	-	-	-	53.1	8.7	-	-	-	-	6.7	-	-	-	59	-	-	-	
Standard deviation	-	-	-	0	0	-	-	-	-	0	-	-	-	0	-	-	-	
CV (%)	-	-	-	0	0	-	-	-	-	0	-	-	-	0	-	-	-	
Forage sorghum [4 WAP, second regrowth, Northern]	-	-	-	55.5	7.7	-	-	-	-	7.8	-	-	-	55.6	-	-	-	Amole et al., 2022
Average	-	-	-	55.5	7.7	-	-	-	-	7.8	-	-	-	55.6	-	-	-	Amole et al., 2022 (n=1)
Minimum	-	-	-	55.5	7.7	-	-	-	-	7.8	-	-	-	55.6	-	-	-	
Maximum	-	-	-	55.5	7.7	-	-	-	-	7.8	-	-	-	55.6	-	-	-	
Standard deviation	-	-	-	0	0	-	-	-	-	0	-	-	-	0	-	-	-	
CV (%)	-	-	-	0	0	-	-	-	-	0	-	-	-	0	-	-	-	
Forage sorghum [4 WAP, second regrowth, Upper East]	-	-	-	56.9	7.5	-	-	-	-	6.4	-	-	-	58.9	-	-	-	Amole et al., 2022
Average	-	-	-	56.9	7.5	-	-	-	-	6.4	-	-	-	58.9	-	-	-	Amole et al., 2022 (n=1)
Minimum	-	-	-	56.9	7.5	-	-	-	-	6.4	-	-	-	58.9	-	-	-	
Maximum	-	-	-	56.9	7.5	-	-	-	-	6.4	-	-	-	58.9	-	-	-	
Standard deviation	-	-	-	0	0	-	-	-	-	0	-	-	-	0	-	-	-	
CV (%)	-	-	-	0	0	-	-	-	-	0	-	-	-	0	-	-	-	
Forage sorghum [8 WAP, first harvest, Northern]	-	-	-	46.8	8.9	-	-	-	-	7.5	-	-	-	60.3	-	-	-	Amole et al., 2022

Average	-	-	-	46.8	8.9	-	-	-	-	7.5	-	-	-	60.3	-	-	-	Amole et al., 2022 (n=1)
Minimum	-	-	-	46.8	8.9	-	-	-	-	7.5	-	-	-	60.3	-	-	-	
Maximum	-	-	-	46.8	8.9	-	-	-	-	7.5	-	-	-	60.3	-	-	-	
Standard deviation	-	-	-	0	0	-	-	-	-	0	-	-	-	0	-	-	-	
CV (%)	-	-	-	0	0	-	-	-	-	0	-	-	-	0	-	-	-	
Forage sorghum [8 WAP, first harvest, Upper East]	-	-	-	45.2	7.5	-	-	-	-	6.6	-	-	-	63.9	-	-	-	Amole et al., 2022
Average	-	-	-	45.2	7.5	-	-	-	-	6.6	-	-	-	63.9	-	-	-	Amole et al., 2022 (n=1)
Minimum	-	-	-	45.2	7.5	-	-	-	-	6.6	-	-	-	63.9	-	-	-	
Maximum	-	-	-	45.2	7.5	-	-	-	-	6.6	-	-	-	63.9	-	-	-	
Standard deviation	-	-	-	0	0	-	-	-	-	0	-	-	-	0	-	-	-	
CV (%)	-	-	-	0	0	-	-	-	-	0	-	-	-	0	-	-	-	
Forage sorghum [8 WAP, first regrowth, Northern]	-	-	-	47.1	8.9	-	-	-	-	7.4	-	-	-	60.2	-	-	-	Amole et al., 2022
Average	-	-	-	47.1	8.9	-	-	-	-	7.4	-	-	-	60.2	-	-	-	Amole et al., 2022 (n=1)
Minimum	-	-	-	47.1	8.9	-	-	-	-	7.4	-	-	-	60.2	-	-	-	
Maximum	-	-	-	47.1	8.9	-	-	-	-	7.4	-	-	-	60.2	-	-	-	
Standard deviation	-	-	-	0	0	-	-	-	-	0	-	-	-	0	-	-	-	
CV (%)	-	-	-	0	0	-	-	-	-	0	-	-	-	0	-	-	-	
Forage sorghum [8 WAP, first regrowth, Upper East]	-	-	-	46.1	7.3	-	-	-	-	6.5	-	-	-	65.4	-	-	-	Amole et al., 2022
Average	-	-	-	46.1	7.3	-	-	-	-	6.5	-	-	-	65.4	-	-	-	Amole et al., 2022 (n=1)
Minimum	-	-	-	46.1	7.3	-	-	-	-	6.5	-	-	-	65.4	-	-	-	
Maximum	-	-	-	46.1	7.3	-	-	-	-	6.5	-	-	-	65.4	-	-	-	
Standard deviation	-	-	-	0	0	-	-	-	-	0	-	-	-	0	-	-	-	
CV (%)	-	-	-	0	0	-	-	-	-	0	-	-	-	0	-	-	-	
Forage sorghum [12 WAP, first harvest, Northern]	-	-	-	40.1	9.2	-	-	-	-	6	-	-	-	66.8	-	-	-	Amole et al., 2022
Average	-	-	-	40.1	9.2	-	-	-	-	6	-	-	-	66.8	-	-	-	Amole et al., 2022 (n=1)
Minimum	-	-	-	40.1	9.2	-	-	-	-	6	-	-	-	66.8	-	-	-	
Maximum	-	-	-	40.1	9.2	-	-	-	-	6	-	-	-	66.8	-	-	-	
Standard deviation	-	-	-	0	0	-	-	-	-	0	-	-	-	0	-	-	-	
CV (%)	-	-	-	0	0	-	-	-	-	0	-	-	-	0	-	-	-	
Forage sorghum [12 WAP, first harvest, Upper East]	-	-	-	40.7	6.4	-	-	-	-	5.6	-	-	-	66.8	-	-	-	Amole et al., 2022
Average	-	-	-	40.7	6.4	-	-	-	-	5.6	-	-	-	66.8	-	-	-	Amole et al., 2022 (n=1)
Minimum	-	-	-	40.7	6.4	-	-	-	-	5.6	-	-	-	66.8	-	-	-	
Maximum	-	-	-	40.7	6.4	-	-	-	-	5.6	-	-	-	66.8	-	-	-	
Standard deviation	-	-	-	0	0	-	-	-	-	0	-	-	-	0	-	-	-	
CV (%)	-	-	-	0	0	-	-	-	-	0	-	-	-	0	-	-	-	

Forage sorghum [16 WAP, first harvest, Northern]	-	-	-	32.3	6.9	-	-	-	-	6.5	-	-	-	69.4	-	-	-	Amole et al., 2022
Average	-	-	-	32.3	6.9	-	-	-	-	6.5	-	-	-	69.4	-	-	-	Amole et al., 2022 (n=1)
Minimum	-	-	-	32.3	6.9	-	-	-	-	6.5	-	-	-	69.4	-	-	-	
Maximum	-	-	-	32.3	6.9	-	-	-	-	6.5	-	-	-	69.4	-	-	-	
Standard deviation	-	-	-	0	0	-	-	-	-	0	-	-	-	0	-	-	-	
CV (%)	-	-	-	0	0	-	-	-	-	0	-	-	-	0	-	-	-	
Forage sorghum [16 WAP, first harvest, Upper East]	-	-	-	35.3	5	-	-	-	-	5.3	-	-	-	70.9	-	-	-	Amole et al., 2022
Average	-	-	-	35.3	5	-	-	-	-	5.3	-	-	-	70.9	-	-	-	Amole et al., 2022 (n=1)
Minimum	-	-	-	35.3	5	-	-	-	-	5.3	-	-	-	70.9	-	-	-	
Maximum	-	-	-	35.3	5	-	-	-	-	5.3	-	-	-	70.9	-	-	-	
Standard deviation	-	-	-	0	0	-	-	-	-	0	-	-	-	0	-	-	-	
CV (%)	-	-	-	0	0	-	-	-	-	0	-	-	-	0	-	-	-	
Aspilia africana [rural]	87.24	5.73	85.63	-	13.63	10.74	14.37	-	-	-	-	-	-	-	-	-	-	Okoli et al., 2024
Average	87.24	5.73	85.63	-	13.63	10.74	14.37	-	-	-	-	-	-	-	-	-	-	Okoli et al., 2024 (n=1)
Minimum	87.24	5.73	85.63	-	13.63	10.74	14.37	-	-	-	-	-	-	-	-	-	-	
Maximum	87.24	5.73	85.63	-	13.63	10.74	14.37	-	-	-	-	-	-	-	-	-	-	
Standard deviation	0	0	0	-	0	0	0	-	-	-	-	-	-	-	-	-	-	
CV (%)	0	0	0	-	0	0	0	-	-	-	-	-	-	-	-	-	-	
Aspilia africana [peri-urban]	87.35	0.96	94.85	-	5.97	22.65	5.15	-	-	-	-	-	-	-	-	-	-	Okoli et al., 2024
Average	87.35	0.96	94.85	-	5.97	22.65	5.15	-	-	-	-	-	-	-	-	-	-	Okoli et al., 2024 (n=1)
Minimum	87.35	0.96	94.85	-	5.97	22.65	5.15	-	-	-	-	-	-	-	-	-	-	
Maximum	87.35	0.96	94.85	-	5.97	22.65	5.15	-	-	-	-	-	-	-	-	-	-	
Standard deviation	0	0	0	-	0	0	0	-	-	-	-	-	-	-	-	-	-	
CV (%)	0	0	0	-	0	0	0	-	-	-	-	-	-	-	-	-	-	
Vernonia amygdalina [rural]	87.47	4.96	89.65	-	15.17	6.94	10.35	-	-	-	-	-	-	-	-	-	-	Okoli et al., 2024
Average	87.47	4.96	89.65	-	15.17	6.94	10.35	-	-	-	-	-	-	-	-	-	-	Okoli et al., 2024 (n=1)
Minimum	87.47	4.96	89.65	-	15.17	6.94	10.35	-	-	-	-	-	-	-	-	-	-	
Maximum	87.47	4.96	89.65	-	15.17	6.94	10.35	-	-	-	-	-	-	-	-	-	-	
Standard deviation	0	0	0	-	0	0	0	-	-	-	-	-	-	-	-	-	-	
CV (%)	0	0	0	-	0	0	0	-	-	-	-	-	-	-	-	-	-	
Costus afer [rural]	86.85	1.66	90.95	-	9	13.53	9.05	-	-	-	-	-	-	-	-	-	-	Okoli et al., 2024
Average	86.85	1.66	90.95	-	9	13.53	9.05	-	-	-	-	-	-	-	-	-	-	Okoli et al., 2024 (n=1)
Minimum	86.85	1.66	90.95	-	9	13.53	9.05	-	-	-	-	-	-	-	-	-	-	
Maximum	86.85	1.66	90.95	-	9	13.53	9.05	-	-	-	-	-	-	-	-	-	-	
Standard deviation	0	0	0	-	0	0	0	-	-	-	-	-	-	-	-	-	-	
CV (%)	0	0	0	-	0	0	0	-	-	-	-	-	-	-	-	-	-	

Musa paradisiaca [peri-urban]	87.94	1.47	88.13	-	9.09	26.15	11.87	-	-	-	-	-	-	-	-	-	-	Okoli et al., 2024
Average	87.94	1.47	88.13	-	9.09	26.15	11.87	-	-	-	-	-	-	-	-	-	-	Okoli et al., 2024 (n=1)
Minimum	87.94	1.47	88.13	-	9.09	26.15	11.87	-	-	-	-	-	-	-	-	-	-	
Maximum	87.94	1.47	88.13	-	9.09	26.15	11.87	-	-	-	-	-	-	-	-	-	-	
Standard deviation	0	0	0	-	0	0	0	-	-	-	-	-	-	-	-	-	-	
CV (%)	0	0	0	-	0	0	0	-	-	-	-	-	-	-	-	-	-	
Bambusa tulda [peri-urban]	88.46	2.04	89.66	-	10.14	10.16	10.34	-	-	-	-	-	-	-	-	-	-	Okoli et al., 2024
Average	88.46	2.04	89.66	-	10.14	10.16	10.34	-	-	-	-	-	-	-	-	-	-	Okoli et al., 2024 (n=1)
Minimum	88.46	2.04	89.66	-	10.14	10.16	10.34	-	-	-	-	-	-	-	-	-	-	
Maximum	88.46	2.04	89.66	-	10.14	10.16	10.34	-	-	-	-	-	-	-	-	-	-	
Standard deviation	0	0	0	-	0	0	0	-	-	-	-	-	-	-	-	-	-	
CV (%)	0	0	0	-	0	0	0	-	-	-	-	-	-	-	-	-	-	
Acroceras amplectens	-	-	-	-	2.25	8.8	-	-	-	-	-	-	-	-	-	0.03	0.037	Amegnaglo et al., 2018
Average	-	-	-	-	2.25	8.8	-	-	-	-	-	-	-	-	-	0.03	0.04	Amegnaglo et al., 2018 (n=1)
Minimum	-	-	-	-	2.25	8.8	-	-	-	-	-	-	-	-	-	0.03	0.04	
Maximum	-	-	-	-	2.25	8.8	-	-	-	-	-	-	-	-	-	0.03	0.04	
Standard deviation	-	-	-	-	0	0	-	-	-	-	-	-	-	-	-	0	0	
CV (%)	-	-	-	-	0	0	-	-	-	-	-	-	-	-	-	0	0	
Andropogon africana	-	-	-	-	1.56	8.6	-	-	-	-	-	-	-	-	-	0.067	0.04	Amegnaglo et al., 2018
Average	-	-	-	-	1.56	8.6	-	-	-	-	-	-	-	-	-	0.07	0.04	Amegnaglo et al., 2018 (n=1)
Minimum	-	-	-	-	1.56	8.6	-	-	-	-	-	-	-	-	-	0.07	0.04	
Maximum	-	-	-	-	1.56	8.6	-	-	-	-	-	-	-	-	-	0.07	0.04	
Standard deviation	-	-	-	-	0	0	-	-	-	-	-	-	-	-	-	0	0	
CV (%)	-	-	-	-	0	0	-	-	-	-	-	-	-	-	-	0	0	
Brachiaria mutica	-	-	-	-	1.94	7.9	-	-	-	-	-	-	-	-	-	0.035	0.05	Amegnaglo et al., 2018
Average	-	-	-	-	1.94	7.9	-	-	-	-	-	-	-	-	-	0.04	0.05	Amegnaglo et al., 2018 (n=1)
Minimum	-	-	-	-	1.94	7.9	-	-	-	-	-	-	-	-	-	0.04	0.05	
Maximum	-	-	-	-	1.94	7.9	-	-	-	-	-	-	-	-	-	0.04	0.05	
Standard deviation	-	-	-	-	0	0	-	-	-	-	-	-	-	-	-	0	0	
CV (%)	-	-	-	-	0	0	-	-	-	-	-	-	-	-	-	0	0	
Cynodon dactylon	-	-	-	-	4.5	9.1	-	-	-	-	-	-	-	-	-	0.041	0.06	Amegnaglo et al., 2018
Average	-	-	-	-	4.5	9.1	-	-	-	-	-	-	-	-	-	0.04	0.06	Amegnaglo et al., 2018 (n=1)
Minimum	-	-	-	-	4.5	9.1	-	-	-	-	-	-	-	-	-	0.04	0.06	
Maximum	-	-	-	-	4.5	9.1	-	-	-	-	-	-	-	-	-	0.04	0.06	
Standard deviation	-	-	-	-	0	0	-	-	-	-	-	-	-	-	-	0	0	
CV (%)	-	-	-	-	0	0	-	-	-	-	-	-	-	-	-	0	0	
Digitaria leptocharis	-	-	-	-	5.56	9.9	-	-	-	-	-	-	-	-	-	0.037	0.035	Amegnaglo et al., 2018
Average	-	-	-	-	5.56	9.9	-	-	-	-	-	-	-	-	-	0.04	0.04	Amegnaglo et al., 2018 (n=1)
Minimum	-	-	-	-	5.56	9.9	-	-	-	-	-	-	-	-	-	0.04	0.04	
Maximum	-	-	-	-	5.56	9.9	-	-	-	-	-	-	-	-	-	0.04	0.04	
Standard deviation	-	-	-	-	0	0	-	-	-	-	-	-	-	-	-	0	0	
CV (%)	-	-	-	-	0	0	-	-	-	-	-	-	-	-	-	0	0	

Diheteropogon amplectens	-	-	-	-	2.13	10	-	-	-	-	-	-	-	-	-	0.048	0.34	Amegnaglo et al., 2018
Average	-	-	-	-	2.13	10	-	-	-	-	-	-	-	-	-	0.05	0.34	Amegnaglo et al., 2018 (n=1)
Minimum	-	-	-	-	2.13	10	-	-	-	-	-	-	-	-	-	0.05	0.34	
Maximum	-	-	-	-	2.13	10	-	-	-	-	-	-	-	-	-	0.05	0.34	
Standard deviation	-	-	-	-	0	0	-	-	-	-	-	-	-	-	-	0	0	
CV (%)	-	-	-	-	0	0	-	-	-	-	-	-	-	-	-	0	0	
Diheteropogon hagerupii	-	-	-	-	5.06	12	-	-	-	-	-	-	-	-	-	0.07	0.039	Amegnaglo et al., 2018
Average	-	-	-	-	5.06	12	-	-	-	-	-	-	-	-	-	0.07	0.04	Amegnaglo et al., 2018 (n=1)
Minimum	-	-	-	-	5.06	12	-	-	-	-	-	-	-	-	-	0.07	0.04	
Maximum	-	-	-	-	5.06	12	-	-	-	-	-	-	-	-	-	0.07	0.04	
Standard deviation	-	-	-	-	0	0	-	-	-	-	-	-	-	-	-	0	0	
CV (%)	-	-	-	-	0	0	-	-	-	-	-	-	-	-	-	0	0	
Leersia hexandra	-	-	-	-	5.19	10	-	-	-	-	-	-	-	-	-	0.042	0.042	Amegnaglo et al., 2018
Average	-	-	-	-	5.19	10	-	-	-	-	-	-	-	-	-	0.04	0.04	Amegnaglo et al., 2018 (n=1)
Minimum	-	-	-	-	5.19	10	-	-	-	-	-	-	-	-	-	0.04	0.04	
Maximum	-	-	-	-	5.19	10	-	-	-	-	-	-	-	-	-	0.04	0.04	
Standard deviation	-	-	-	-	0	0	-	-	-	-	-	-	-	-	-	0	0	
CV (%)	-	-	-	-	0	0	-	-	-	-	-	-	-	-	-	0	0	
Panicum phragmitoides	-	-	-	-	5.19	15	-	-	-	-	-	-	-	-	-	0.026	0.03	Amegnaglo et al., 2018
Average	-	-	-	-	5.19	15	-	-	-	-	-	-	-	-	-	0.03	0.03	Amegnaglo et al., 2018 (n=1)
Minimum	-	-	-	-	5.19	15	-	-	-	-	-	-	-	-	-	0.03	0.03	
Maximum	-	-	-	-	5.19	15	-	-	-	-	-	-	-	-	-	0.03	0.03	
Standard deviation	-	-	-	-	0	0	-	-	-	-	-	-	-	-	-	0	0	
CV (%)	-	-	-	-	0	0	-	-	-	-	-	-	-	-	-	0	0	
Paspalum orbiculare	-	-	-	-	3.5	7.9	-	-	-	-	-	-	-	-	-	0.129	0.062	Amegnaglo et al., 2018
Average	-	-	-	-	3.5	7.9	-	-	-	-	-	-	-	-	-	0.13	0.06	Amegnaglo et al., 2018 (n=1)
Minimum	-	-	-	-	3.5	7.9	-	-	-	-	-	-	-	-	-	0.13	0.06	
Maximum	-	-	-	-	3.5	7.9	-	-	-	-	-	-	-	-	-	0.13	0.06	
Standard deviation	-	-	-	-	0	0	-	-	-	-	-	-	-	-	-	0	0	
CV (%)	-	-	-	-	0	0	-	-	-	-	-	-	-	-	-	0	0	
Pennisetum polystachyon	-	-	-	-	3.5	16	-	-	-	-	-	-	-	-	-	0.046	0.038	Amegnaglo et al., 2018
Average	-	-	-	-	3.5	16	-	-	-	-	-	-	-	-	-	0.05	0.04	Amegnaglo et al., 2018 (n=1)
Minimum	-	-	-	-	3.5	16	-	-	-	-	-	-	-	-	-	0.05	0.04	
Maximum	-	-	-	-	3.5	16	-	-	-	-	-	-	-	-	-	0.05	0.04	
Standard deviation	-	-	-	-	0	0	-	-	-	-	-	-	-	-	-	0	0	
CV (%)	-	-	-	-	0	0	-	-	-	-	-	-	-	-	-	0	0	
Vetiveria nigritana	-	-	-	-	3.63	6.3	-	-	-	-	-	-	-	-	-	0.027	0.05	Amegnaglo et al., 2018
Average	-	-	-	-	3.63	6.3	-	-	-	-	-	-	-	-	-	0.03	0.05	Amegnaglo et al., 2018 (n=1)
Minimum	-	-	-	-	3.63	6.3	-	-	-	-	-	-	-	-	-	0.03	0.05	
Maximum	-	-	-	-	3.63	6.3	-	-	-	-	-	-	-	-	-	0.03	0.05	
Standard deviation	-	-	-	-	0	0	-	-	-	-	-	-	-	-	-	0	0	
CV (%)	-	-	-	-	0	0	-	-	-	-	-	-	-	-	-	0	0	
